# Design of a mucin-selective protease for targeted degradation of cancer-associated mucins

**DOI:** 10.1038/s41587-023-01840-6

**Published:** 2023-08-03

**Authors:** Kayvon Pedram, D. Judy Shon, Gabrielle S. Tender, Natalia R. Mantuano, Jason J. Northey, Kevin J. Metcalf, Simon P. Wisnovsky, Nicholas M. Riley, Giovanni C. Forcina, Stacy A. Malaker, Angel Kuo, Benson M. George, Caitlyn L. Miller, Kerriann M. Casey, José G. Vilches-Moure, Michael J. Ferracane, Valerie M. Weaver, Heinz Läubli, Carolyn R. Bertozzi

**Affiliations:** 1 Department of Chemistry and Sarafan ChEM-H, Stanford University, Stanford, CA, USA.; 2 Cancer Immunotherapy Laboratory, Department of Biomedicine, University of Basel, Basel, Switzerland.; 3 Division of Oncology, Department of Theragnostics, University Hospital, Basel, Switzerland.; 4 Center for Bioengineering and Tissue Regeneration, Department of Surgery, University of California, San Francisco (UCSF), San Francisco, CA, USA.; 5 Institute for Stem Cell Biology and Regenerative Medicine, Stanford University School of Medicine, Stanford, CA, USA.; 6 Ludwig Center for Cancer Stem Cell Research and Medicine, Stanford University School of Medicine, Stanford, CA, USA.; 7 Department of Comparative Medicine, Stanford University, Stanford, CA, USA.; 8 Department of Chemistry, University of Redlands, Redlands, CA, USA.; 9 Departments of Radiation Oncology and Bioengineering and Therapeutic Sciences, Eli and Edythe Broad Center of Regeneration Medicine and Stem Cell Research, and Helen Diller Comprehensive Cancer Center, University of California, San Francisco (UCSF), San Francisco, CA, USA.; 10 Howard Hughes Medical Institute, Stanford, CA, USA.; 11 Present address: Janelia Research Campus, Howard Hughes Medical Institute, Ashburn, VA, USA.; 12 Present address: Faculty of Pharmaceutical Sciences, University of British Columbia, Vancouver, British Columbia, Canada.; 13 Present address: Department of Chemistry, Yale University, New Haven, CT, USA.; 14 Present address: Brigham and Women’s Hospital, Boston, MA, USA.; 15 These authors contributed equally: Kayvon Pedram, D. Judy Shon, Gabrielle S. Tender.

## Abstract

Targeted protein degradation is an emerging strategy for the elimination of classically undruggable proteins. Here, to expand the landscape of targetable substrates, we designed degraders that achieve substrate selectivity via recognition of a discrete peptide and glycan motif and achieve cell-type selectivity via antigen-driven cell-surface binding. We applied this approach to mucins, *O*-glycosylated proteins that drive cancer progression through biophysical and immunological mechanisms. Engineering of a bacterial mucin-selective protease yielded a variant for fusion to a cancer antigen-binding nanobody. The resulting conjugate selectively degraded mucins on cancer cells, promoted cell death in culture models of mucin-driven growth and survival, and reduced tumor growth in mouse models of breast cancer progression. This work establishes a blueprint for the development of biologics that degrade specific protein glycoforms on target cells.

Mucins are glycoproteins that bear a high density of *O*-glycosylated serine and threonine residues. In species ranging from sea sponges to mammals, mucins are expressed at epithelial and endothelial surfaces where they defend against physical insults and pathogens^1^. The mechanisms by which mucins exert their functions at these surfaces fall broadly into two categories (Fig. 1a). First, mucins are critical to the initiation and propagation of biophysical signals. For example, their extended and rigid secondary structure enables their use by cells as force-sensitive antennae, as is the case for the mucin MUC1 during integrin-mediated adhesion to soft matrices^2^ and the mucin CD45 during macrophage pinocytosis^3^. Second, the glycopeptide epitopes presented by mucins act as ligands for various receptors, particularly those involved in cell adhesion and immune modulation^4^. For example, the immune cell receptor sialic acid-binding immunoglobulin-like lectin 7 (Siglec-7) binds the mucin CD43 on leukemia cell surfaces and delivers an immune inhibitory signal, analogous to classical checkpoint receptors such as PD-1 (ref. 5).

Cancers, especially carcinomas, hijack mucin signaling pathways to protect themselves from both biophysical and immunological insults. It is estimated that just one member of the mucin family, MUC1, is aberrantly expressed in more than half of carcinomas diagnosed per year in the United States^6^, a frequency matched by prototypical oncogenes such as *RAS* and *MYC*. In addition, common carcinomas, such as breast, ovarian and intestinal cancers, have mucinous forms, wherein tumor cells present as individual colonies suspended in a matrix of secreted mucin and polysaccharides^7^. As such, mucins have been exploited as tumor-enriched epitopes for antibodies^8^, antibody–drug conjugates^9^ and chimeric antigen receptor T cells^10^. In addition, anti- bodies, small molecules and peptides targeting the C-terminus of the MUC1 protein are under development to block its well-characterized cancer-driving functions, for example, by preventing dimerization^11^.

Generally, these mucin-targeted therapeutic interventions face the challenge that mucin signaling occurs through the cooperative action of hundreds of arrayed epitopes, a unique scaffolding secondary structure and a C-terminus capable of downstream growth and survival signaling. Therefore, it is attractive to consider strategies that enable therapeutic degradation of overexpressed mucins to reverse their pleiotropic tumor-progressive functions. Depletion of cellular mucins has hitherto only been achieved in the context of mucin 1 kidney disease, wherein a frameshifted and truncated form of MUC1 accumulates in early secretory compartments. This intracellular accumulation can be reversed with a small molecule that binds a cargo receptor, TMED9 (ref. 12).

Targeted protein degradation (TPD) has emerged as a powerful technique to address canonically undruggable targets. Classically, TPD uses bispecific molecules to traffic unwanted proteins to endogenous cellular proteolytic machinery for degradation. An advantage of this approach is that the aberrant protein is deleted, meaning that the full range of its pleiotropic effects on cell signaling are reversed^13^. As conventional TPD relies on proteasomal degradation, it is limited to targets that (1) can be bound with a bridging molecule that recruits endogenous degradation machinery and (2) contain cytosolic domains. Recently, cytosolic delivery of an exogenous, target-selective protease achieved proteolytic manipulation of cytosolic proteins without the need to recruit proteasome-shuttling pathways^14^, and leveraging of lysosomal-shuttling receptors has enabled TPD of cell-surface and secreted proteins^15^.

To degrade cancer-associated mucins, we developed a strategy for degradation of cell-surface proteins on specific cells without the need for endogenous degradation machinery. Specifically, a protease with dual glycan- and peptide-based selectivity for mucins is targeted to cancer cells via fusion to a camelid heavy chain variable domain (nanobody). We demonstrate that these targeted proteases reduce cancer cell viability in cellulo and blunt primary tumor burden and metastatic outgrowth in mouse breast cancer models. As nearly all extracellular proteins are glycosylated^16^ and glycosylation status is commonly altered in disease^4^, glycoform-dependent and cell-type-selective TPD presents a general opportunity for increasing on-target specificity for disease-driving extracellular proteins.

## Results

### Mucinase treatment undermines mucin-driven survival in cells

We and others have characterized proteases from the bacterial kingdom with selectivity for mucin domains^17–21^. These ‘mucinases’ act through recognition of joint peptide and glycan motifs, which have been mapped using mass spectrometry (MS) of cleavage products. As an initial candidate for therapeutic repurposing, we chose the zinc metalloprotease StcE from *Escherichia coli* serotype O157:H7. StcE recognizes the motif S/T*-X-S/T, where the first serine/threonine must bear an *O*-glycan (asterisk) for cleavage to occur^18^. StcE is agnostic to the structure of the glycan and the identity of the X amino acid, which can also be absent. StcE is therefore a pan-mucinase, which is able to act on epitopes present across the natural mucins.

**Fig. 1 |:**
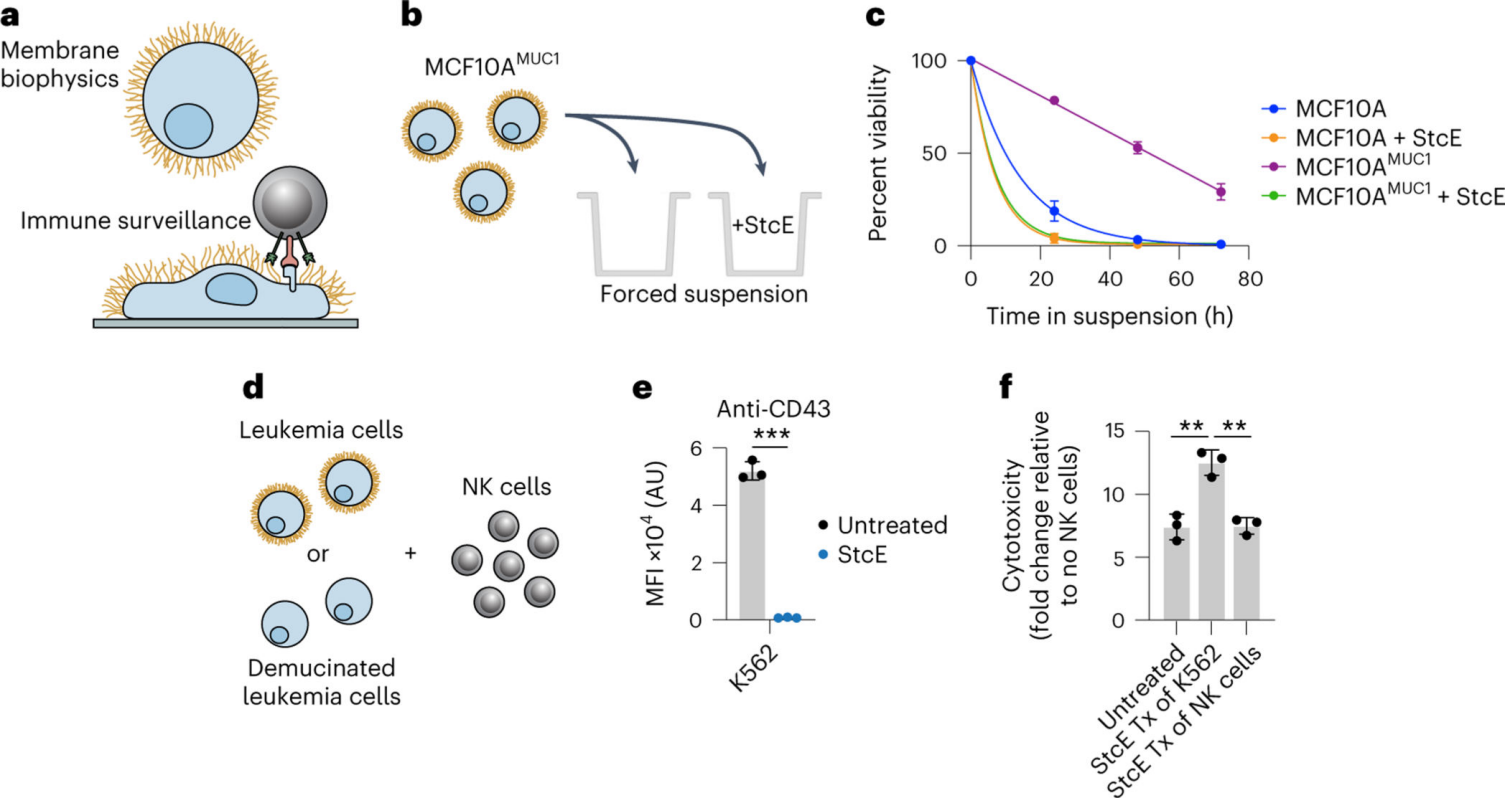
Mucinase treatment reverses mucin-driven survival pathways in cancer cell lines. **a**, Schematic depicting how mucins influence membrane biophysics and immune surveillance. **b**, Setup for the suspension survival assay under anchorage-free conditions using MCF10A cells expressing a doxycycline-inducible MUC1 ectodomain and treated with or without StcE mucinase. **c**, Viability of anchorage-free MCF10A^±MUC1^ cells ± 10 nM StcE over 72 h as determined by flow cytometry (*n* = 3 biologically independent replicates). **d**, Setup for immune surveillance assay with leukemia cell lines ± StcE and primary human NK cells. **e**, Surface CD43 levels of K562 cells ± 50 nM StcE measured by flow cytometry (*n* = 3 biologically independent replicates); MFI, mean fluorescence intensity; AU, arbitrary units. **f**, Normalized NK cell killing of K562 cells under indicated StcE treatment (Tx) conditions at a 2:1 effector:target ratio as determined by flow cytometry (*n* = 3 biologically independent replicates). Data are shown as mean ± s.e.m. (**c**) or mean ± s.d. (**e** and **f**). *P* values were determined by using Tukey-corrected one-way analysis of variance (ANOVA); **P < 0.005; ***P < 0.0005.

To begin, we tested whether treatment with StcE could reduce cell viability by undermining the biophysical function of mucins. Expression of the MUC1 ectodomain in mammary epithelial cells induces a bulky glycocalyx, which causes the cells to lift from their basement membrane and thrive in suspension in a manner characteristic of circulating metastatic tumor cells^22^. To model suspension survival in cellulo, wild-type cells and cells overexpressing MUC1 were plated on ultralow-attachment plates treated with or without StcE and analyzed by flow cytometry to assess viability over 3 d (Fig. 1b). Under these anchorage-free conditions, StcE treatment resulted in rapid cell death, consistent with previously reported alterations in membrane biophysical signaling through PI3K–Akt (Fig. 1c)^23^. Meanwhile, StcE treatment of MUC1-expressing cells in standard tissue culture plates caused suspended cells to settle, after which they continued to divide, highlighting the low toxicity of mucinase treatment at nanomolar doses (Supplemental Videos 1 and 2).

**Extended Data Fig. 1 |:**
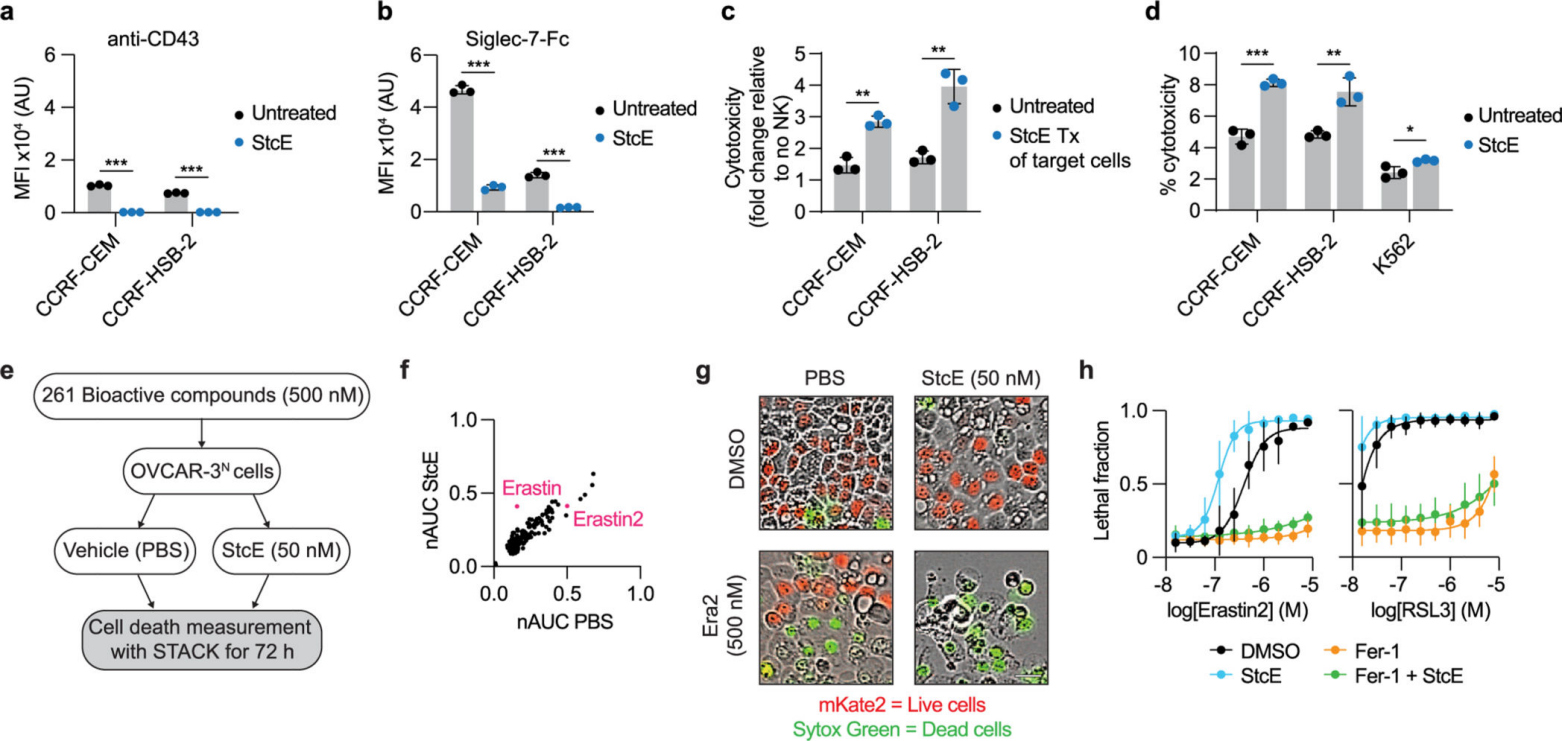
StcE treatment of cell lines potentiates NK cell surveillance and small molecule-induced ferroptosis. **a-b**, Surface CD43 (**a**) and Siglec-7 ligand (**b**) levels of CCRF-CEM and CCRF-HSB-2 cells ± 50 nM StcE measured by flow cytometry (*n* = 3 biologically independent replicates). Siglec-7-Fc staining of K562 cells can be found in Wisnovsky et al. (2021)^5^. MFI = mean fluorescence intensity and AU = arbitrary units. **c**, Normalized NK cell killing of CCRF-CEM and CCRF-HSB-2 cells ± 10 nM StcE at 5:1 and 2:1 effector:target ratios, respectively, as determined by flow cytometry (*n* = 3 biologically independent replicates). Tx = treatment. **d**, Cytotoxicity of StcE treatment of leukemia cell lines (*n* = 3 biologically independent replicates). A statistically significant baseline toxicity increase was observed. **e**, Screening strategy for bioactive compound library on OVCAR-3^N^ cells ± StcE. Superscript *N* denotes stable expression of nuclear fluorescent protein. **f**, Normalized area-under-the-curve (nAUC) of lethal fraction scores of OVCAR-3^N^ cells treated with 500 nM bioactive compounds ± 50 nM StcE (*n* = 2 biologically independent replicates). Ferroptosis-inducing erastin and erastin2 are highlighted in pink. **g**, Visualization of live (red) and dead (green) OVCAR-3^N^ cells ± 50 nM StcE ± 500 nM erastin2 (Era2) at 72 hours, representative images derived from (**f**). Scale bar, 30 μm. **h**, Lethal fraction curves of OVCAR-3^N^ cells treated with 500 nM erastin2 or RSL3 ± 50 nM StcE ± 1 μM ferrostatin-1 (Fer-1) for 48 hours (*n* = 5 biologically independent replicates). Data are mean ± s.d. *P*-values were determined using Tukey-corrected two-way ANOVA. *p < 0.05, **p < 0.005, ***p < 0.0005.

Next, we asked whether StcE treatment could enhance immune surveillance of cancer cells. The mucin CD43 has been identified as a ligand on leukemia cells for the natural killer (NK) cell immune checkpoint receptor Siglec-7 (ref. 5). In this model, removal of CD43 potentiates NK cell killing of leukemia cell lines. To assess whether mucinase treatment would have a similar effect, we treated three leukemia cell lines with or without endotoxin-free StcE (Methods), incubated them with healthy human blood donor NK cells, and quantified viability after 4 h (Fig. 1d). StcE treatment resulted in loss of cell-surface CD43 and overall Siglec-7 ligand residency, as expected (Fig. 1e and Extended Data Fig. 1a,b)^5^. Demucinated leukemia cells were susceptible to increased NK cell surveillance, consistent with a recent report showing augmentation of breast cancer cell line killing by immortalized NK cells after mucinase treatment (Fig. 1f and Extended Data Fig. 1c,d)^24^.

As the presence of mucins on cell surfaces has been associated with decreased drug efficacy^25^, we also explored whether mucinase treatment would synergize with small-molecule cytotoxic drugs. Using scalable time-lapse analysis of cell death kinetics (STACK)^26^, we screened a 261-compound library in an ovarian cancer cell line (Extended Data Fig. 1e). The commercial library of 261 small molecules targeted a range of biological pathways (Supplemental Table 1) and had been used previously to evaluate treatments that modulate compound cytotoxicity^27^. Erastin, which induces ferroptosis through inhibition of the cystine:glutamate antiporter system x_c_^−^, scored among the top hits for compound cell death enhanced by StcE treatment (Extended Data Fig. 1f,g and Supplemental Table 1)^28^. A dose response with erastin2, a more potent analog, confirmed enhancement of ferroptosis with StcE cotreatment that was fully suppressed by the ferroptosis inhibitor ferrostatin-1. By contrast, there was no enhancement of ferroptosis induced by the mechanistically distinct compound RSL3 (Extended Data Fig. 1h)^29^. Taken together, these results demonstrate that removal of mucins via mucinase treatment can reverse their pleiotropic tumor-progressive roles.

### Toxicity profile of StcE necessitates tumor targeting

**Extended Data Fig. 2 |:**
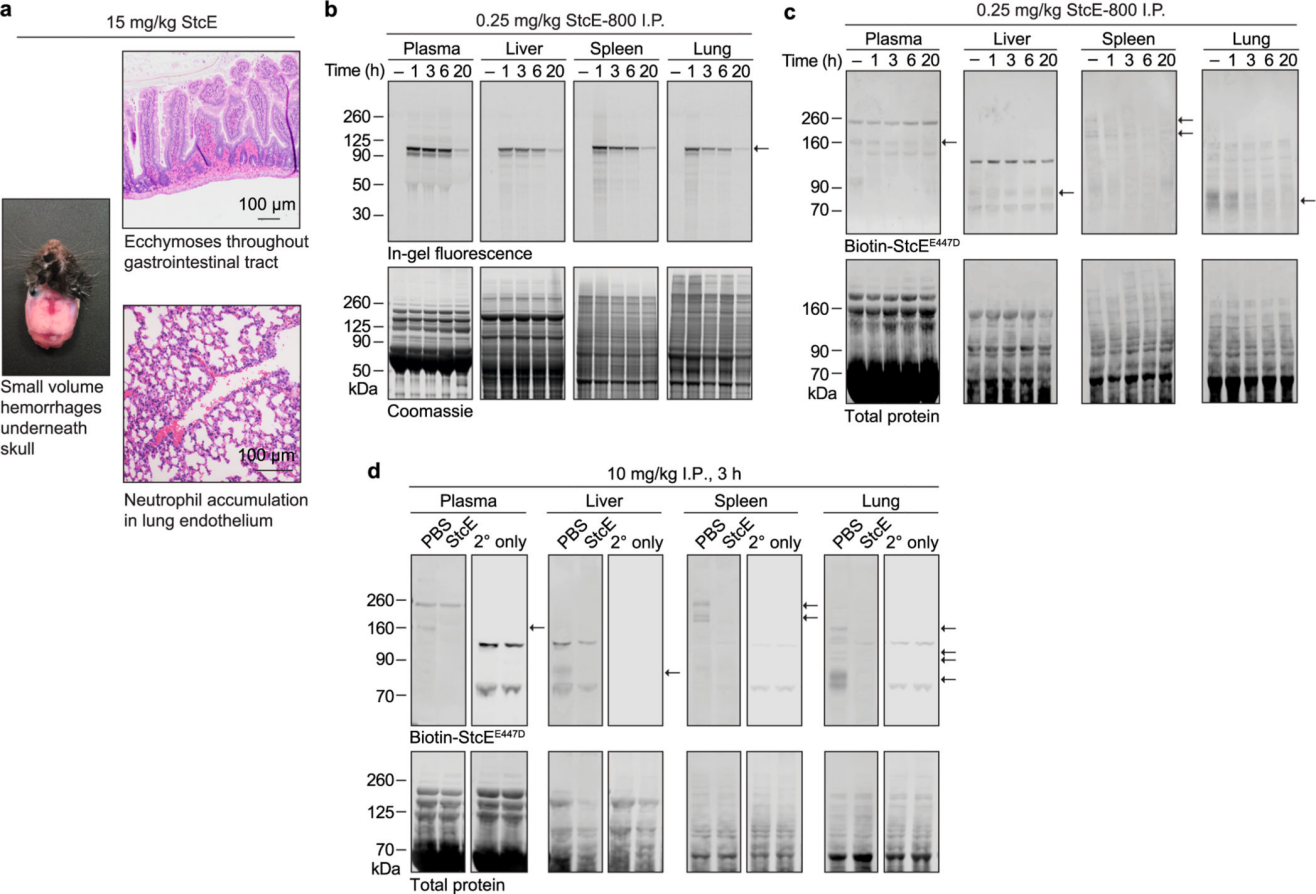
StcE cleaves mucins in mouse tissues at a maximum tolerated dose of 0.25 mg/kg and exhibits systemic toxicity at higher doses. **a**, Necropsy analysis post intravenous (I.V.) injection of 15 mg/kg StcE revealed abnormalities in the lung, gastrointestinal tract, and underneath the skull (*n* = 1 animal). **b**, SDS-PAGE of plasma and tissues post intraperitoneal (I.P.) injection of PBS or 0.25 mg/kg of IRdye 800CW-labeled StcE (StcE-800, molecular weight = 98 kDa), indicated by the black arrow. **c**, Anti-mucin Western blot using a catalytically inactive biotin-labeled StcE mutant (biotin-StcE^E447D^)^19^ on plasma and tissues from (**b**). Mucin bands are indicated by black arrows. **d**, Mucin Western blot on plasma and tissues 3 hours post I.P. injection of PBS or 10 mg/kg StcE. Mucin bands are denoted by black arrows. Replicate gels stained with IRdye 800CW-streptavidin alone (2° only) are shown.

Bacterial enzymes are currently used as frontline cancer therapeutics; for example, L-asparaginase from *E. coli* is employed in treatment of childhood acute lymphoblastic leukemias^30^. As mucinases had not, to our knowledge, been tested as injectable therapeutics, we assayed StcE for activity and tolerability in vivo. The maximum tolerated dose for StcE treatment in BALB/c and C57BL/6 mice was 0.25 mg per kg (body weight, unless indicated otherwise). Necropsy and complete blood count (CBC) analyses performed 3 h after injection of 15 mg per kg StcE revealed hemorrhages underneath the skull, ecchymoses throughout the gastrointestinal tract, neutrophil accumulation in the lungs and platelet depletion (Extended Data Fig. 2a and Supplemental Table 2). Western blotting using a mucin-specific probe^19^ showed that StcE injected at 0.25 mg per kg remained in circulation for at least 20 h and digested mucins throughout the body, though not as completely as at higher doses (Extended Data Fig. 2b–d). As endothelial and white blood cell surface mucins are critical components of clotting and immune activation pathways^31^, these findings established that an engineered mucinase variant with selectivity for tumor-associated mucins was necessary to avoid on-target, off-tumor effects.

The clinical success of antibody-drug conjugates has shown that fusion of toxic therapeutic cargo to antibodies is a viable strategy for lowering on-target, off-tumor toxicity and increasing on-target efficacy^32^. More recently, antibody-enzyme conjugates have been designed to target the hydrolytic activity of an enzyme to specific subsets of cells^33^. An important design principle of antibody-enzyme conjugates is to ensure that the activity of the enzyme is sufficiently low such that hydrolysis only occurs when the enzyme is concentrated at its target via binding of the antibody. Specifically, in previous work with an antibody of nanomolar affinity, micromolar enzymatic activity was shown to be effective for cell-surface targets^34^. As StcE is active at subnanomolar concentrations, our initial aim was to engineer a mucinase that retained its peptide and glycan specificity but exhibited activity within the micromolar range.

### Structure-guided engineering reduces StcE activity and binding

**Fig. 2 |:**
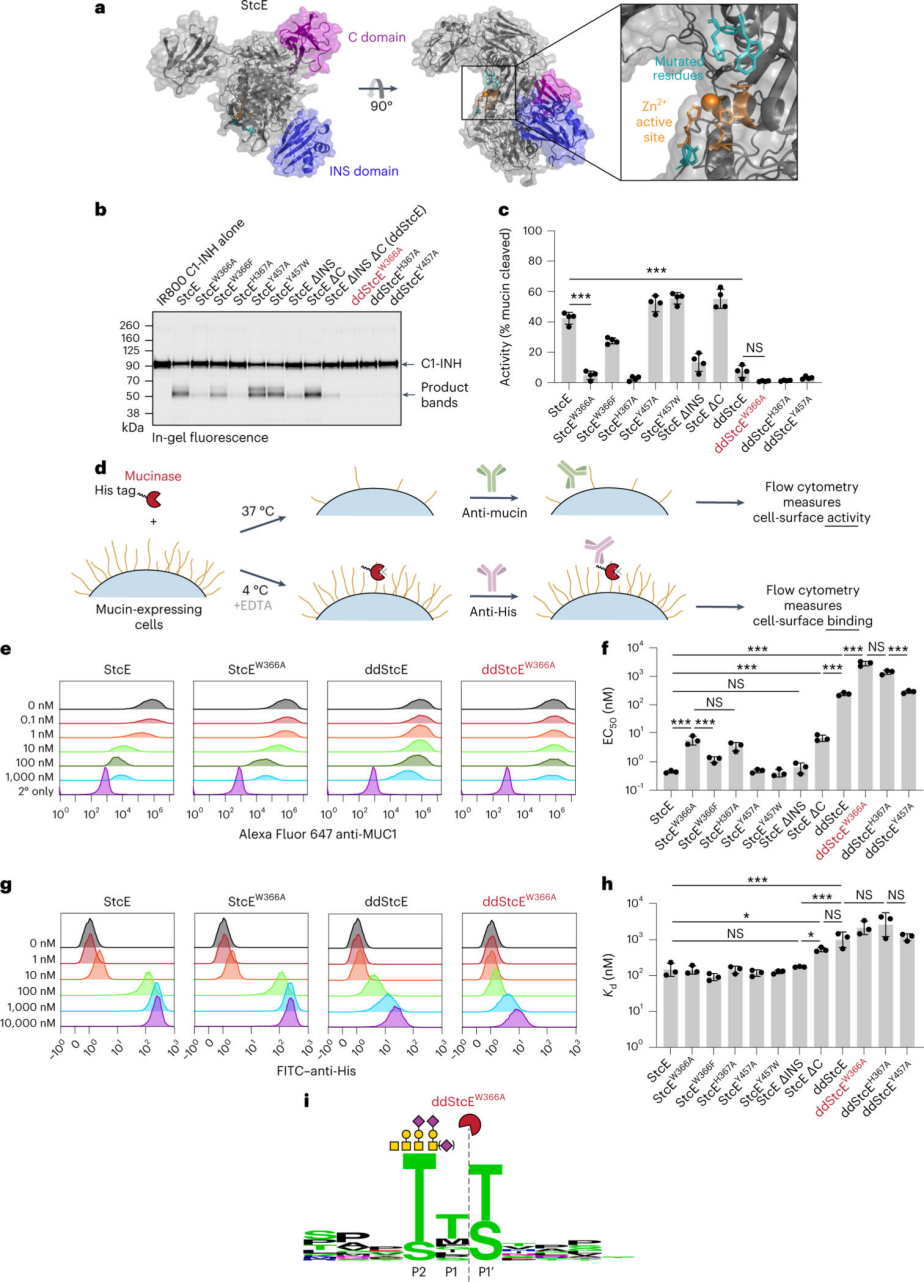
Structure-guided engineering of StcE yields mutants of reduced activity, binding and size. **a**, Structure of StcE, as predicted by ColabFold (Methods)^62^, with the C domain (purple) and INS domain (blue) highlighted. The Zn^2+^ active site is depicted in orange, while mutated residues are shown in teal. **b**, Digestion of IRDye 800CW-labeled purified human mucin C1-INH with 50 nM StcE or StcE mutants, quantified by in-gel fluorescence. **c**, Quantification of **b** (*n* = 4 independent digestions). **d**, Setup for flow cytometry assays measuring cell-surface activity and binding of StcE and StcE mutants. **e**, Representative flow plots showing surface MUC1 levels of HeLa cells treated with StcE mutants at the indicated concentrations. For flow plots of all other StcE mutants, see Supplementary Fig. 1d. **f**, EC_50_ values derived from quantifying mean fluorescence intensity (MFI) from **e** and Supplementary Fig. 1d (*n* = 3 biologically independent replicates). For dose–response curves, see Supplementary Fig. 1e. **g**, Representative flow plots depicting cell-surface binding of StcE variants on HeLa cells measured by anti-His staining. For flow plots of all other StcE mutants, see Supplementary Fig. 1f. **h**, *K*_d_ values derived from quantifying MFI from **g** and Supplementary Fig. 1f (*n* = 3 biologically independent replicates). For dose–response curves, see Supplementary Fig. 1g. **i**, ddStcE^W366A^ cleavage motif as determined by MS on recombinant mucins. Data are shown as mean ± s.d. *P* values were determined using a Tukey-corrected one-way ANOVA; *P < 0.05; ***P < 0.0005; NS, not significant.

We next used the previously published crystal structure^35^ and prior docking studies^18^ to rationally design a StcE mutant with reduced activity and cell-surface binding but retained specificity for mucins. To begin, we deleted two domains, the C and INS domains (Fig. 2a, left), as removal of the INS domain had previously been observed to reduce enzymatic activity, and removal of the C domain had been shown to decrease nonspecific cell-surface binding^35^, both of which were desirable for our targeted mucinase. We also mutated amino acids found near the active site that were not predicted to interact with key substrate residues in the docked complex. Specifically, W366, H367 and Y457 line the active site but do not directly interact with enzyme catalytic residues or substrate P2–P1′ residues, suggesting that mutation of these amino acids might reduce but not abrogate enzymatic activity (Fig. 2a, right, and Supplementary Fig. 1a).

Combinations of domain deletions and single point mutations yielded a total of 11 mutants for characterization, with expression yields ranging from 20 to 100 mg liter^−1^ (Methods and Supplementary Fig. 1b). In vitro activity against recombinant mucin substrates was quantified by densitometry following SDS–PAGE (Fig. 2b,c and Supplementary Fig. 1c). To determine the half-maximal effective concentration (EC_50_) of the mutants for degradation of mucins on live cell surfaces, cells were treated with various concentrations of enzymes for 1 h at 37 °C and subjected to flow cytometry to assess cell-surface MUC1 staining (Fig. 2d–f and Supplementary Fig. 1d,e). To determine effective dissociation constants (*K*_d_) of the mutants, cells were treated with the same concentrations of enzymes for 30 min at 4 °C in the presence of the metal chelator and metalloprotease inhibitor EDTA to prevent enzyme activity. In the latter case, binding was quantified via interaction of an anti-His antibody with the His-tagged mutants (Fig. 2d,g,h and Supplementary Fig. 1f,g).

Combining the two domain deletions (ΔC and ΔINS) yielded double deletion StcE (ddStcE), which exhibited reduced activity and binding along with a molecular weight reduction to 76 kDa relative to the 98-kDa parent enzyme. Nevertheless, the EC_50_ and *K*_d_ of ddStcE on cells remained in the high nanomolar range. Of the single point mutations, W366A and H367A most drastically reduced activity against recombinant and cell-surface mucins (Fig. 2c,f). Adding W366A and H367A mutations to the ddStcE scaffold yielded ddStcE^W366A^ and ddStcE^H367A^, which were active in the desired micromolar range (for discussion of enzymatic activity relative to binder affinity, see above), with EC_50_ values of ~3 and ~1 μM, respectively, and effective *K*_d_ values of ~2 μM each (Fig. 2f,h). ddStcE^W366A^, which we refer to as engineered StcE (eStcE), was selected as the lead candidate for targeting because it exhibited the lowest activity against cell-surface MUC1. We characterized the cleavage motif of eStcE using MS, as was previously done for StcE^18^, revealing that the mutations did not alter substrate recognition (Fig. 2i and Supplemental Table 3).

### A nanobody–eStcE fusion enables targeted mucin degradation

**Extended Data Fig. 3 |:**
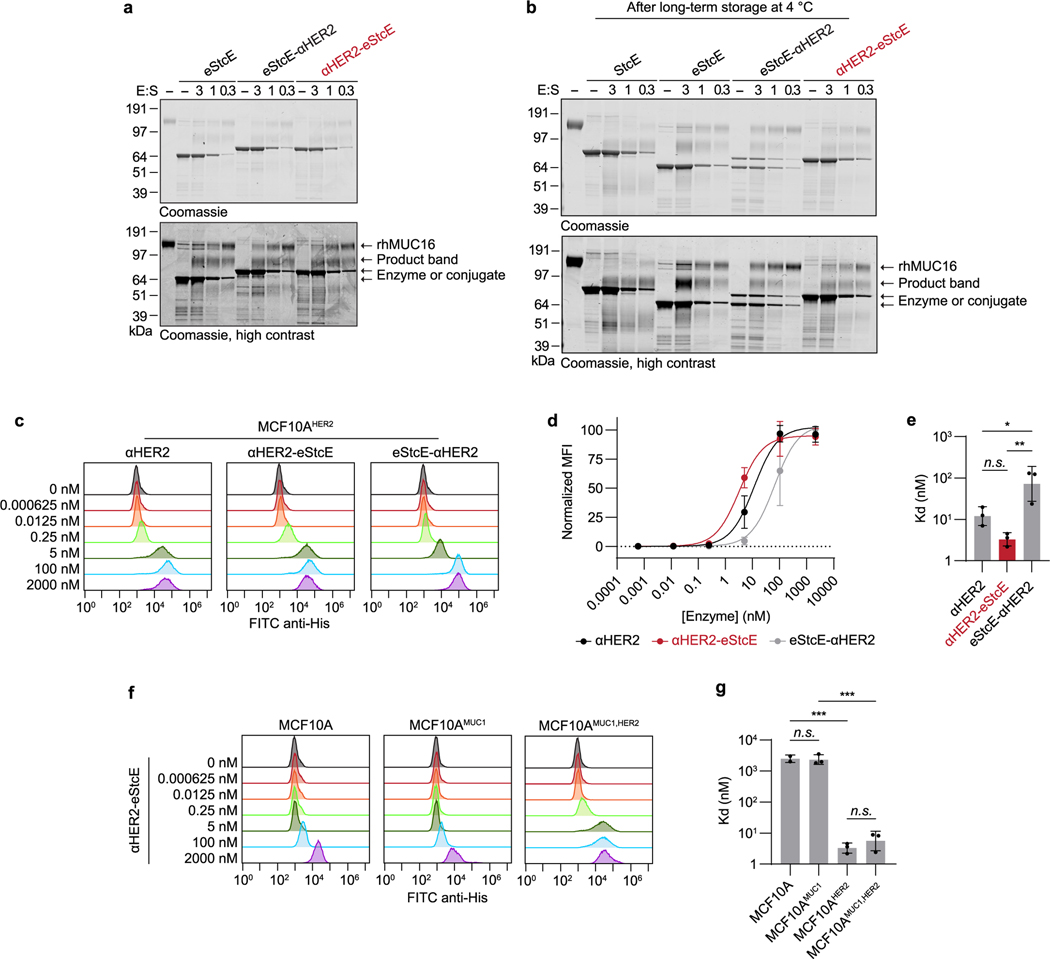
Expression and characterization of engineered nanobody-mucinase conjugates. **a**, Digestion of recombinant human MUC16 (rhMUC16) with eStcE alone or nanobody-eStcE conjugates. **b**, rhMUC16 in-gel digest depicting degradation of eStcE-*αHER2* conjugate after long-term storage at 4 °C. **c**, Representative flow plots showing cell surface binding of nanobody alone and eStcE-*αHER2* on MCF10A^HER2^ cells measured by anti-His staining. **d**, Cell surface binding curves derived from mean fluorescence intensity from (**c**) (*n* = 3 biologically independent replicates). **e**, Kd values derived from (**d**). **f**, Representative flow plots showing cell surface binding of *αHER2*-eStcE on MCF10A^±MUC1, ±HER2^ cells measured by anti-His staining. Flow plot of *αHER2-eStcE on* MCF10A^HER2^ is shown in (**c**). **g**, Kd values derived from mean fluorescence intensity from (**f**) and Fig. 3c (*n* = 3 biologically independent replicates). The MCF10A^HER2^ bar depicts the same data as the *αHER2*-eStcE bar in (**e**). Data are mean ± s.d. *P*-values were determined using Tukey-corrected one-way ANOVA. *p < 0.05, **p < 0.005, ***p < 0.0005.

**Fig. 3 |:**
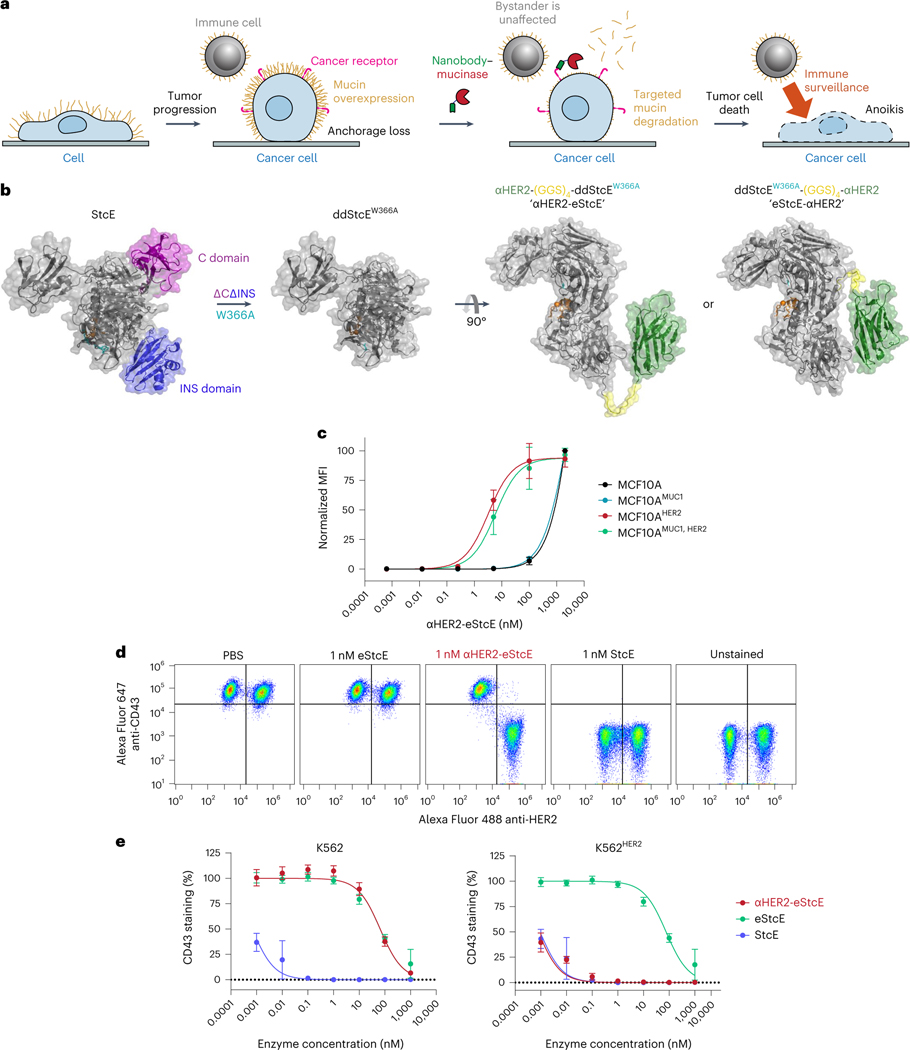
An optimized nanobody-mucinase conjugate selectively cleaves mucins from HER2^+^ cells. **a**, Schematic depicting reversal of mucin-driven tumor-progressive pathways via treatment with a targeted nanobody-mucinase conjugate. **b**, Structure of nanobody-mucinase conjugates, as predicted by ColabFold (Methods)^62^ with the engineering strategy shown. The HER2-targeting nanobody is depicted in green, the active site is shown in orange, the mutated residue (W366A) is in teal, and the flexible linker is shown in yellow. **c**, Binding curves of αHER2-eStcE on MCF10A^±MUC1, ±HER2^ cells quantified by anti-His mean fluorescence intensity (MFI) as in Fig. 2d (*n* = 3 biologically independent replicates). For flow plots and *K*_d_ values, see Extended Data Fig. 3c,f,g. **d**, Representative flow plots depicting surface CD43 levels of mixed K562^±HER2^ cells treated with 1 nM mucinases or conjugate overnight. For representative flow plots with shorter incubation times, see Extended Data Fig. 5e. **e**, CD43 cleavage curves derived from MFI values from **d** (*n* = 3 biologically independent replicates). Data are shown as mean ± s.d.

We envisioned that targeting eStcE to cancer cells would reverse biophysical and immunological tumor-progressive pathways while leaving bystander cells unaffected (Fig. 3a). As immune cells bind Fc-containing biologics through the Fc receptor^36^, we created a genetic fusion to a nanobody rather than an antibody. The cell-surface receptor HER2 was selected as the target antigen because it is upregulated in several carcinoma subtypes, including breast and ovarian cancer, and is bound by a well-validated nanobody, 5F7 (ref. 37). We designed two different fusion orientations that we tested for expression yield, stability, mucinase activity and cell-surface HER2 binding (Fig. 3b). Both orientations of the chimera were expressed in endotoxin-deficient ClearColi at 30–60 mg liter^−1^ and were similarly active against recombinant MUC16 as eStcE alone (Extended Data Fig. 3a). The conjugate with the mucinase C-terminal to the nanobody, which we refer to as ‘αHER2-eStcE’, was stable and retained activity after months at 4 °C, while the other orientation ‘eStcE-αHER2’ exhibited reduced activity following equivalent storage conditions (Extended Data Fig. 3b). Effective dissociation constants (*K*_d_) for nanobody-mucinase conjugates were determined via flow cytometry of HER2^+^ cells as described above, giving values of 11, 4 and 58 nM for αHER2, αHER2-eStcE and eStcE-αHER2, respectively (Extended Data Fig. 3c–e). Therefore, αHER2-eStcE was selected for further in cellulo and in vivo analyses due to its stability and affinity to HER2^+^ cells.

We used cells transduced with a doxycycline-inducible MUC1 ectodomain construct to test the contributions of the nanobody and the enzyme components of αHER2-eStcE to mucin binding. αHER2-eStcE bound to HER2^+^ cell surfaces with a *K*_d_ value approximately three orders of magnitude higher than its binding to HER2^−^ cells, indicating that αHER2-eStcE bound to cells via HER2 affinity and not mucin affinity (Fig. 3c and Extended Data Fig. 3f,g). Indeed, HER2^+^ cells treated with αHER2-eStcE over a 4-h time course exhibited an approximately tenfold decrease in CD43 staining but retained αHER2-eStcE cell-surface residency, indicating that the conjugate does not need to stably bind mucins to deplete cellular mucins (Supplementary Fig. 2).

**Extended Data Fig. 4 |:**
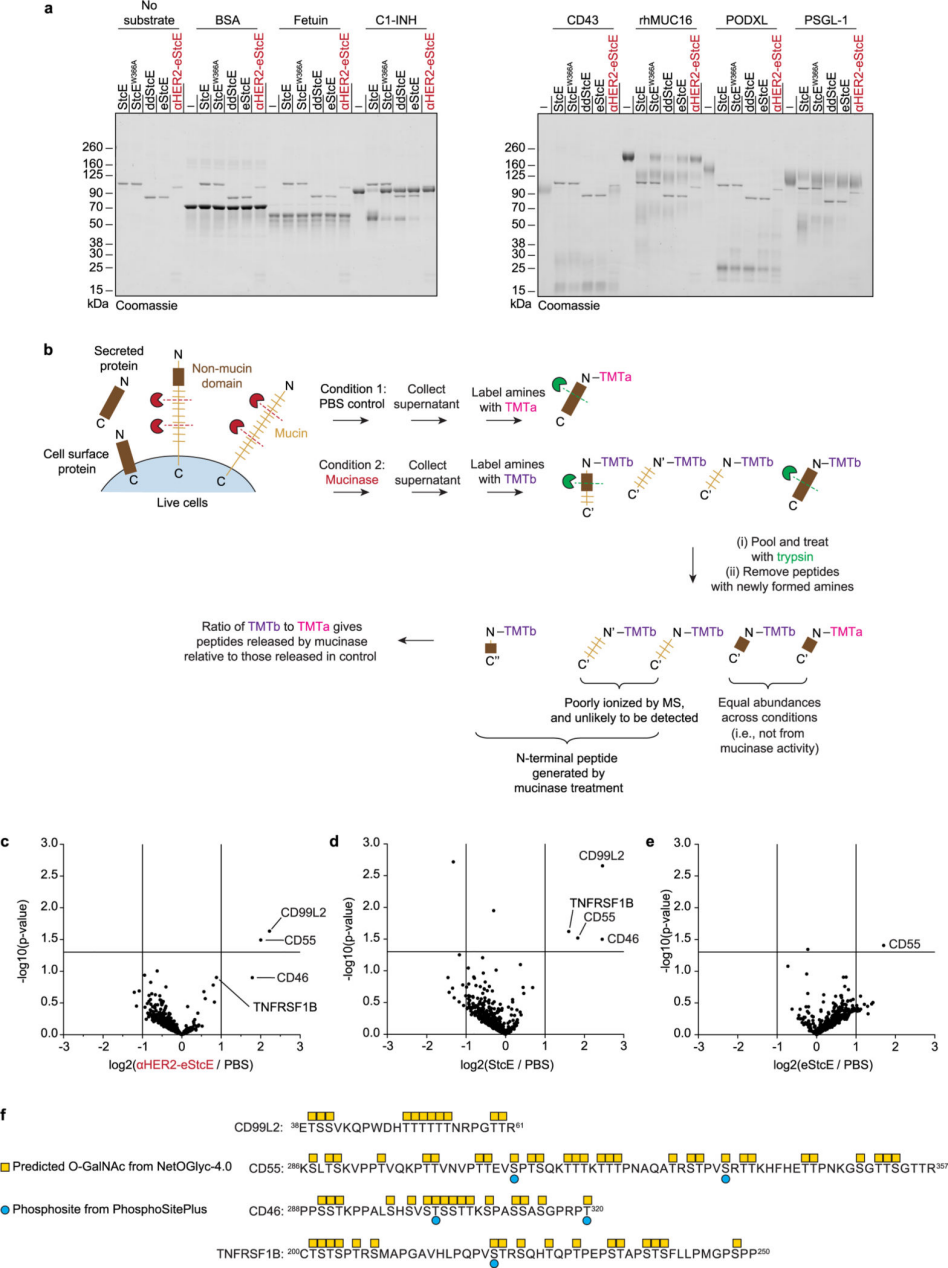
Assessment of αHER2-eStcE selectivity for mucin substrates *in vitro* and on cell surfaces. **a**, Digestion of recombinant or purified non-mucins (BSA, fetuin) and mucins (C1-INH, CD43, PODXL, PSGL-1) with StcE, StcE mutants, and *αHER2*-eStcE. **b**, Setup for terminal amine isotopic labeling of substrates mass spectrometry (TAILS MS) experiment. Mucinase-generated peptides derived from mucin domains were not searched for because of search space complications caused by glycan modifications. **c-e**, Volcano plots depicting enrichment of peptides following treatment of K562^HER2^ cells with *αHER2*-eStcE (**c**), StcE (**d**), or eStcE (**e**) relative to vehicle control (*n* = 4 biologically independent replicates). *P*-values were determined using two-tailed t-tests with multiple testing corrections performed using a permutation-based FDR with 250 randomizations, an FDR of 0.05 and an S0 value of 2. **f**, Annotation of predicted O-glycosites (yellow squares)^16^ and known phosphosites (blue circles)^74^ in putative mucin domains of enriched proteins from (**c-e**).

Cleavage assays with a panel of recombinant non-mucin and mucin proteins confirmed that the fusions maintained mucin selectivity (Extended Data Fig. 4a). To test the specificity of αHER2-eStcE for mucins versus non-mucin proteins on cell surfaces, we used terminal amine isotopic labeling of substrates MS (TAILS MS), which is a method optimized for detection of peptides generated from protease digestion of live cells^38^. A HER2^+^ suspension cell line was treated with vehicle control, StcE, eStcE or αHER2-eStcE, and supernatants were collected and subjected to TAILS MS (Extended Data Fig. 4b). Notably, analysis of peptides generated relative to vehicle control confirmed selectivity for mucin-domain glycoproteins, similar cleavage profiles between StcE and αHER2-eStcE, and reduced activity for untargeted eStcE (Extended Data Fig. 4c–f).

**Extended Data Fig. 5 |:**
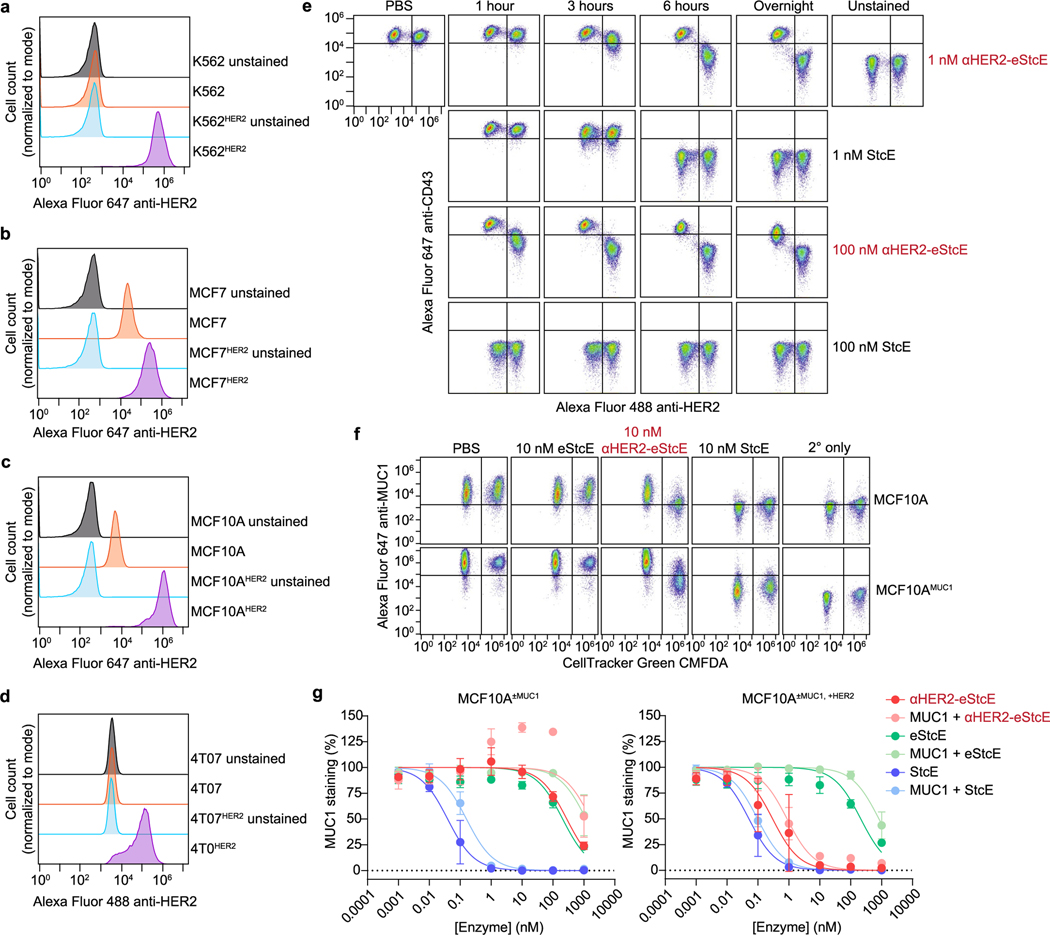
Mixed cell assays to assess targeted de-mucination using generated HER2+ cell lines. **a-d**, Surface HER2 levels of K562^±HER2^ (**a**), MCF7^±HER2^ (**b**), MCF10A^±HER2^ (**c**), and 4T07^±HER2^ (**d**) cells measured by flow cytometry. **e**, Representative flow plots depicting surface CD43 levels of mixed K562^±HER2^ cells treated with StcE or conjugate for the indicated times and concentrations. **f**, Representative flow plots depicting surface MUC1 levels of mixed MCF10A^±MUC1, ±HER2^ cells treated with 10 nM mucinases or conjugate. MCF10A^±MUC1, +HER2^ cells were pre-labeled with CellTracker Green CMFDA. **g**, MUC1 cleavage curves derived from mean fluorescence intensity from (**f**) (*n* = 3 biologically independent replicates). Data are mean ± s.d.

**Extended Data Fig. 6 |:**
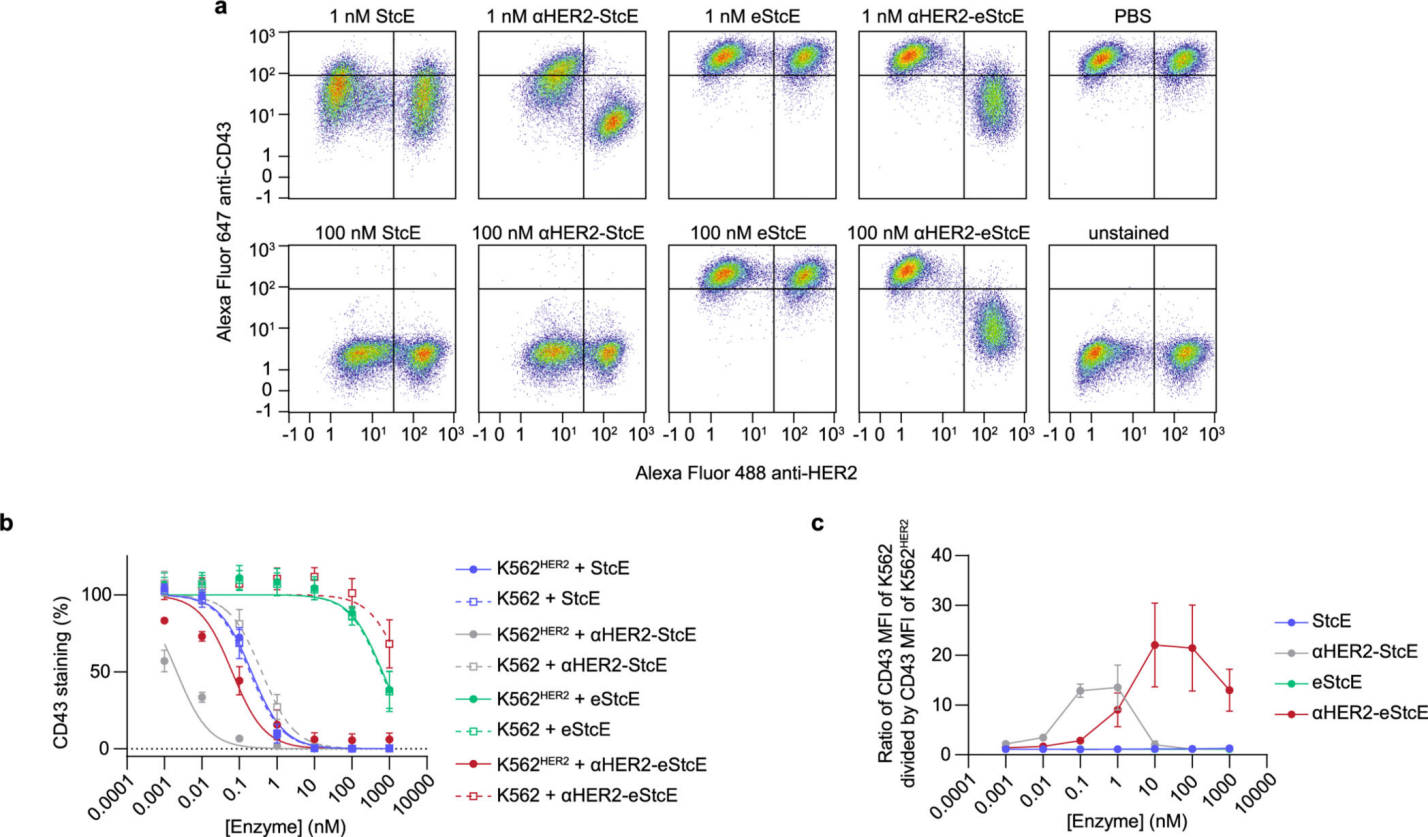
αHER2-eStcE expands the therapeutic window for selective cleavage of mucins from HER2+ cells as compared to αHER2-StcE. **a**, Representative flow plots from three biologically independent replicates depicting surface CD43 levels of mixed K562^±HER2^ cells treated with StcE, *αHER2*-StcE, eStcE, or *αHER2*-eStcE for 1 hour. **b**, CD43 cleavage curves derived from median fluorescence intensity from (**a**) (*n* = 3 biologically independent replicates). **c**, Selective cleavage of mucins on HER2+ cells derived from the ratio of CD43 median fluorescence intensity on K562 divided by CD43 median fluorescence intensity of K562^HER2^ from (**a**) (*n* = 3 biologically independent replicates).Data are mean ± s.d.

Next, we tested the conjugate’s on-target activity through mixed cell assays with various human cancer cell lines engineered to express HER2 (Extended Data Fig. 5a–d). Mixed HER2^+^ and HER2^−^ cells were treated with StcE, eStcE or αHER2-eStcE overnight, and depletion of cell-surface mucins was analyzed via live-cell flow cytometry to quantify mucin staining (anti-CD43). StcE treatment at 1 nM resulted in complete removal of cell-surface mucins on both HER2^+^ and HER2^−^ cells, while 1 nM eStcE resulted in no discernable removal of mucins in either population. By contrast, 1 nM αHER2-eStcE resulted in complete loss of cell-surface mucins on HER2^+^ cells and no discernable loss of mucins on HER2^−^ cells (Fig. 3d,e and Extended Data Fig. 5e). The same trend was observed at higher doses in another cell line interrogated for cell-surface residency of a different mucin protein, MUC1 (Extended Data Fig. 5f,g). A fusion of the parent enzyme StcE with the αHER2 nanobody, termed ‘αHER2-StcE’, was unable to remove mucins solely from HER2^+^ cells at any tested concentration (0.001 to 1,000 nM), confirming the need for engineering of a lower-activity mutant (Extended Data Fig. 6).

**Extended Data Fig. 7 |:**
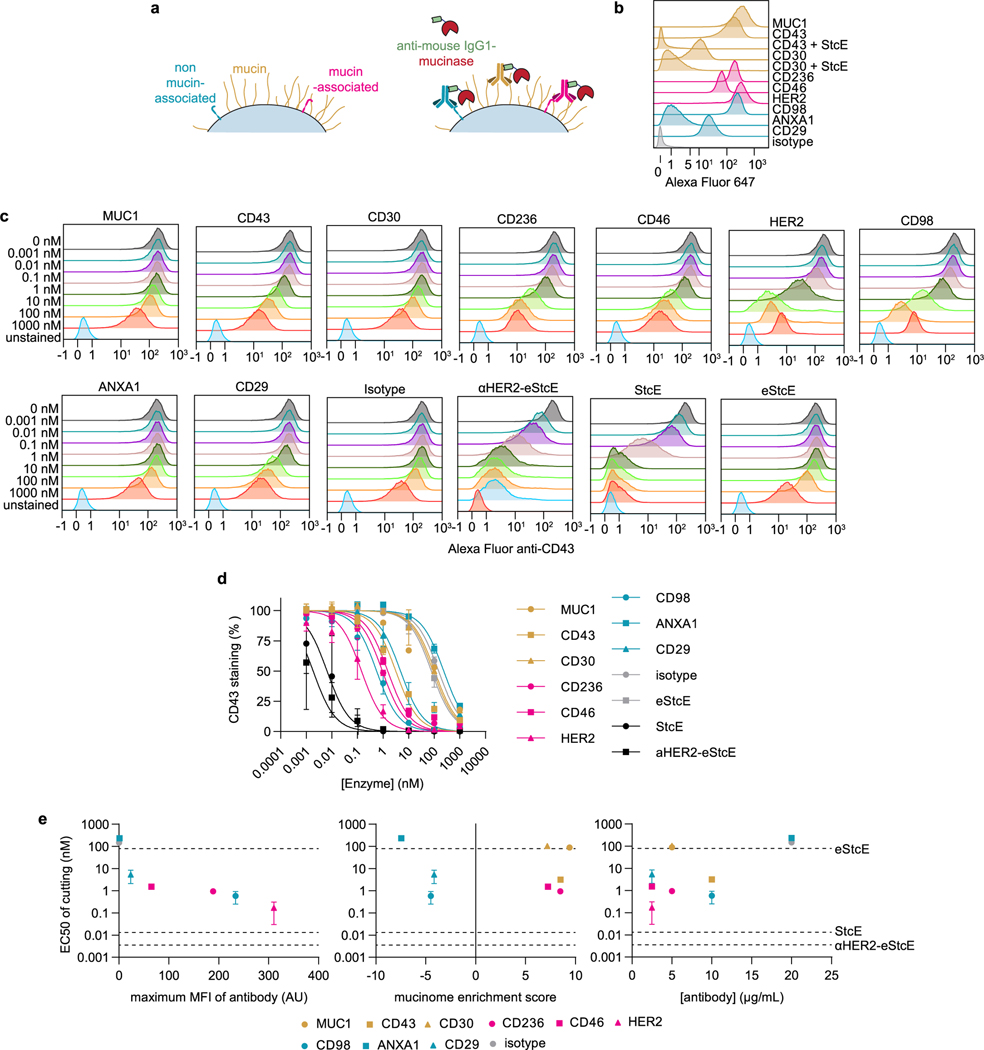
The targeted mucin degradation approach is generalizable for cell surface binding targets. **a**, Schematic depicting targeted mucin degradation using a single anti-mouse IgG1-mucinase (*αIgG1-eStcE)* conjugate and primary mouse IgG1 antibodies against non mucin-associated, mucin, and mucin-associated cell surface epitopes. **b**, Flow cytometry plots showing maximum K562^HER2^ cell surface staining achieved with each primary (1.25–20 μg/mL) and Alexa Fluor 647 anti-mouse IgG1 secondary. To test if anti-mucin antibodies were StcE-sensitive, K562^HER2^ cells were treated with 100 nM StcE for 1 hour. The MUC1 antibody was previously confirmed to be StcE-sensitive in Extended Data Fig. 5f. **c**, Representative flow plots depicting surface CD43 levels of K562^HER2^ cells treated with StcE, eStcE, and *αHER2-eStcE, or* indicated primary antibody and *αIgG1-eStcE for 4 hours*. **d**, CD43 cleavage curves derived from median fluorescence intensity from (**c**) (*n* = 3 biologically independent replicates). **e**, EC50 of CD43 cutting derived from curve fitting (**d**) were compared to the maximum median fluorescence intensity of primary binding in (**b**) (*left*), target’s mucinome enrichment score (Methods) (*center*)^40^, or the concentration of primary used in the cutting experiment (*right*). The dotted lines represent mean EC50 values for eStcE, StcE, and *αHER2-eStcE from (****d****)*. Mucins were not plotted in the left graph since their MFI changes during mucin depletion, and HER2 and isotype were not plotted in the center graph since they do not have a mucinome score. Data are mean ± s.d.

Finally, to address the generalizability of our targeted mucin degradation approach, we fused a previously published and well-validated anti-mouse IgG1 Fc nanobody (TP1107) (ref. 39) to eStcE to generate a conjugate termed ‘αIgG1-eStcE’ and confirmed that it performed comparably to αHER2-eStcE when used in combination with a HER2-targeting antibody (Supplementary Fig. 3). We cotreated cells with αIgG1-eStcE and primary antibodies to mucin, mucin-associated, and non-mucin-associated cell-surface antigens, chosen based on enrichment scores from the recently published cell mucinome (Methods)^40^. The resulting EC_50_ values for mucin depletion were plotted against the concentration of primary antibody used, the target’s mucinome enrichment score and the maximum median fluorescence intensity of primary antibody binding (Extended Data Fig. 7). The maximum median fluorescence intensity of primary antibody binding was the sole factor that correlated with EC_50_ trends, suggesting the absence of any specific requirement for the targeting agent beyond the ability to load enzyme onto cell surfaces.

### αHER2-eStcE selectively kills HER2^+^ cells

**Fig. 4 |:**
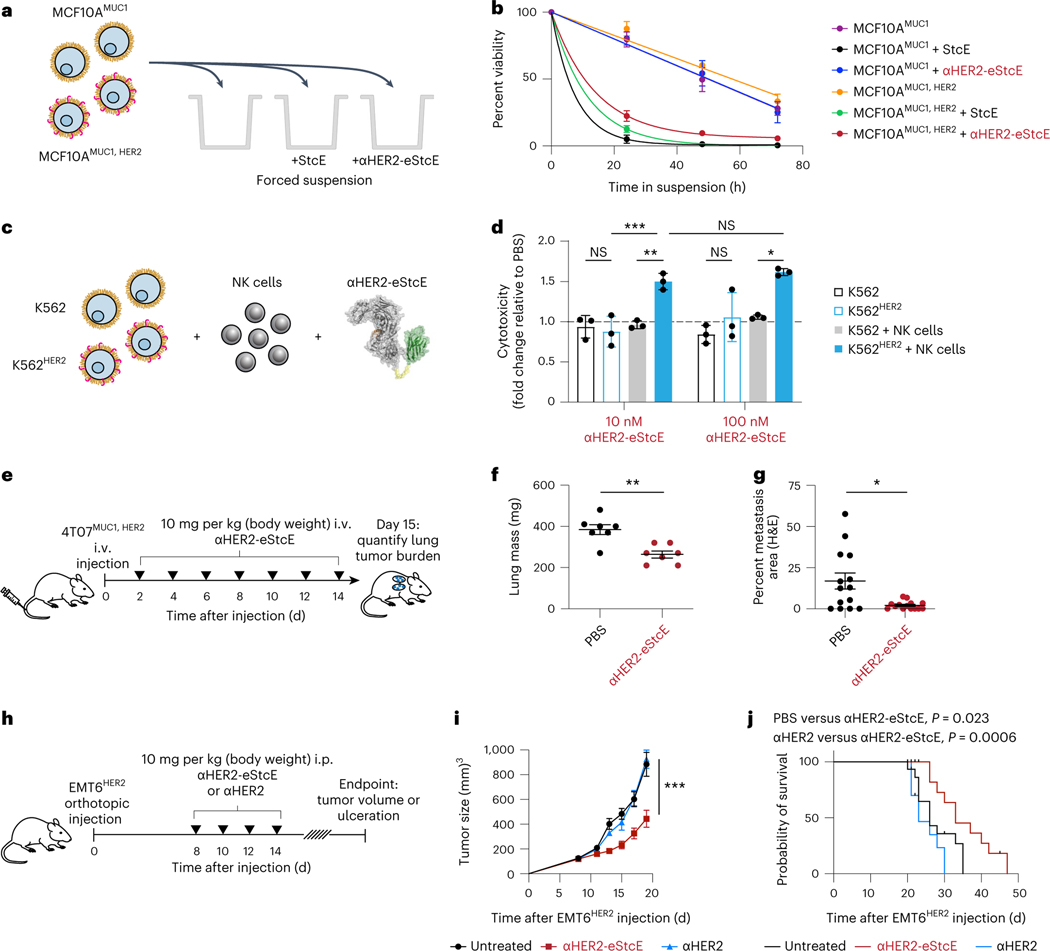
αHER2-eStcE is effective in mixed cell assays and breast cancer mouse models. **a**, Setup for mixed cell suspension survival assays under anchorage-free conditions as in Fig. 1b. **b**, Viability of anchorage-free mixed MCF10A^MUC1, ±HER2^ cells ± 1 nM StcE or αHER2-eStcE over 72 h as determined by flow cytometry (*n* = 3 biologically independent replicates). **c**, Setup for mixed cell NK cell killing assay as in Fig. 1d. **d**, Normalized NK cell killing of mixed K562^±HER2^ cells treated with conjugate at a 2:1 effector:target ratio as determined by flow cytometry (*n* = 3 biologically independent replicates). Values are reported as fold change relative to the average of three PBS-treated control replicates (dotted line). **e**, Treatment regimen for BALB/c mice injected i.v. via tail vein with 4T07^MUC1, HER2^ cells. αHER2-eStcE was injected i.v. every other day at 10 mg per kg starting on day 2 (*n* = 7 animals per group). **f**, Plot depicting lung masses of animals described in **e**. **g**, Percent area of lung metastases quantified by H&E tissue staining of animals described in **e**. Points represent individual scans of lung slices (*n* = 2 per animal). For images, see Extended Data Fig. 9f,g. **h**, Treatment regimen for BALB/c mice injected with EMT6^HER2^ orthotopically into the mammary fat pad. αHER2-eStcE or αHER2 was injected four times i.p. every other day starting on day 8 (*n* = 9 animals per group (αHER2), 10 animals per group (αHER2-eStcE) or 12 animals per group (untreated)). Mice were killed once tumor size reached approximately 1,500 mm^3^ or when mice developed ulcerated tumors. **i**, Average growth curves of EMT6^HER2^ tumors for animals described in **h**. **j**, Survival curves for animals described in **h**. Data are shown as mean ± s.e.m. (**b**, **f**, **g** and **i**) or mean ± s.d. (**d**). *P* values were determined by Tukey-corrected two-way ANOVA (**d** and **i**), two-tailed Mann–Whitney test (**f** and **g**) and Mantel–Cox test (**j**); *P < 0.05; **P < 0.005; ***P < 0.0005.

Using a series of mixed cell assays, we tested whether αHER2-eStcE could selectively reverse mucin-dependent tumor-progressive pathways. We repeated biophysical and immunological assays from Fig. 1 using HER2^+^ and HER2^−^ cell populations, which were mixed before enzymatic treatment (Fig. 4a,c). Under anchorage-free conditions, αHER2-eStcE reversed suspension survival in only the HER2^+^ population, whereas StcE treatment resulted in cell death in both populations (Fig. 4b). Likewise, treatment with αHER2-eStcE selectively enhanced NK cell-mediated killing of the HER2^+^ cell population (Fig. 4d and Supplementary Fig. 4). Recently, it was found that removal of cell-surface mucins via mucinase treatment promotes engagement of *trans*-acting phagocytic receptors in macrophages^41^. Thus, we tested αHER2-eStcE in a mixed cell assay with primary human macrophages and immortalized human breast cancer cells, where we observed enhanced phagocytosis of HER2^+^ cells relative to HER2^−^ cells (Supplementary Fig. 5).

### αHER2-eStcE is nontoxic in mice

**Extended Data Fig. 8 |:**
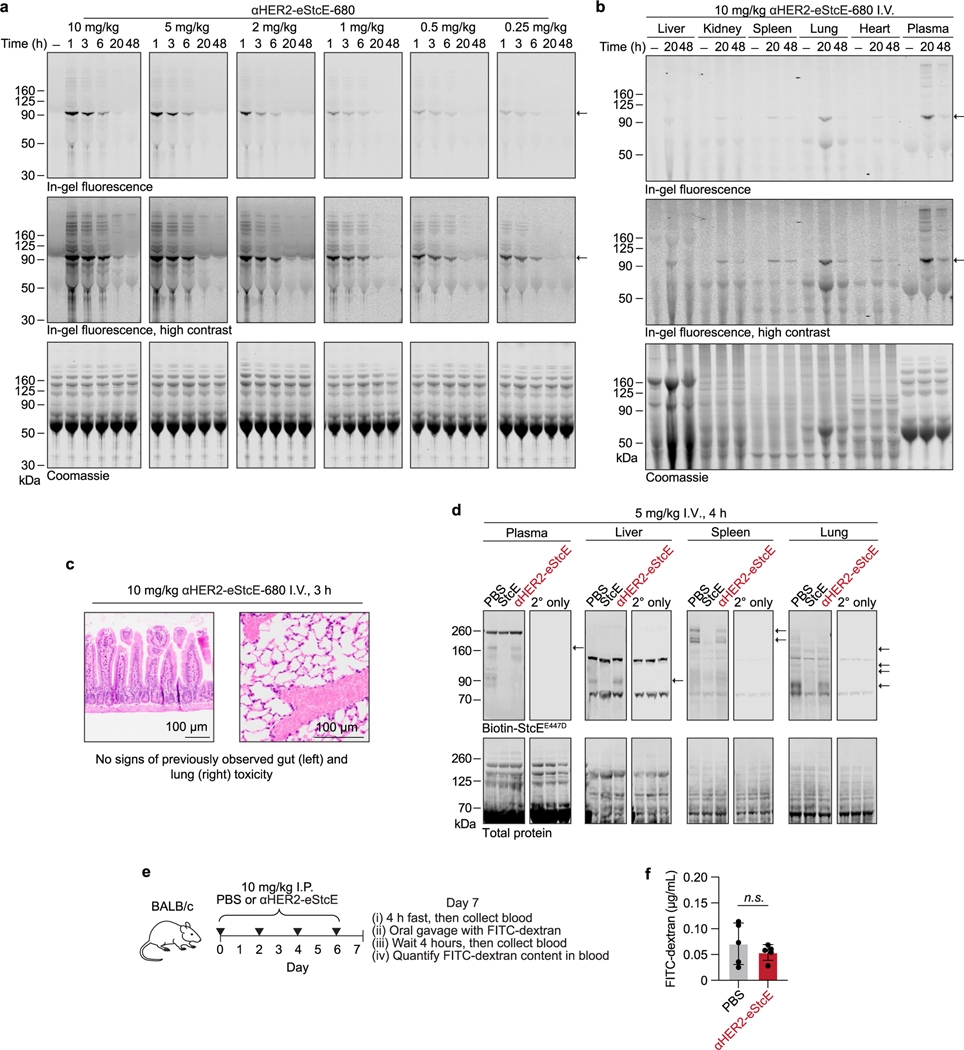
αHER2-eStcE is nontoxic to mice at every tested dose and distributes widely across tissues. **a**, SDS-PAGE of plasma from mice post retro-orbital injection of PBS or IRdye 680RD-labeled *αHER2*-eStcE (*αHER2*-eStcE-680) at the indicated doses. *αHER2*-eStcE is denoted by the black arrow. **b**, SDS-PAGE of plasma and tissues post retro-orbital injection of 10 mg/kg *αHER2*-eStcE-680, denoted by the black arrow. **c**, Necropsy analysis 3 hours post retro-orbital injection of 10 mg/kg *αHER2*-eStcE-680 revealed no abnormalities. **d**, Mucin Western blot on plasma and tissues 4 hours post retro-orbital injection of 5 mg/kg StcE or conjugate. Mucin bands are denoted by black arrows. **e**, Treatment regimen and protocol for FITC-dextran permeability assay. **f**, Concentration of FITC-dextran in plasma of BALB/c mice treated with PBS or αHER2-eStcE (*n* = 5 animals per group). Data are mean ± s.d. *P*-values were determined using two-tailed unpaired t-test.

Intravenous (i.v.) administration of fluorophore-labeled αHER2-eStcE at doses ranging from 0.25 to 10 mg per kg to BALB/c mice revealed that the conjugate remained in blood and tissues for at least 20 h, with no discernable toxicity (Extended Data Fig. 8a,b). Blinded necropsy and CBC analyses confirmed no abnormalities at the highest tested dose of 10 mg per kg (Extended Data Fig. 8c and Supplemental Table 4). To assess mucin depletion in tissues, we injected 5 mg per kg StcE or αHER2-eStcE i.v. into BALB/c mice, collected plasma, liver, spleen and lung 4 h after injection, and immunoblotted for mucins. In all cases, αHER2-eStcE injection resulted in reduced mucin depletion compared to the wild-type parent enzyme (Extended Data Fig. 8d). The integrity of the gastrointestinal mucus layer was also maintained with repeated doses (Extended Data Fig. 8e,f).

### αHER2-eStcE blunts tumor burden and metastasis in mice

To assess on-target efficacy of αHER2-eStcE, we turned to previously validated mouse models of breast cancer progression. The mouse cell line 4T07 is a BALB/c syngeneic mammary carcinoma that efficiently metastasizes to sites such as the lung but is unable to efficiently proliferate at metastatic sites^42^. Woods et al. showed that elaboration of 4T07 cell surfaces via ectopic expression of the MUC1 ectodomain or with lipid-anchored mucin mimetic glycopolymers enhances proliferation in the metastatic niche through PI3K-Akt mechanosignaling pathways related to cell cycle progression^23^. This model involved tail vein injection of luciferase-expressing 4T07 cells into BALB/c mice, where upon cells were lodged in the small capillaries of the lung. At day 15 after injection, animals were killed, and tumor burden in the lung was quantified by lung mass and immunohistochemistry (IHC).

**Extended Data Fig. 9 |:**
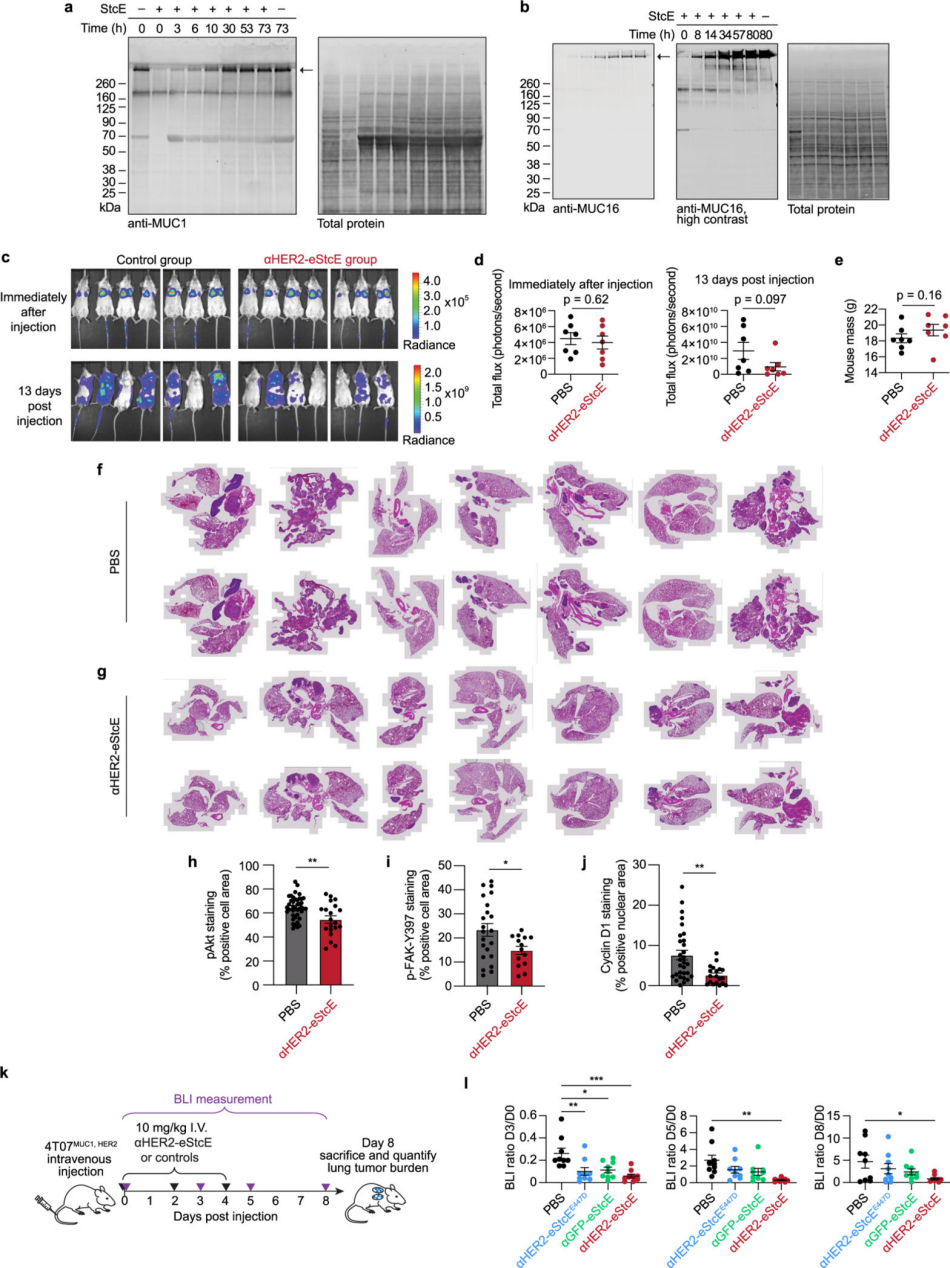
In the 4T07^MUC1, HER2^ murine model of breast cancer progression, *αHER2*-eStcE reduces lung metastatic burden and the prosurvival mechanosignaling markers, pAkt, p-FAK-Y397, and cyclin D1^23^. **a-b**, 4T07^MUC1^ cells (**a**) and OVCAR-3 cells (**b**) were treated with 50 nM StcE for 2 hours, washed 1x with 2 mM EDTA followed by 5x with DPBS, then cultured for the indicated times. Cells were then lysed and subjected to Western blotting for MUC1 (**a**) and MUC16 (**b**). Mucin bands are denoted by black arrows. **c**, Bioluminescent imaging of animals described in Fig. 4e. **d**, Total flux measurements quantified from (**c**). **e**, Plot depicting mouse masses of animals described in Fig. 4e. **f-g**, H&E staining of lungs from PBS-treated (**f**) or *αHER2*-eStcE treated (**g**) animals (*n* = 7 animals per group, 2 slides per animal). Percent area of lung metastases is quantified in Fig. 4g. **h**, Quantification of images from Supplementary Fig. 6 using the IHC profiler plugin in ImageJ. Percent positive corresponds to positive DAB staining in the cytosol. **i**, Quantification of images from Supplementary Fig. 7 using the IHC profiler plugin in ImageJ. Percent positive corresponds to positive DAB staining in the cytosol. **j**, Quantification of images from Supplementary Fig. 8 using the IHC profiler plugin in ImageJ. Percent positive corresponds to positive DAB staining in the nucleus. **k**, Treatment regimen for BALB/c mice injected intravenously (I.V.) via tail vein with 4T07^MUC1, HER2^ cells. Doxycycline was included in the chow for the duration of the experiment to maintain MUC1 ectodomain expression. αHER2-eStcE at 10 mg/kg or an equimolar quantity of αHER2-eStcE^E447D^ or αGFP-eStcE were injected I.V. every other day starting on day 0 (*n* = 9 animals per group). **l**, Total flux of the indicated days normalized to the total flux on day 0 for each mouse quantified from Supplementary Fig. 10. Data are mean ± s.e.m. *P*-values were determined using two-tailed Mann-Whitney test (**d-e**), two-tailed unpaired t-test (**h-j**), or Tukey-corrected one-way ANOVA (**l**). *p < 0.05, **p < 0.005, ***p < 0.0005.

We performed a therapeutic model with 4T07 cells stably expressing the MUC1 ectodomain and HER2 to assess whether αHER2-eStcE would influence metastatic outgrowth to the lung. Treatment was administered i.v. every other day with 10 mg per kg αHER2-eStcE or vehicle control (Fig. 4e). The dosing strategy was chosen based on (1) the observed at least 20-h in vivo circulation time (Extended Data Fig. 8a) and (2) the approximately 24-h turnover observed in cellulo for enzymatically degraded mucins (Extended Data Fig. 9a,b), consistent with reported mucin half-lives^43^. Bioluminescent imaging (BLI) directly following injection confirmed that 4T07^MUC1, HER2^ cells seeded the lungs of both control and treatment group animals (Extended Data Fig. 9c,d). BLI at 13 d after injection revealed reduced tumor burden in αHER2-eStcE-treated animals (*n* = 7, *P* = 0.097). Total mouse mass did not differ significantly between groups (Extended Data Fig. 9e). Necropsy of animals on day 15 after injection showed a decreased wet lung mass (*n* = 7, *P* = 0.0041) and decreased percent metastatic area by hematoxylin and eosin (H&E) tissue staining (*n* = 7 mice and two lung sections per mouse; *P* = 0.0061; Fig. 4f,g and Extended Data Fig. 9f,g). IHC analysis of lungs revealed reduced staining of phosphorylated Akt, phosphorylated FAK-Y397, and cyclin D1 in αHER2-eStcE-treated versus control animals (Extended Data Fig. 9h–j and Supplementary Fig. 6–8). These data indicate that treatment with αHER2-eStcE may blunt metastatic outgrowth in the 4T07 model through reversal of mucin ectodomain-driven enhancement of cell cycle progression via the PI3K-Akt axis^23^. In a separate experiment in which MUC1 ectodomain expression was maintained through constant administration of doxycycline, we tested the efficacy of αHER2-eStcE compared to a non-targeting control, where eStcE was fused to a green fluorescent protein (GFP)-targeting nanobody (3OGO (ref. 44); termed ‘αGFP-eStcE’), and an inactive control with an inactivating mutation to a catalytic residue (termed ‘αHER2-eStcE^E447D^’) (Supplementary Fig. 9)^35^. BLI indicated that while nontargeting and fully inactive controls may initially impair tumor cell survival or enhance tumor cell clearance from the lungs, only targeted αHER2-eStcE treatment produces durable efficacy (Extended Fig. 9k,l and Supplementary Fig. 10).

The mouse cell line EMT6 is a BALB/c syngeneic mammary carcinoma that is used as a model for immune surveillance^45^. Gray et al. showed that desialylation of orthotopic EMT6 tumors with injected sialidase constructs prolonged the survival of mice through inhibition of the Siglec-sialic acid immune checkpoint axis^34^. This model involved injection of EMT6 cells stably expressing HER2 (EMT6^HER2^) into the mammary fat pads of mice followed by intraperitoneal (i.p.) treatment with enzymes or controls.

**Extended Data Fig. 10 |:**
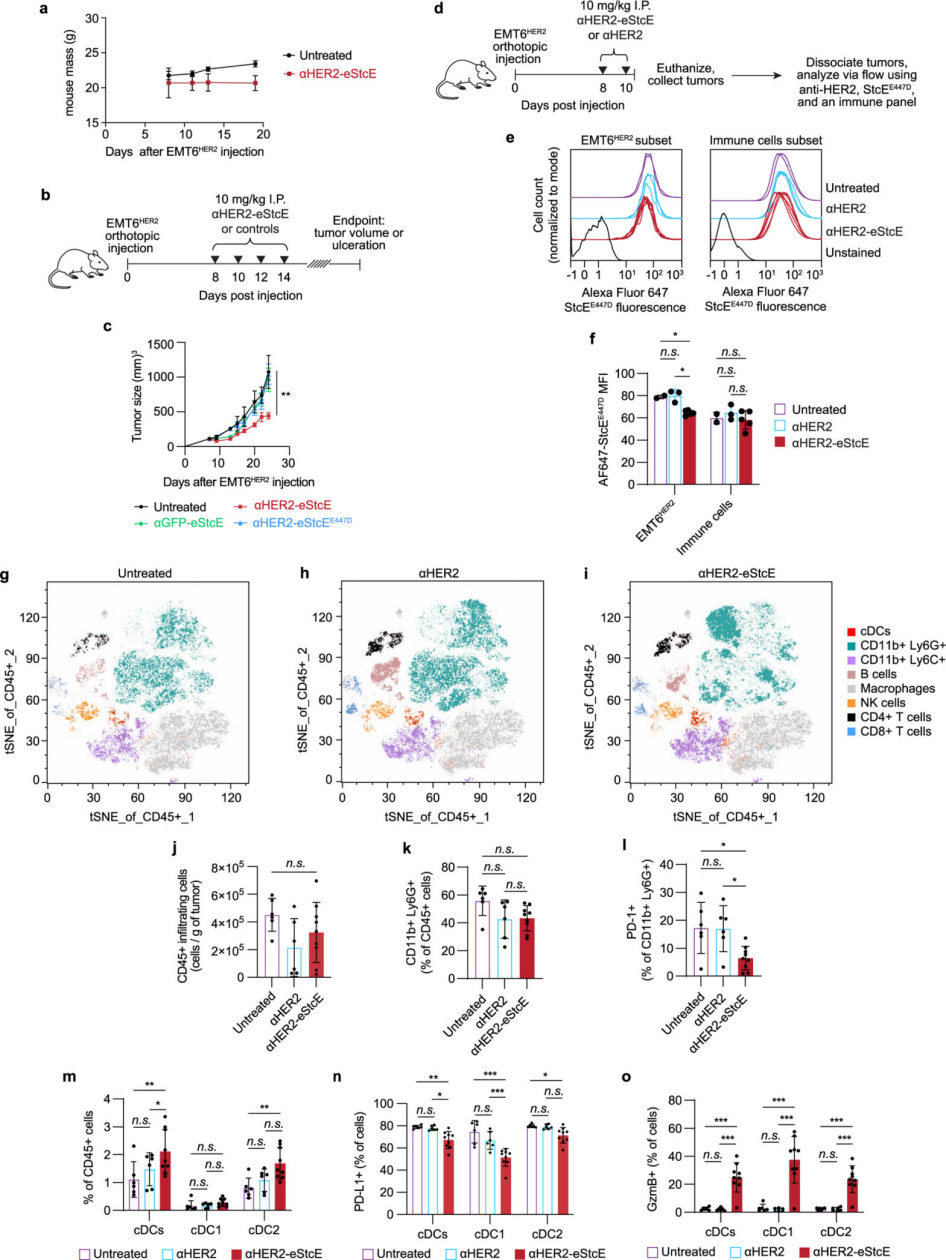
In the EMT6^HER2^ murine model of breast cancer progression, αHER2-eStcE reduces mucin levels on EMT6^HER2^ cells but not immune cells and alters the tumor immune microenvironment. **a**, Plot depicting mouse masses of animals described in Fig. 4h. Mouse masses for αHER2 treated mice were not measured. **b**, Treatment regimen for BALB/c mice injected with EMT6^HER2^ orthotopically into the mammary fat pad. αHER2-eStcE, αHER2-eStcE^E447D^, or αGFP-eStcE were injected four times intraperitoneally (I.P.) every other day starting on day 8 (*n* = 3 animals per group (Untreated, αHER2-eStcE), 5 animals per group (αGFP-eStcE), or 6 animals per group (αHER2-eStcE^E447D^)). The dose was 10 mg/kg for αHER2-eStcE or an equimolar quantity (2.8 nmol) of αHER2-eStcE^E447D^ or αGFP-eStcE. **c**, Average growth curves of EMT6^HER2^ tumors for animals described in (**b**). Mice were euthanized once tumor size reached approximately 1500 mm^3^ or when mice developed ulcerated tumors. **d**, Short-term treatment regimen for BALB/c mice injected with EMT6^HER2^ orthotopically into the mammary fat pad. αHER2-eStcE or αHER2 were injected twice intraperitoneally (I.P.) every other day starting on day 8 (*n* = 6 animals per group (Untreated, αHER2) or 9 animals per group (αHER2-eStcE)). The dose was 10 mg/kg for αHER2-eStcE and an equimolar quantity (2.8 nmol) of αHER2. **e**, Total cell surface mucin staining of CD45^−^/HER2^+^ (EMT6^HER2^ subset) and CD45^+^/HER2^−^ (immune subset) cells isolated from animals described in (**d**) using Alexa Fluor 647-labeled StcE^E447D^ (AF647-StcE^E447D^)^19^. Subsets were defined as shown in Supplementary Fig. 11. **f**, Mean fluorescence intensity (MFI) values derived from (**e**) (*n* = 2, 3, or 5 biologically independent replicates (Untreated, αHER2, and αHER2-eStcE, respectively). **g-i**, T-statistical stochastic neighbor embedding (tSNE) plots depicting immune cell subsets of tumor-infiltrating lymphocytes from untreated (**g**), αHER2 treated (**h**), and αHER2-eStcE treated (**i**) animals described in (**d**). Immune subsets were defined as shown in Supplementary Fig. 12. **j**, Live single CD45+ cells per gram of tumor from animals described in (**d**). **k**, Percent of tumor-infiltrating Ly6G+ cells as a fraction of total CD45+ cells from animals described in (**d**). **l**, Percent of PD-1+ cells in the Ly6G+ cell population from animals described in (**d**). **m**, Percent of tumor-infiltrating cDCs, cDC Type 1 (cDC1s), and cDC Type 2 (cDC2s) as a fraction of total CD45+ cells from animals described in (**d**). **n-o**, Percent of PD-L1+ (**n**) and GzmB+ (**o**) cells in cDC, cDC1, and cDC2 populations, from animals described in (**d**). Data are mean ± s.e.m (**a-b**) or mean ± s.d (**f, j-o)**. *P*-values were determined using Tukey-corrected one-way (**j-l**) and two-way ANOVA (**a, c, f, m-o**). *p < 0.05, **p < 0.005, ***p < 0.0005.

To assess whether mucinase-driven depletion of Siglec ligands from tumor cell surfaces would have a similar beneficial effect, we performed a therapeutic model with EMT6^HER2^ cells and treated animals with four doses of 10 mg per kg αHER2-eStcE, an equimolar dose of αHER2 or vehicle control (Fig. 4h). Treatment with αHER2-eStcE resulted in reduced tumor size at day 19 (*n* = 6–9, *P* < 0.0001; Fig. 4i). After 35 d, all mice in the vehicle-treated group had reached a tumor burden requiring euthanasia, while treatment with αHER2-eStcE extended mouse survival to 47 d (*P* = 0.023 versus control and *P* = 0.0006 versus αHER2 alone; Fig. 4j). Treatment with αHER2 alone did not result in attenuation of tumor growth or prolonged survival, and mice treated with αHER2-eStcE did not exhibit weight loss over the course of the experiment, suggesting that treatment was well tolerated (Extended Data Fig. 10a). In a replicate experiment using a separate set of animals, tumor growth was once again attenuated with treatment of αHER2-eStcE but not with treatment of αHER2-eStcE^E447D^ or αGFP-eStcE, indicating that enzymatic activity and tumor targeting were required for the observed efficacy (Extended Data Fig. 10b,c).

For analysis of mucin degradation and immune infiltration within EMT6^HER2^ tumors, a separate set of animals was treated as described above with vehicle, αHER2, or αHER2-eStcE and euthanized at day 10 after implantation (Extended Data Fig. 10d). Flow cytometry analysis of αHER2-eStcE-treated animals revealed a modest but significant reduction of cell-surface mucins on EMT6^HER2^ cancer cells (CD45^−^HER2^+^ cells) without affecting mucin levels on immune cells (CD45^+^HER2^−^ cells), suggesting that αHER2-eStcE promotes selective mucin depletion in vivo (Extended Data Fig. 10e,fand Supplementary Fig. 11). EMT6^HER2^ cells and immune cells in animals treated with vehicle or αHER2 control did not exhibit alterations in cell-surface mucin levels.

We next profiled the immune composition within EMT6^HER2^ tumors and found that the dominant immune cell type within these tumors was Ly6G^+^ cells, which correspond to Ly6G-expressing granulocytes and/or neutrophils that are often found in breast tumor immune infiltrates (Extended Data Fig. 10g–j and Supplementary Fig. 12)^46^. Tumor-infiltrating Ly6G^+^ cells from mice treated with αHER2-eStcE showed reduced levels of the inhibitory immune checkpoint protein PD-1 relative to vehicle and αHER2 treatment groups (Extended Data Fig. 10k,l). In addition, αHER2-eStcE therapy promoted infiltration of conventional dendritic cells (cDCs) into tumors (Extended Data Fig. 10m). We found that cDCs in αHER2-eStcE-treated tumors exhibited an augmented phenotype, as indicated by reduced levels of the inhibitory ligand PD-L1 (Extended Data Fig. 10n). Strikingly, cDCs of conjugate-treated animals also exhibited significantly increased levels of granzyme B (GzmB), a cytotoxic protease that is released by immune cells to trigger apoptosis of target cells (Extended Data Fig. 10o). While GzmB is typically associated with cytotoxic cells, such as CD8^+^ T cells and NK cells, it can also be produced by other cell types after activation^47^. These data, which support modulation of the tumor immune microenvironment following αHER2-eStcE treatment, are consistent with the importance of mucin and sialic acid signaling in the tumor microenvironment^48–50^.

In summary, targeted degradation of cancer-associated cell-surface mucins limited primary tumor growth and metastasis in mouse models of breast cancer progression, with indications that both biophysical and immunological signaling were influenced.

## Discussion

Over 80% of cell-surface and secreted proteins are predicted to be glycosylated^16^, and specific alterations in protein glycosylation have been observed in diverse pathologies, including inflammation and cancer^4^. An emerging theme from research in this field is that the biological functions of these altered glycoproteins are determined by both their glycan and protein components. Accordingly, disease-relevant protein glycoforms are an important class of therapeutic targets^4^. This observation motivated us to consider new therapeutic modalities that target composite epitopes comprising both protein and glycan. Mucin glycoproteins are attractive targets because their structures and biological activities are equally dependent on their protein and glycan constituents. Further, we and others have characterized bacterial proteases with peptide- and glycan-dependent cleavage motifs that render them highly selective for densely *O*-glycosylated mucin domains^17–21^, suggesting that disease-driving mucins could be selectively degraded by restricting the activity of these mucinases to target cells.

Antibody-enzyme conjugates have been previously used to spatially restrict enzymatic activity in therapeutic contexts. In one technique, termed antibody-directed enzyme prodrug therapy, the enzyme activates a prodrug, resulting in selective release of active compound at a tumor or pathogen site^51^. In another strategy, the enzyme is hydrolytic, resulting in degradation of the antigen or cell death in the vicinity of the affinity reagent’s binding site^52^. Building on this prior work, we recently reported antibody-sialidase conjugates for selective desialylation of tumor cell surfaces, which have entered phase I/II clinical trials (trial identifier NCT05259696) (refs. 33,34).

To design antibody-mucinase conjugates with high on-target selectivity, we drew from insights gained through efforts to engineer so-called molecular glues, which are small molecules that bridge a target protein with an endogenous enzyme^53,54^. Molecular glues, such as proteolysis targeting chimeras, operate through the principle of effective molarity, wherein two reactants that are in close proximity interact pseudointramolecularly, dramatically increasing the reaction rate^55^. Selectivity against a target pool is achieved when two criteria are met: (1) binding of the enzyme conjugate to its substrates (measured, for example, by *K*_d_) is driven by the affinity of the targeting moiety and not the enzyme’s intrinsic affinity, and (2) the enzyme’s activity (measured, for example, by EC_50_) is such that intermolecular reactions occur slower than pseudointramolecular reactions. Based on prior work with antibody-sialidase conjugates, we reasoned that both criteria could be met with a high-affinity antibody and a low-efficiency enzyme^34^.

A series of domain deletions and point mutations yielded an engineered variant of the pan-mucinase StcE^18^, which we term eStcE, exhibiting micromolar *K*_d_ and EC_50_ values on cell surfaces. We fused eStcE to αHER2, which binds the human HER2 receptor with nanomolar affinity^37^. We expected the lack of an immune-cell-binding Fc domain on αHER2 to be an advantage, given widespread expression of functionally important mucins such as CD45 and MUC1 on immune cell surfaces^31^. The conjugate, termed αHER2-eStcE, exhibited high on-target activity as quantified by mixed cell assays in a variety of cell types expressing different mucin proteins. Furthermore, cotreatment with αIgG1-eStcE and a variety of primary antibodies demonstrated the platformability of effective molarity-driven cell-type-selective targeting. For example, in addition to therapeutic applications, we expect that these tools will facilitate functional evaluation of mucins expressed by individual cell types in complex environments.

In cell lines, functional mixed cell assays replicated the biophysical and immunological effects of treatment with the wild-type StcE enzyme and showed high on-target selectivity. In a mouse model of mechanical signaling during lung metastatic outgrowth, αHER2-eStcE treatment decreased lung burden in mucin-overexpressing tumors^23^. In an orthotopic mouse model of breast cancer, previously shown to be driven by sialic acid-mediated immunological silencing^34^, αHER2-eStcE treatment decreased tumor burden and increased survival.

Future work will focus on determining the precise therapeutic contexts in which targeted mucin degradation would be most effective. Based on our data, tumors that are immune infiltrated, susceptible to mechanical stress and sensitive to ferroptosis may be a starting point. Cancers that present as so-called ‘mucinous’ subtypes, wherein individual cancer cells are suspended in a secreted matrix of mucin and polysaccharides, may be a second set of indications^7^. In addition, the range of human diseases that are characterized by aberrant mucin phenotypes, such as respiratory viral infections^56,57^, cystic fibrosis^58^, bacterial endocarditis^59^ and gut dysbiosis^60^, are a third class of disease indications. Further experiments in preclinical models will be necessary to assess whether nanobody-mucinase conjugates have the potential to join other bacterially derived enzymes, such as asparaginase^30^, as frontline therapeutics. Finally, identification of human proteases capable of degrading mucin domains would be beneficial to clinical translation of this class of biologics^61^.

### Online content

Any methods, additional references, Nature Portfolio reporting summaries, source data, extended data, supplementary information, acknowledgements, peer review information; details of author contributions and competing interests; and statements of data and code availability are available at https://doi.org/10.1038/s41587-023-01840-6.

## Methods

### Statistics and reproducibility

For all immortalized cell line data, biological replicates refer to experiments performed independently on different days. All statistical methods were performed using Prism (GraphPad). When multiple conditions were compared, a Tukey-corrected one-way ANOVA was used. In all circumstances in which multiple groups and conditions were compared, a Tukey-corrected two-way ANOVA was used. For EC_50_ and *K*_d_ comparisons, data were log normalized before statistical comparisons. When two groups were compared, a two-tailed unpaired *t*-test or multiple unpaired two-tailed *t*-tests with two-stage Benjamini, Kreiger and Yekutieli false discovery rate correction were used. For mouse survival data, a Mantel–Cox test was used. All gel/blot images are representative of at least *n* = 2 independent replicates.

### Cell culture

Cells were maintained at 37 °C and 5% CO_2_. MCF10A^±MUC1, ±HER2^ cells were cultured in phenol red-free 1:1 DMEM:F12 supplemented with 5% New Zealand horse serum (Thermo Fisher Scientific), 20 ng ml^−1^ epidermal growth factor (Peprotech), 0.5 μg ml^−1^ hydrocortisone (Millipore Sigma), 100 ng ml^−1^ cholera toxin (Millipore Sigma), 10 μg ml^−1^ insulin (Millipore Sigma) and 1% penicillin/streptomycin (P/S). K562^±HER2^, CCRF-CEM and 4TO7^MUC1, HER2^ cells were cultured in RPMI supplemented with 10% heat-inactivated fetal bovine serum (FBS; Thermo Fisher Scientific) and 1% P/S. HeLa, CCRF-HSB-2, EMT6^HER2^ and HEK-293T cells were grown in DMEM supplemented with 10% heat-inactivated FBS and 1% P/S. MCF7 cells were grown in DMEM supplemented with 10% heat-inactivated FBS, 10 μg ml^−1^ human insulin (Thermo Fisher Scientific) and 1% P/S. OVCAR-3^N^ cells were cultured in RPMI supplemented with 10% heat-inactivated FBS, 0.01 mg ml^−1^ bovine insulin (Sigma-Aldrich) and 1% P/S. Cells were counted using a Countess II FL automated cell counter (Thermo Fisher Scientific) following the manufacturer’s recommendations.

### MCF10A^MUC1^ suspension survival assay

MCF10A cells expressing a cytoplasmic truncation of MUC1 (MUC1ΔCT; also referred to as MUC1 ectodomain) were used to limit any possible cytoplasmic signaling^23^. MUC1ΔCT was induced with 200 ng ml^−1^ doxycycline for 24 h. Uninduced and induced cells were seeded at 3 × 10^5^ cells per well in a 24-well ultralow-attachment plate (Corning) in 0.75 ml of complete medium; 200 ng ml^−1^ doxycycline and 10 nM StcE were added as appropriate. The plate was incubated at 37 °C, 5% CO_2_ and 125 r.p.m. At *t* = 0, 24, 48 and 72 h, cells were centrifuged at 350*g* for 5 min, resuspended in 200 μl of PBS with 0.1% benzonase (Sigma-Aldrich) and incubated at room temperature for 15 min. Cells were then resuspended in 200 μl of enzyme-free cell dissociation buffer (Thermo Fisher Scientific) and stained with 100 nM Calcein AM (Thermo Fisher Scientific) and 5 nM Sytox Red dead cell stain (Thermo Fisher Scientific) for 20 min at 4 °C before analysis using a BD Accuri C6 Plus. Videos of MCF10A^MUC1^ cells freshly seeded on standard tissue culture plates (Corning) and treated with and without 1 nM StcE were generated with images taken at 30-min intervals for 18 h using an IncuCyte. The IncuCyte was set to 37 °C and 5% CO_2_, and cells were incubated in complete medium with 200 ng ml^−1^ doxycycline.

For mixed cell assays, MUC1ΔCT was induced with 200 ng ml^−1^ doxycycline for 24 h. A 1:1 mixture of 2.5 × 10^5^ MCF10A^MUC1^ and MCF10A^MUC1, HER2^ cells was seeded per well in a 24-well ultralow-attachment plate in 0.80 ml of complete medium; 200 ng ml^−1^ doxycycline, 1 nM StcE and 1 nM αHER2-eStcE were added as appropriate. The plate was incubated at 37 °C, 5% CO_2_ and 125 r.p.m. At *t* = 0, 24, 48 and 72 h, cells were centrifuged at 350*g* for 5 min, resuspended in 100 μl of PBS with 0.1% benzonase and incubated at room temperature for 15 min. Cells were then resuspended in 200 μl of enzyme-free cell dissociation buffer and stained with Alexa Fluor 647 anti-human CD340 (erbB2/HER2, 24D2 clone; BioLegend) and 500 nM Sytox Green nucleic acid stain (Thermo Fisher Scientific) for 20 min at 4 °C, according to the manufacturer’s recommendations, before analysis using a BD Accuri C6 Plus. All flow cytometry data were analyzed using FlowJo v. 10.0 (TreeStar).

### CD43 and Siglec-7-Fc flow cytometry

K562, CCRF-CEM or CCRF-HSB-2 cells (1 × 10^6^) growing in log phase were collected, resuspended in 1 ml of serum-free RPMI and treated with either vehicle or 50 nM StcE for 1 h. Cells were subsequently centrifuged at 600*g* and washed twice in PBS. Cells were then resuspended in fluorescence-activated cell sorting (FACS) buffer (0.5% bovine serum albumin (BSA) in PBS) at 1 × 10^6^ cells per ml and aliquoted into a V-bottom 96-well plate (Corning) at 1 × 10^5^ cells per well. For staining, a precomplex solution of 1 μg ml^−1^ Siglec-7-Fc (R&D Systems) and 1 μg ml^−1^ Alexa Fluor 488 anti-hFc was made in FACS buffer and incubated on ice for 1 h. Alexa Fluor 647 anti-CD43/sialophorin (MEM-59 clone; Novus Biologicals) was subsequently added to the precomplex solution before staining. Cells were stained in 100 μl of staining solution for 30 min, washed twice with FACS buffer and analyzed by flow cytometry using a BD Accuri C6 Plus.

### Human donor-derived macrophage isolation

Peripheral blood mononuclear cells (PBMCs) were isolated from LRS chambers (Stanford Blood Center) using a Ficoll-Paque density gradient (Cytiva). Isolated PBMCs were extracted from the PBS/Ficoll interface and washed three times with PBS. PBMCs were resuspended in RPMI containing 10% heat-inactivated FBS and plated at 1 × 10^7^ cells per well into a 24-well, number 1.5 glass plate (Cellvis) that was precoated with poly-L-lysine solution (Millipore Sigma). PBMCs were incubated for 1 h at 37 °C to allow monocytes to adhere to the glass. Cells were then rinsed three times with PBS to remove contaminating lymphocytes. Medium was replaced with IMDM containing 10% human AB serum (Gemini). Monocytes were differentiated for 7–9 d.

### NK cell isolation

PBMC aliquots were quickly thawed and diluted in 10 ml of RPMI containing DNase to break up cell aggregates. Cells were incubated at 5% CO_2_ and 37 °C for 30 min and subsequently counted in duplicate. Cells were then centrifuged at 600*g* and resuspended in RPMI to a final cell concentration of 50 × 10^6^ cells per ml. Isolation of NK cells was performed according to the manufacturer’s instructions using an NK cell magnetic isolation kit (STEMCELL Technologies). NK cells were cultured for at least 24 h before conducting experiments. For killing experiments, NK cells were cultured for 24 h in complete medium containing 0.2–0.5 μg ml^−1^ interleukin-2 (BioLegend).

### NK cell killing assays

Target cells were collected by centrifugation and resuspended in serum-free RPMI containing 5 μM CellTracker Far Red (Thermo Fisher Scientific) at 5 × 10^5^ cells per ml. Cells were then incubated for 30 min at 37 °C; where indicated, cells were treated with 10–20 nM StcE. Following staining and StcE treatment, cells were centrifuged, washed twice with PBS containing 1 mM EDTA and resuspended in complete medium. Cells were diluted to 1 × 10^5^ cells per ml in complete medium containing 100 nM Sytox Green, and 100 μl of cell suspension was aliquoted into aflat-bottom 96-well plate. Separately, NK cells were diluted to various cell concentrations to generate the indicated effector:target ratios in complete medium containing 100 nM Sytox Green. Where indicated, these cell suspensions were treated with 20 nM StcE for 30 min, washed twice with PBS containing 1 mM ETDA and resuspended in complete medium containing 100 nM Sytox Green; 100 μl of these cell suspensions was then mixed with the target cell suspensions to generate a total volume of 200 μl. Cells were incubated at 37 °C for 4 h and analyzed by flow cytometry.

For mixed cell assays, K562^HER2^ cells were collected by centrifugation and incubated for 30 min at 37 °C in serum-free RPMI containing 0.33 μM CellTrace Far Red (Thermo Fisher Scientific) at 5 × 10^5^ cells per ml. K562, isolated NK and labeled K562^HER2^ cells were collected by centrifugation and resuspended in complete medium, and 1 × 10^4^ K562 cells, 1 × 10^4^ K562^HER2^ cells and 2 × 10^4^ NK cells in 200 μl of complete medium containing 50 nM Sytox Green were added to a flat-bottom 96-well plate. PBS, αHER2-eStcE or StcE in PBS was added to a final volume of 222.2 μl and incubated for 4 h at 37 °C. Cells were analyzed by flow cytometry using a BD Accuri C6 Plus such that a minimum of approximately 2,000 K562 cells and 2,000 K562^HER2^ cells were analyzed. For the gating strategy, see Supplementary Fig. 4.

### Bioactive compound library screen

A library of 261 bioactive compounds (Selleck Chemicals) was stored at −80 °C. The library was reformatted from 96-well to 384-well format using a Versette automated liquid handler configured with a 96-channel pipetting head and diluted to 2 mM in DMSO. The day before the screen, 5 × 10^3^ OVCAR-3^N^ cells per well were seeded into two 384-well plates in 45 μl of medium. The next day, the medium was removed and replaced with medium containing 20 nM Sytox Green, and compounds from a freshly thawed library master stock plate (one compound per well) were added to a final concentration of 500 nM. One plate was cotreated with vehicle (PBS), and the other plate was treated with 50 nM StcE. Plates were imaged immediately and every 2 h thereafter for a total of 72 h using an Essen IncuCyte Zoom. Counts of Sytox Green^+^ and mKate2^+^ objects per mm^2^ were obtained, and the lethal fraction was calculated as previously described^26^. The area under the curve (AUC) values of lethal fraction scores across the full 72 h were calculated using the trapezoid rule in Excel (Microsoft). The Bliss independence model was used to compute expectant cell death of compound and StcE treatment using normalized AUC values, and deviation from this expectation was used to infer modulation of cell death, as described previously^63^.

### Measuring cell death using STACK

Follow-up cell death experiments of OVCAR-3^N^ cells were performed using STACK^26^. Cell lines stably expressing nuclear-localized mKate2 were incubated in medium containing 20 nM Sytox Green. Counts of live (mKate2^+^) and dead (Sytox Green^+^) objects were obtained from images collected every 2 or 4 h. The following image extraction parameter values were used to count OVCAR-3^N^ mKate2^+^ objects: parameter adaptive, threshold adjustment of 1; edge split on; edge sensitivity of 50; filter area minimum of 20 μm^2^ and maximum of 8,100 μm^2^ and eccentricity maximum of 1.0. The following image extraction parameter values were used to count OVCAR-3^N^ Sytox Green^+^ objects: parameter adaptive, threshold adjustment of 10; edge split on; edge sensitivity of −5; filter area minimum of 20 μm^2^ and maximum of 750 μm^2^ and eccentricity maximum of 0.9. Counts were exported to Excel, and lethal fraction scores were computed from mKate2^+^ and Sytox Green^+^ counts as previously described^26^. To compute lethal fraction, double mKate2^+^Sytox Green^+^ counts were subtracted from live-cell counts.

### ColabFold modeling

Protein sequences for StcE and αHER2-eStcE were used as input for ColabFold (https://colab.research.google.com/github/sokrypton/ColabFold/blob/main/AlphaFold2.ipynb#scrollTo=kOblAo-xetgx)^62^. The predicted structure for StcE aligned well (for the previously reported StcE structure^35^, root mean squared deviation (r.m.s.d.) = 0.2) with the X-ray structure determined by Yu et al.^35^. Molecular graphics were generated using PyMOL.

### Molecular modeling

The AlphaFold-predicted structure of StcE^64^ was overlaid with a glycopeptide–StcE model complex^18^ previously generated via docking experiments with the crystal structure originally determined by Yu et al.^35^. As such, the unaltered glycopeptide ligand Ac-P(GalNAc)TLTH-*N*Me and zinc ion of the docked complex underwent brief minimization with the AlphaFold structure using the Amber10:EHT forcefield^65^ in Molecular Operating Environment to yield the final complex used to inform mutagenesis studies.

### Cloning

StcE mutants were cloned from pET28b-StcE_Δ35-NHis, generously provided by N. Strynadka (University of British Columbia), using a Q5 site-directed mutagenesis kit (New England Biolabs), In-Fusion HD Cloning Plus (Takara Bio) or by ordering related designed plasmids from Twist Bioscience. The amino acid sequences for the 5F7 nanobody (provided by M. Gray, Stanford University^66^) and αGFP (previously published^44^) were reverse translated and optimized for expression in *E. coli* K12 with the IDT Codon Optimization Tool before cloning as described above. A plasmid containing the sequence for TP1107 (αIgG1) was ordered from Addgene (plasmid 104158) and cloned into the plasmid as described above.

### Protein purification

BL21(DE3) *E. coli* cells were transformed with sequence-confirmed plasmids and grown in sterile terrific broth with 30 μg ml^−1^ kanamycin at 37 °C and 250 r.p.m. until an optical density of 0.4–0.8 was reached. Protein expression was induced with 0.3 mM isopropyl-β-D-1-thiogalactopyranoside, and the culture was incubated overnight at 20 °C and 250 r.p.m. Cells were centrifuged at 6,000*g* for 10 min and lysed in 20 mM HEPES (pH 7.5) and 500 mM NaCl with a probe tip sonicator. Lysates were clarified by centrifuging at 11,000*g* for 10 min and filtered through a low-protein-binding 0.22-μm polyethersulfone membrane vacuum filter bottle (Corning). Lysates were applied to 3–4 ml of Ni-NTA agarose (Qiagen) per liter of bacterial culture, washed with 200 ml of 20 mM HEPES (pH 7.5), 500 mM NaCl and 20 mM imidazole and eluted with 20 ml of 20 mM HEPES (pH 7.5), 500 mM NaCl and 250 mM imidazole per liter of culture. Purified proteins were buffer exchanged into cold PBS either with Zeba Spin desalting columns (7,000-Da molecular weight cutoff (MWCO) and 0.5-ml capacity (Fisher Scientific)) or through dialysis with Pierce Slide-A-Lyzer G2 dialysis cassettes (20,000-Da MWCO (Fisher Scientific)). Protein concentration was determined via NanoDrop, and protein purity was determined by SDS–PAGE. Purified protein aliquots were stored at −80 °C and thawed and stored at 4 °C before experiments.

### Endotoxin-free protein purification

ClearColi BL21(DE3) electrocompetent cells (Lucigen) were transformed with plasmids and grown in sterile LB-Miller culture medium with 30 μg ml^−1^ kanamycin at 37 °C and 250 r.p.m. until an optical density of 0.4–0.8 was reached. Protein expression was induced with 0.4 mM IPTG, and the proteins were purified as described above. Proteins were run through Pierce high-capacity endotoxin removal columns (Thermo Fisher Scientific) at least four times, following the manufacturer’s instructions. Endotoxin levels were tested using a HEK-Blue LPS detection kit 2 (InvivoGen) according to the manufacturer’s recommendations. All endotoxin levels were confirmed to be below *K*/*M* for the maximum dose used in vivo, where *K* is 5 EU kg^−1^, and *M* is the dose of the protein/formulation of interest within a 1-h period^67^.

### In vitro mucin cleavage activity assays

Recombinant C1-INH (Molecular Innovations) was labeled with IRDye 800CW NHS Ester (LI-COR Biosciences; dye:protein ratio of 0.54) following the manufacturer’s instructions. Extra dye was removed with Zeba Spin desalting columns (7,000-Da MWCO and 0.5-ml capacity (Fisher Scientific)). For each reaction, 50 nM mucinase and 500 nM recombinant mucin in PBS were combined and incubated at 37 °C for 1 h, and 4× NuPAGE LDS sample buffer (Fisher Scientific) and dithiothreitol (Thermo Fisher Scientific) were added to final concentrations of 1–2× and 1–250 mM, respectively, and boiled at 95 °C for 5 min. Samples were run on a 4−12% 18-well Criterion XT Bis-Tris protein gel (Bio-Rad) in XT-MOPS (Bio-Rad) at 180 V for 1 h. Gels were imaged using an Odyssey CLx near-infrared fluorescence imaging system (LI-COR Biosciences). To quantify mucinase activity, product band and C1-INH parent band signal intensities were determined using Image Studio software, and digestion percentage was calculated by dividing the signal from the product bands by the signal from the parent and product bands. For recombinant human MUC16 digestion experiments, recombinant MUC16 (R&D Systems) was left unlabeled but was otherwise treated as described above. Protein was visualized with AcquaStain protein gel stain (Bulldog-Bio) and imaged using an Odyssey CLx near-infrared fluorescence imaging system. For comparison of in vitro substrate digestion efficiency of different mutants, 89 μg ml^−1^ unlabeled substrates and 162 nM mucinase (StcE, StcE^W366A^, ddStcE, eStcE or αHER2-eStcE) in PBS were combined and incubated overnight at 37 °C. SDS–PAGE gels were run and analyzed as described above. BSA was purchased from Sigma-Aldrich (A7906–1KG), and fetuin was purchased from Promega (V4961). Recombinantly expressed MUC16, podocalyxin, CD43 and PSGL-1 were purchased from R&D Systems (5609-MU, 1658-PD, 9680-CD and 3345-PS, respectively.

### In vitro MUC1 cleavage activity assay

HeLa cells resuspended at 4.5 × 10^5^–5.0 × 10^5^ cells in 150 μl of complete medium were allocated per well toa 96-well, ultralow-attachment round-bottom plate (Corning). Fifty microliters of the different StcE mutants in PBS was added to the wells, and the plate was incubated at 37 °C for 1 h. Cells were washed twice with cold FACS buffer containing 2 mM EDTA and once with cold FACS buffer and then stained with anti-MUC1/episialin (clone 214D4; EMD Millipore) in FACS buffer supplemented with 0.1% benzonase for 30 min on ice. Cells were washed three times with cold FACS buffer containing 2 mM EDTA and stained with Alexa Fluor 647 Affinipure goat anti-mouse IgG ( Jackson ImmunoResearch). Cells were washed twice with FACS containing 2 mM EDTA and stained with 30 nM Sytox Green for 10 min at 4 °C. A minimum of 30,000 ungated events were analyzed using a BD Accuri C6 Plus. For data analysis, the Alexa Fluor 647 MFI values of unstained and PBS-treated samples were used to define 0% and 100% cell-surface MUC1, respectively, and each sample was normalized to percent MUC1 within each replicate. Using GraphPad Prism 9, each replicate was fitted to inhibitor concentration versus normalized response, and log_10_ (half-maximal inhibitory concentration) was reported.

### Mucinase binding assays to cell-surface mucins

HeLa cells (4 × 10^5^–5 × 10^5^ cells) were added to each well of a V-bottom 96-well plate and washed three times with cold FACS buffer containing 2 mM EDTA. Cells were treated with mucinases in cold FACS buffer containing 2 mM EDTA and 0.1% benzonase for 30 min on ice, washed three times with FACS buffer with 2 mM EDTA and stained with FITC–anti-His (clone GG11–8F3.5.1; Miltenyi Biotec) for 30 min on ice. Cells were washed three times with FACS buffer with 2 mM EDTA and stained with 5 nM Sytox Red in FACS buffer with 2 mM EDTA for 20 min. Approximately 10,000 live single cells were analyzed using a MACSQuant Analyzer 10 flow cytometer (Miltenyi Biotec). Within each sample, binding was normalized to the greatest MFI per mucinase, with 0 being defined as the MFI for the PBS-treated sample. To mitigate the hook effect, only concentrations with greater than 85% normalized binding past the maximum signal were included. In GraphPad Prism 9, each replicate was fitted to agonist concentration versus response with the lower limit restricted to 0, and log_10_ (EC_50_) was reported.

For conjugate and nanobody binding assays, MCF10A^±MUC1 ±HER2^ cells were processed, washed and stained as described above. Approximately 20,000 live single cells were analyzed on a BD Accuri C6 Plus. For replicates in which Prism could not correctly fit the data to report an EC_50_ value, the replicate was not included in the bar graph of EC_50_ values; this occurred with one replicate of αHER2-eStcE binding to MCF10A cells.

### eStcE MS sample preparation

Recombinantly expressed MUC16, podocalyxin, CD43 and PSGL-1 were purchased from R&D Systems (5609-MU, 1658-PD, 9680-CD and 3345-PS, respectively). All recombinant mucin-domain glycoproteins were reconstituted in ultrapure water (Pierce) to a concentration of 1 mg ml^−1^. A fraction (1 μg; 1 μl) of each recombinant glycoprotein was digested with eStcE at a 1:1 enzyme:substrate ratio. For sialidase-treated samples, 1 μl of sialoEXO (Genovis) was added to 39 μl of ultrapure water, and 1 μl of this dilution was added to the reaction vial, per the manufacturer’s instructions. The reaction was brought to a total volume of 12 μl in 50 mM ammonium bicarbonate and allowed to react overnight at 37 °C. Control reactions were incubated at 37 °C overnight in a solution containing buffer only. The following day, the volume was increased to 19 μl with 50 mM ammonium bicarbonate. PNGaseF (1 μl; Promega) was added to 99 μl of 50 mM ammonium bicarbonate, and 1 μl of this reaction was added to each mucinase reaction vial. De-*N*-glycosylation reactions were incubated for 8−12 h at 37 °C. Reduction and alkylation were performed according to ProteaseMax (Promega) protocols. Briefly, the solution was diluted to 93.5 μl with 50 mM ammonium bicarbonate. Then, 1 μl of 0.5 M dithiothreitol was added, and the samples were incubated at 56 °C for 20 min, followed by the addition of 2.7 μl of 0.55 M iodoacetamide at room temperature for 15 min in the dark. Digestion was completed by adding sequencing-grade trypsin (Promega) at a 1:20 enzyme:substrate ratio overnight at 37 °C and quenched by adding 0.3 μl of glacial acetic acid. C18 cleanup was performed using 1-ml Strata-X columns (Phenomenex). Each column was wet with 1 ml of acetonitrile once, followed by rinsing with 1 ml of buffer A (0.1% formic acid in water). The samples were diluted to 1 ml in buffer A and loaded through the column and rinsed with buffer A. Finally, the samples were eluted with three rinses of 100 μl of buffer B (0.5% formic acid and 80% acetonitrile) and dried by SpeedVac. The samples were reconstituted in 10 μl of buffer A for MS analysis.

### MS for cleavage motif

Samples were analyzed by online nanoflow liquid chromatography–tandem MS (LC–MS/MS) using an Orbitrap Fusion Tribrid mass spectrometer (Thermo Fisher Scientific) coupled to a Dionex Ultimate 3000 HPLC (Thermo Fisher Scientific). Each sample was analyzed twice, once with a higher-energy collisional dissociation (HCD)-triggered electron transfer dissociation (ETD) method (for input to Byonic) and once with an HCD-triggered electron-transfer/higher-energy collision dissociation (EThcD) method (for input into O-Pair). A portion of the sample (4 of 10 μl (40%)) was loaded via autosampler isocratically onto a C18 nano precolumn using 0.1% formic acid in water (solvent A). For preconcentration and desalting, the column was washed with 2% acetonitrile and 0.1% formic acid in water (loading pump solvent). Subsequently, the C18 nano precolumn was switched in line with the C18 nano separation column (75 μm × 250 mm EASY-Spray containing 2-μm C18 beads) for gradient elution. The column was held at 40 °C using a column heater in the EASY-Spray ionization source (Thermo Fisher Scientific). The samples were eluted at a constant flow rate of 0.3 μl min^−1^ using a 90-min gradient. The gradient profile was as follows (presented in minutes:percent solvent B; 2% formic acid in acetonitrile): 0:3, 3:3, 93:35, 103:42, 104:95, 109:95, 110:3 and 140:3. The instrument method used an MS1 resolution of 60,000 full-width at half-maximum at 400 *m*/*z*, an automatic gain control (AGC) target of 3 × 10^5^ and a mass range from 300 to 1,500 *m*/*z*. Dynamic exclusion was enabled with a repeat count of 3, repeat duration of 10 s and exclusion duration of 10 s. Only charge states 2 to 6 were selected for fragmentation. MS2s were generated at top speed for 3 s. HCD was performed on all selected precursor masses with the following parameters: isolation window of 2 *m*/*z*, 30% collision energy, orbitrap detection (resolution of 30,000) and an AGC target of 1 × 10^4^ ions. For HCD-pd-ET(hc)D runs, ET(hc)D was performed if (1) the precursor mass was between 300 and 1,000 *m*/*z* and (2) three of nine HexNAc or NeuAc fingerprint ions (126.055, 138.055, 144.07, 168.065, 186.076, 204.086, 274.092 and 292.103) were present at ±0.1 *m*/*z* and greater than 5% relative intensity. ETD parameters were as follows: calibrated charge-dependent ETD times, 2 × 10^5^ reagent target and precursor AGC target of 1 × 10^4^. EThcD parameters were the same but included 30 normalized collision energy supplemental activation and Orbitrap analysis at a resolution of 30,000 full-width at half-maximum.

### MS data analysis for cleavage motif

HCD-pd-ETD raw files were searched using Byonic by ProteinMetrics against directed databases containing the recombinant protein of interest. Search parameters included semispecific cleavage specificity at the C-terminal site of R and K, meaning non-tryptic cleavage was permitted at either the N or C terminus of a detected peptide but not both. Mass tolerance was set at 10 ppm for MS1s, 0.1 *m*/*z* for HCD MS2s and 0.35 *m*/*z* for ETD MS2s. Methionine oxidation (common 2), asparagine deamidation (common 2) and N-terminal acetylation (rare 1) were set as variable modifications with a total common maximum of 3 and rare maximum of 1. *O*-Glycans were also set as variable modifications (common 2) using ‘*O*-glycan 6 most common’ structures^18^. Cysteine carbaminomethylation was set as a fixed modification. Peptide hits were filtered using a 1% false discovery rate. All peptides were manually validated and/or sequenced using Xcalibur software (Thermo Fisher Scientific). HCD was used to confirm that the peptides were glycosylated, and ETD spectra were used for localization of glycosylation sites.

To further confirm eStcE cleavage sites, HCD-pd-EThcD raw files were searched using O-Pair in MetaMorpheus against directed databases containing the recombinant protein of interest^68^. Search parameters included an ‘*O*-glycopeptide search’ using the ‘Oglycan. gdb’ database. The top 50 candidates were kept, using HCD-pd-EThcD fragmentation, with a maximum of four glycans allowed. Semitrypsin cleavage specificity was selected with a maximum of two missed cleavages and a peptide length of 5–60 amino acids. Mass tolerance was set at 10 ppm for MS1s and 20 ppm for all MS2s. Cysteine carbaminomethylation was set as a fixed modification, and methionine oxidation and asparagine deamidation were set as variable modifications. O-Pair results were filtered for results with a *q* value of less than 0.01.

### TAILS MS sample preparation

TAILS methods were adapted from previous TAILS publications^38,69^ and protocols available at the Overall group website (https://clip.ubc.ca/resources/protocols-and-sops/; Bench Protocol v5.6).

K562^HER2^ cells were washed three times with warmed PBS, incubated for 1–2 h in serum-free RPMI without phenol red and without glutamine and resuspended in the same medium at 0.8 × 10^6^ cells per ml. αHER2-eStcE, eStcE, StcE or equal volume PBS was added to a final concentration of 1 nM, and the cells were incubated overnight at 37 °C. Cells were centrifuged at 600*g* for 5 min, and conditioned supernatant was collected and treated with protease inhibitors (cOmplete EDTA-free protease inhibitor cocktail) and 10 mM EDTA. Conditioned supernatant was clarified by centrifugation at 1,000*g* for 5 min at 4 °C. Trichloroacetic acid was added to a final concentration of 15% (vol/vol), and the mixture sat on ice for 3–4 h. Precipitated proteins were washed three times by repeated pelleting by centrifugation at 9,000*g* for 15 min at 4 °C, decanting of the supernatant and resuspending the pellets in 100% acetone (−20 °C). After the final spin, the supernatant was decanted, and the pellets were frozen overnight.

Pellets were resuspended in 100 μl of 6 M guanidine hydrochloride, and protein amounts were determined by a bicinchoninic acid protein assay kit (Thermo Fisher Scientific); 125 μg of total protein material was used for each sample, and samples were adjusted to a total volume of 175 μl with water. Samples were then adjusted to 100 mM HEPES before adding freshly prepared TCEP to a final concentration of 10 mM and incubating at 37 °C for 30 min. Freshly prepared *N*-ethylmaleimide (NEM; adjusted to pH 6) was added to a final concentration of 15 mM, and samples were incubated for 10 min in the dark at room temperature. Fifty microliters of 100 mM TCEP was added, and samples were vortexed to quench NEM before samples were put on ice. Proteins were precipitated using acetone, where a fourfold volume of ice-cold acetone was added to each sample in an acetone-compatible tube. Samples were vortexed and incubated at −80 °C overnight before centrifuging at 18,000*g* for 10 min. Supernatant was decanted, with care taken not to disturb the protein pellet, and pellets were air dried for 15 min in an uncapped tube. Samples were then labeled with 16-plex tandem mass tags (TMT; Thermo Fisher Scientific) according to the labeling scheme in Supplementary Table 7, with distinct differences from manufacturer protocols because of labeling at the protein rather than peptide level. Samples were resuspended in 110 μl of 100 mM TEAB, and TMT labels (0.8 mg each) were dissolved in 110 μl of DMSO. Samples were vortexed before a 1-h incubation in the dark at 25 °C. Following incubation, samples were combined into a single tube, and proteins were precipitated using the same acetone precipitation protocol described above with overnight incubation. Following the 15-min air dry, the pellet was resuspended in 50 μl of 6 M guanidine hydrochloride and diluted tenfold with 100 mM HEPES (pH 8.0). Trypsin was added at a protease:protein ratio of 1:100, with gentle mixing with a pipette before overnight incubation at 37 °C. Negative selection for N-terminal peptides was performed using 45 mg ml^−1^ HPG-ALDII obtained from the Overall lab (29-mg aliquot). The HPG-ALDII polymer was thawed at room temperature and added to the digested sample at a polymer:peptide ratio of 6:1. Sodium cyanoborohydride was immediately added to a final concentration of 20 mM, and the sample was gently mixed, the pH was confirmed to be ~6–7, and the sample was incubated overnight at 37 °C. Sample recovery was performed the following day, with all centrifugation steps at 12,000*g* for 10 min at room temperature. Tris (pH 6.8) was added to a final concentration of 100 mM, a pH of 6–7 was verified, and the sample was incubated for 30 min at 37 °C. A 10-kDa MWCO Amicon column was prewashed with 400 μl of 100 mM NaOH and 400 μl of water, and flow-through (FT) was discarded. The peptide–polymer mixture was centrifuged through the column, and the FT was collected into a clean tube (TAILS sample 1). The filter was then washed by spinning 400 μl of water through, the FT was added to TAILS sample 1, and the filter was thoroughly washed with 100 μl of water, which rids the filter of the very hydrophilic polymer. The filter was repositioned upside down in a new tube (TAILS sample 2), with a quick spin to increase the yield of hydrophobic peptides. TAILS samples 1 and 2 were lyophilized before they were desalted using 10 mg ml^−1^ Strata-X columns (Phenomenex). Briefly, columns were wet with 1 ml of acetonitrile followed by equilibration with 1 ml of 0.2% formic acid in water. Samples were resuspended in 500 μl of 0.2% formic acid in water and loaded on the column, followed by a wash with 1 ml of 0.2% formic acid. Peptides were eluted with 400 μl of 0.2% formic acid in 80% acetonitrile, dried via lyophilization and resuspended in 0.2% formic acid in water before MS analysis.

### TAILS LC-MS/MS

Both TAILS samples 1 and 2 were analyzed using 90-min LC–MS/MS acquisitions, and TAILS sample 1 was analyzed with an additional 240-min LC–MS/MS acquisition. Peptide mixtures were separated over a 25-cm EASY-Spray reversed-phase LC column (75-μm inner diameter packed with 2-μm, 100-Å PepMap C18 particles; Thermo Fisher Scientific). The mobile phases (A: water with 0.2% formic acid; B: acetonitrile with 0.2% formic acid) were driven and controlled by a Dionex Ultimate 3000 RPLC nano system (Thermo Fisher Scientific). An integrated loading pump was used to load peptides onto a trap column (Acclaim PepMap 100 C18, 5-μm particles, 20-mm length; Thermo Fisher Scientific) at 5 μl min^−1^, which was put in line with the analytical column 5.5 min into the gradient. Gradient elution was performed at 300 nl min^−1^ for all analyses. For the 90-min acquisitions, the gradient was held at 0% B for the first 6 min of the analysis, followed by an increase from 0% to 5% B from 6 to 6.5 min, an increase from 5% to 22% B from 6.5 to 66.5 min, an increase from 22% to 90% B from 66.5 to 70 min, isocratic flow at 90% B from 70 to 75 min and a reequilibration at 0% B for 15 min. For the 240-min acquisitions, the gradient was held at 0% B for the first 6 min of the analysis, followed by an increase from 0% to 5% B from 6 to 6.5 min, an increase from 5% to 25% B from 6.5 to 200 min, an increase from 25% to 90% B from 200 to 218 min, isocratic flow at 90% B from 218 to 224 min and a reequilibration at 0% B for 16 min. For all methods, eluted peptides were analyzed on an Orbitrap Fusion Tribrid MS system (Thermo Fisher Scientific). Precursors were ionized using an EASY-Spray ionization source (Thermo Fisher Scientific) held at +2.2 kV compared to ground, and the column was held at 40 °C. The inlet capillary temperature was held at 275 °C. Survey scans of peptide precursors were collected in the Orbitrap from 350 to 1,500 Th, with an AGC target of 250% (1,000,000 charges), a maximum injection time of 50 ms and a resolution of 60,000 at 200 *m*/*z*. For 90-min analyses, monoisotopic precursor selection was enabled for peptide isotopic distributions, precursors of *z* = 2–5 were selected for data-dependent MS/MS scans for 2 s of cycle time, and dynamic exclusion was set to 30 s with a ±10-ppm window set around the precursor monoisotope. An isolation window of 1 Th was used to select precursor ions with the quadrupole. MS/MS scans were collected using HCD at 30 normalized collision energy with an AGC target of 200% (100,000 charges) and a maximum injection time of 118 ms. Mass analysis was performed in the Orbitrap with a resolution of 60,000 with a first mass set at 120 *m*/*z*. All sets were the same for 240-min analyses, with the exception of a 3-s cycle time and a 60-s dynamic exclusion time.

### TAILS MS data analysis

All raw data files were processed in batch using MaxQuant^70^, where the Andromeda search engine^71^ was used to search the entire human proteome downloaded from UniProt (reviewed 20,428 entries). Cleavage specificity was set to ‘semi-specific free N-terminus’ with ArgC specificity. The NEM modification of cysteine had to be created, with an addition of C_6_H_7_O_2_N (125.0478 Da) (ref. 72), and was set as a fixed modification, while oxidation of methionine was set as a variable modification with five maximum modifications per peptide. The experiment type was set to Reporter ion MS2 with 16-plex TMT modifications selected (user defined modifications were added for both lysine and N-terminal labeling). The reporter ion mass tolerance was set to 0.003 Da, and the minimum reporter precursor ion fractions (PIF) score was set to 0.75. Defaults were used for the remaining settings, including peptide-to-spectrum match (PSM) and protein false discovery rate thresholds of 0.01 and 20 ppm, 4.5 ppm and 20 ppm for first search MS1 tolerance, main search MS1 tolerance and MS2 product ion tolerance, respectively. ‘Match between runs’ and ‘second peptide’ options were not enabled. Quantified peptides were then processed in Perseus^73^. Contaminants and reverse hits were removed, and signal in all relevant TMT channels of at least one condition was required to retain protein identifications. Significance calculations for TAILS comparisons were performed using a two-tailed *t*-test with multiple testing corrections performed using a permutation-based false discovery rate with 250 randomizations, a false discovery rate of 0.05 and an S0 value of 2. The four proteins specifically degraded by StcE compared to PBS (CD99L2, TNFRSF1B, CD55 and CD46) were manually searched for regions with a high density of predicted mucin-type *O*-glycosylation using NetOGlyc-4.0 (ref. 16). To account for phosphorylated residues incorrectly predicted as glycosylated residues, phosphorylation was annotated using PhosphoSitePlus^74^.

### Generation of HER2 stable lines

pMXs-HER2 vector was generated by cloning the HER2^+^ coding sequence (Addgene) into the pMXs-FLAG backbone using In-Fusion HD Cloning Plus (Takara Bio). HEK-293T cells (1.5 × 10^6^) were seeded into 6-cm dishes in 5 ml of complete medium. Twenty-eight hours later, 1 μg of pMXs-HER2 was mixed with 900 ng of retrovirus Pol/Gag, 150 ng of vesicular stomatitis virus G protein DNA, 130 μl of DMEM and 6 μl of 1 mg ml^−1^ polyethylenimine. The mixture was incubated for 20 min at room temperature and added to HEK-293T cells dropwise. Eighteen hours later, the culture medium was replaced with 5 ml of DMEM supplemented with 30% heat-inactivated FBS and 1% P/S. Thirty hours later, the medium was collected and centrifuged at 1,000 r.p.m. for 5 min. The clarified supernatant was stored at −80 °C before infection. To establish stably expressing cell lines, 1.5 × 10^6^ cells were seeded in six-well plates in 2.8 ml of complete medium. Polybrene was added at 10 μg ml^−1^, and cells were infected with 200 μl of virus-containing medium. Plates were centrifuged at 2,200 r.p.m. for 45 min and incubated at 37 °C and 5% CO_2_. Twelve to 24 h later, cells were lifted with trypsin and plated in 10-cm dishes with 10 μg ml^−1^ blasticidin S (Thermo Fisher Scientific). Stable cell lines were tested for expression of HER2 by flow cytometry.

### HER2 flow cytometry

Log-phase cells were aliquoted into a V-bottom 96-well plate at 5 × 10^5^ cells per well. Cells were washed twice with cold FACS buffer with 2 mM EDTA and once with cold FACS buffer and then stained with Alexa Fluor 488 or Alexa Fluor 647 anti-human CD340 (erbB2/HER2; 24D2 clone) in FACS buffer containing 0.1% benzonase on ice protected from light. Cells were washed three times with FACS buffer with 2 mM EDTA and stained with 5 nM Sytox Red or 30 nM Sytox Green for 10 min at 4 °C. A minimum of 30,000 ungated events were analyzed using a BD Accuri C6 Plus.

### K562 mixed cell CD43 cleavage assay

K562 and K562^HER2^ cells (2.5 × 10^5^ cells each) were allocated per well in a 96-well, ultralow-attachment round-bottom plate in 150 μl of complete medium. Fifty microliters of mucinases in PBS was added to the wells, and the plate was incubated (overnight unless stated otherwise) at 37 °C. Cells were washed twice with cold FACS buffer with 2 mM EDTA and once with cold FACS buffer and then stained with Alexa Fluor 488 anti-human CD340 (erbB2/HER2; 24D2 clone; BioLegend) and Alexa Fluor 647 anti-CD43/sialophorin (MEM-59 clone; Novus Biologicals) in FACS buffer supplemented with 0.1% benzonase on ice protected from light. Cells were washed three times with cold FACS buffer with 2 mM EDTA and stained with 1 μM Sytox AADvanced (Thermo Fisher Scientific) or 1 μM Sytox Blue in FACS buffer with 2 mM EDTA for 5 min on ice. At least 20,000 live single cells (representing approximately 10,000 of each population) were analyzed using a BD Accuri C6 Plus or MACSQuant Analyzer 10 flow cytometer (Miltenyi Biotec). Samples were compensated using single-stained controls in FlowJo v. 10.0. Unstained and PBS-treated samples were used to define 0% and 100% cell-surface CD43, respectively, and each sample was normalized to percent CD43 within each replicate. Using GraphPad Prism 9, replicates were fitted to inhibitor concentration versus normalized response.

For the experiment validating αIgG1-eStcE cutting in a mixed cell assay, K562^HER2^ cells were stained with 1.25 μM CellTrace Violet (Thermo Fisher Scientific) in RPMI at 37 °C for 20 min and quenched with complete medium for 5 min. Then, 2.5 × 10^5^ K562 and CellTrace Violet-stained K562^HER2^ cells were mixed and treated as described above with αHER2-eStcE or αIgG1-eStcE and 10 μg ml^−1^ mouse IgG1 anti-human CD340 (erbB2/HER2; 24D2 clone; BioLegend). Cells were stained and analyzed as described above, except no Alexa Fluor 488 anti-HER2 was used.

### MCF10A^MUC1^ mixed cell MUC1 cleavage assay

MUC1ΔCT was induced with 1 μg ml^−1^ doxycycline for 24 h. HER2^+^ cells were stained with 5 μM Molecular Probes CellTracker Green CMFDA dye (Thermo Fisher Scientific) in PBS at 37 °C for 30 min and washed twice with warmed PBS. Then, 2.5 × 10^5^ MCF10A^MUC1, HER2^ and 2.5 × 10^5^ MFC10A^MUC1^ cells (matched for induction or no induction with doxycycline) were added to each well of a low-adhesion, U-bottom 96-well plate in 150 μl of complete medium. Fifty microliters of mucinases in PBS was added to wells, and the plate was incubated overnight at 37 °C and 5% CO_2_. Cells were washed twice with cold FACS buffer with 2 mM EDTA and once with cold FACS buffer and then stained with MUC1 mouse monoclonal antibody (clone VU4H5; Cell Signaling Technology) in FACS buffer supplemented with 0.1% benzonase on ice for 30 min. Cells were washed three times with cold FACS buffer with 2 mM EDTA, stained with Alexa Fluor 647 Affinipure goat anti-mouse IgG ( Jackson ImmunoResearch) for 30 min on ice, washed three times with cold FACS buffer with 2 mM EDTA and stained with 1 μM Sytox AADvanced (Thermo Fisher Scientific) in FACS buffer with 2 mM EDTA for 5 min on ice. Approximately 30,000 ungated events were analyzed using a BD Accuri C6 Plus.

### Kinetics of αHER2-eStcE binding and cutting

At different time points, 150 μl of complete medium containing 5 × 10^5^ K562^HER2^ cells and 50 μl of 400 nM αHER2, αHER2-eStcE or StcE in PBS were added to a 96-well, ultralow-attachment round-bottom plate and incubated at 37 °C. At the end of the incubation (times reported are total incubation length, and incubation was stopped for all time points simultaneously), the cells were washed twice with cold FACS buffer and stained with FITC–anti-His (clone GG11–8F3.5.1; Miltenyi Biotec) and Alexa Fluor 647 anti-CD43/sialophorin (MEM-59 clone; Novus Biologicals) for 30 min in FACS buffer supplemented with 0.1% benzonase on ice protected from light. Cells were washed twice with cold FACS buffer with 2 mM EDTA and stained with 1 μM Sytox Blue in FACS buffer with 2 mM EDTA for 5 min on ice. At least 20,000 live single cells were analyzed using a MACSQuant Analyzer 10 flow cytometer (Miltenyi Biotec). At each time point, unstained and PBS-treated samples were used to define 0% and 100% cell-surface CD43, respectively, and each sample was normalized to percent CD43 within each replicate. Using GraphPad Prism 9, replicates were fitted to inhibitor concentration versus normalized response.

### αIgG1-eStcE binding to K562^HER2^

αIgG1-eStcE and αHER2-eStcE were labeled with Alexa Fluor 647 NHS Ester (Thermo Fisher Scientific) following the manufacturer’s instructions and mixed with unlabeled protein for a final consistent dye:protein ratio of 0.819. K562^HER2^ cells were stained with 1.25 μM CellTrace Violet as described above. Then, 2.5 × 10^5^ K562 and CellTrace Violet-stained K562^HER2^ cells were added to each well of a V-bottom 96-well plate and washed three times with cold FACS buffer. Cells were stained for 30 min with 5 μg ml^−1^ mouse IgG1 anti-human CD340 (erbB2/HER2; 24D2 clone; BioLegend) or were left unstained in FACS buffer and 0.1% benzonase for 30 min on ice. Cells were washed three times with FACS buffer with 2 mM EDTA, stained with the indicated concentrations of Alexa Fluor 647-labeled αIgG1-eStcE or Alexa Fluor 647-labeled αHER2-eStcE for 30 min on ice, washed three times with FACS buffer with 2 mM EDTA and stained with 30 nM Sytox Green (Thermo Fisher Scientific) in FACS buffer with 2 mM EDTA for at least 5 min. Approximately 40,000 live single cells were analyzed using a MACSQuant Analyzer 10 flow cytometer (Miltenyi Biotec). Within each sample, binding was normalized to the greatest MFI per conjugate, with 0 being defined as the MFI for the PBS-treated sample. To mitigate the hook effect, only concentrations with greater than 85% normalized binding past the maximum signal were included. In GraphPad Prism 9, each replicate was fitted to agonist concentration versus response with the lower limit restricted to 0, and log_10_ (EC_50_) was reported.

### Selection and validation of primary antibodies for αIgG1-eStcE cutting

The K562 mucinome^40^ was used to categorize antigens for targeting with a mixture of primary IgG1 antibody and αIgG1-eStcE. Antigens were categorized as mucins (positive mucinome enrichment score and classified as a mucin), mucin associated (positive mucinome enrichment score but not classified as a mucin) or non-mucin associated (negative mucinome enrichment score and not classified as a mucin). To test the sensitivity of primary antibody binding to mucinase treatment, K562^HER2^ cells were treated with 100 nM StcE or PBS for 1 h at 37 °C and aliquoted at 2.5 × 10^5^ cells per well into a 96-well V-bottom plate. Cells were washed twice with cold FACS buffer and stained with 1.25–20 μg ml^−1^ of each antibody in FACS buffer containing 0.1% benzonase for 30 min on ice. Cells were washed twice with FACS buffer and stained with 20 μg ml^−1^ Alexa Fluor 647 goat anti-mouse IgG1 (Invitrogen) in FACS buffer for 30 min on ice protected from light. Cells were washed twice with cold FACS buffer containing 2 mM EDTA, stained with 30 nM Sytox Green for 10 min and analyzed using a MACSQuant Analyzer 10 flow cytometer (Miltenyi Biotec). For each antibody, the maximum MFI value achieved at any concentration was reported, and minimum concentration of antibody that achieved approximately 95% staining was used for CD43 cutting assays to minimize oversaturation of the primary antibody. The isotype control did not bind at any concentration; so 20 μg ml^−1^ was used in the cutting experiments to match the highest concentration used for any antibody (Supplemental Table 8).

### αIgG1-eStcE cutting CD43 on K562^HER2^

K562^HER2^ cells were allocated in a 96-well, ultralow-attachment round-bottom plate (5 × 10^5^ cells per well) in 150 μl of complete medium. Fifty microliters of mucinases or the indicated concentration of primary antibody (Supplemental Table 8) and αIgG1-eStcE in PBS was added to wells, and the plate was incubated for 4 h at 37 °C. Cells were washed twice with cold FACS buffer and stained with Alexa Fluor 647 anti-CD43/sialophorin (MEM-59 clone; Novus Biologicals) in FACS buffer supplemented with 0.1% benzonase on ice protected from light. Cells were washed twice with cold FACS buffer with 2 mM EDTA and stained with 30 nM Sytox Green in FACS buffer with 2 mM EDTA for 5 min on ice. At least 20,000 live single cells were analyzed on a MACSQuant Analyzer 10 flow cytometer (Miltenyi Biotec). Unstained and PBS-treated samples were used to define 0% and 100% cell-surface CD43, respectively, and each sample was normalized to percent CD43 within each replicate. For the CD43 primary antibody sample, 100% cell-surface CD43 was defined using a sample treated with the anti-CD43 primary and 0 nM αIgG1-eStcE. Using GraphPad Prism 9, replicates were fitted to inhibitor concentration versus normalized response to generate EC_50_ values of cutting.

### Macrophage phagocytosis assay

MCF7 and MCF7^HER2^ cells were lifted with enzyme-free cell dissociation buffer and resuspended in PBS. MCF7 and MCF7^HER2^ cells were incubated in 5 μg ml^−1^ Alexa Fluor 546 C_5_ maleimide (Invitrogen) and 5 μg ml^−1^ Alexa Fluor 647 C_2_ maleimide (Invitrogen), respectively, for 20 min with rotation at room temperature. Cells were resuspended in 5 mM *N*-ethyl-maleimide (Sigma-Aldrich) in PBS and incubated for 20 min with rotation at room temperature. Cells were resuspended in PBS and treated with PBS, 5 nM endotoxin-free StcE or 100 nM αHER2-eStcE as appropriate for 2 h at 37 °C. After 1 h, InVivoMAb anti-mouse/human/rat CD47 (clone MIAP410; BioXCell) was added to a final concentration of 20 μg ml^−1^. The medium for the macrophages was replaced with serum-free RPMI, and appropriate wells were treated with 10 μM cytochalasin D (Invitrogen). MCF7 and MCF7^HER2^ cells were washed twice with PBS and resuspended in 100 μl of serum-free RPMI. Macrophage medium was replaced with 200 μl of serum-free RPMI. MCF7 and MCF7^HER2^ cells of the same treatment group were mixed, added to the appropriate macrophage well and incubated for 30 min at 37 °C. After incubation, macrophages were gently washed five times with cold PBS. Cells were fixed with 4% paraformaldehyde in PBS for 15 min at room temperature, rinsed with PBS and permeabilized with 0.5% Triton X-100 in PBS for 10 min. Cells were rinsed with PBS, blocked with 2% BSA in PBS for 20 min and stained with Alexa Fluor 488 phalloidin (Invitrogen; 1:2,000) and 7.5 μM DAPI in PBS for 20 min at room temperature. Cells were washed three times with PBS and stored in PBS at 4 °C before imaging with a Nikon A1R confocal microscope. Phagocytosis binding indices were calculated as the surface area of target cells divided by the number of macrophages in the field of view. Surface area and number of macrophages were calculated using the imaging software Imaris. Normalized binding indices were calculated relative to the binding index of the PBS treatment condition of the appropriate biological replicate. Three biological replicates were done with macrophages isolated from three different human donors.

### In vivo toxicity studies

Experiments involving animals were approved under Stanford APLAC protocol number 31511 and number 10266. Animals were housed under the following conditions: 12-h light/12-h dark cycle, ambient temperature of 20–22 °C and relative humidity of between 30 and 70%. Ten-week-old C57BL/6 mice (bred in-house) were injected with PBS or StcE (0.15 mg per kg (body weight) or 15 mg per kg (body weight)) via i.v. injection. Nine-week-old female BALB/cJ mice ( Jackson Laboratory) were injected with PBS or 10 mg per kg (body weight) αHER2-eStcE via i.p. injection. Three hours after injection, mice were submitted to the Necropsy Service at Stanford University for necropsy and histopathologic analysis, and blood samples were submitted to the Animal Diagnostic Laboratory for CBC analyses.

### In vivo biodistribution and mucin degradation western blots

Experiments involving animals were approved under Stanford APLAC protocol number 31511. Twelve-week-old male BALB/cJ mice ( Jackson Laboratory) were injected with PBS, 10 mg per kg (body weight) StcE or 0.25 mg per kg (body weight) IRdye 800CW-labeled StcE via i.p. injection. Liver, spleen, lung and plasma (submandibular bleed) were collected at the indicated times after injection. Tissues were lysed using a Bead Mill 24 homogenizer (Fisher Scientific) in RIPA buffer (Thermo Fisher Scientific) supplemented with benzonase and cOmplete Mini EDTA-free protease inhibitor cocktail tablets (Sigma-Aldrich). Plasma and tissue lysates (40–50 μg) were loaded onto a 4−12% Criterion XT Bis-Tris protein gel and run in XT-MOPS at 180 V for 1 h. Total protein was visualized with AcquaStain protein gel stain (Bulldog-Bio). For mucin western blots, the gel was transferred to a 0.2-μm nitrocellulose membrane using the Trans-Blot Turbo transfer system (Bio-Rad) at 2.5 A for 15 min. Total protein was quantified using REVERT stain (LI-COR Biosciences). The membrane was blocked with carbo-free blocking solution (Vector Laboratories) supplemented with 0.1% (vol/vol) Tween 20 for 1 h at room temperature and incubated with 10 μg ml^−1^ biotin–StcE^E447D^ in PBS with 0.1% (vol/vol) Tween 20 at room temperature for 1 h. IRDye 800CW–streptavidin (LI-COR Biosciences) was used according to the manufacturer’s recommendations. Blots were imaged using an Odyssey CLx near-infrared fluorescence imaging system (LI-COR Biosciences).

Eight-week-old female BALB/cJ mice ( Jackson Laboratory) were injected with PBS or IRdye 680RD–αHER2-eStcE at 0.25, 0.5, 1, 2, 5 and 10 mg per kg (body weight) via retro-orbital injection. Plasma (tail bleed) was collected at *t* = 1, 3, 6, 20 and 48 h after injection. Liver, kidney, spleen, lung and heart tissues for the mice injected with 10 mg per kg (body weight) αHER2-eStcE were collected at *t* = 20 and 48 h after injection. Plasma samples (1 μl) were loaded onto a 4−12% Criterion XT Bis-Tris protein gel and run in XT-MOPS at 180 V for 1 h. Tissues were lysed as described above, and lysates (30 μg) were loaded onto a 4−12% Criterion XT Bis-Tris protein gel and run in XT-MOPS at 180 V for 1 h. Total protein was visualized with AcquaStain protein gel stain (Bulldog-Bio). Gels were imaged using an Odyssey CLx near-infrared fluorescence imaging system. Seven-week-old female BALB/cJ mice ( Jackson Laboratory) were injected with PBS, 5 mg per kg (body weight) StcE or 5 mg per kg (body weight) αHER2-eStcE via retro-orbital injection. Liver, spleen, lung and plasma (submandibular bleed) were collected 4 h after injection. Western blotting for mucin was performed as described above.

### In vivo intestinal permeability assay

Experiments involving animals were approved under Stanford APLAC protocol number 31511. Seven-week-old female BALB/cJ mice ( Jackson Laboratory) were injected with PBS or 10 mg per kg (body weight) αHER2-eStcE via i.p. injection every other day (days 1, 3, 5 and 7) for a total of four doses. On day 8, mice were fasted for 4 h in cages without food or bedding. After fasting, blood was collected via tail vein nick, and 15% (vol/vol) acid citrate dextrose solution (Sigma) was added. Mice were given an oral gavage (150 μl) of 80 mg ml^−1^ FITC–dextran (4 kDa) dissolved in PBS. After 4 h, blood collection was repeated. All blood samples were centrifuged at 5,000 r.p.m. for 10 min to isolate plasma. Plasma samples were diluted 1:10 in 100 μl of PBS and transferred to a black, opaque-bottom 96-well plate. A serial dilution of FITC–dextran (0.2 to 12.5 μg ml^−1^ range) in PBS with 10% (vol/vol) mouse plasma was included for comparison. Fluorescence signal (excitation: 485 nm; emission: 540 nm) was measured using a SpectraMax i3x microplate reader.

### 4T07^MUC1, HER2^ mouse model

Experiments involving animals were approved under University of California, San Francisco, Institutional Animal Care and Use Committee protocol number AN179766. Animals were housed under the following conditions: 12-h dark/12-h light cycle, ambient temperature of 20–26 °C and relative humidity of between 30 and 70%. 4T07 cells expressing MUC1ΔCT were used to limit any possible cytoplasmic signaling^23^. 4T07^MUC1, HER2^ breast cancer cells expressing mApple luciferase and doxycycline-inducible MUC1ΔCT (1 × 10^6^) were seeded in the lungs of female syngeneic 8-week-old BALB/cJ mice by i.v. injection (tail vein). Cells were stimulated with 2 mg ml^−1^ doxycycline for 24 h to induce MUC1ΔCT expression before injection. Competent cell seeding of the lungs was assessed by BLI (IVIS In Vivo Imaging System) following i.p. injection of D-luciferin (150 mg per kg (body weight)) within 30 min of cell injection. Mice were given systemic treatments of PBS or 10 mg per kg (body weight) αHER2-eStcE by i.v. injection every 2 d for a total of seven doses. Tumor cell lung burden was assessed over time by additional BLI measurements of mice. BLI for independent images was calculated from total bioluminescence flux for the chest region of each mouse. At 15 d, animals were killed, and lungs were collected. Whole animal and gross lung weights were recorded as well as the number of detected lung surface lesions. Lungs were then formalin fixed and processed for paraffin embedding, and the average lung lesion area for each animal was determined from H&E-stained tissue sections. In a separate experiment, 4T07^MUC1, HER2^ cells were seeded as described above, and mice were immediately treated with one i.v. dose of PBS, 10 mg per kg (body weight) αHER2-eStcE or an equimolar dose of αHER2-eStcE^E447D^ or αGFP-eStcE (day 0). Two additional i.v. doses of each treatment were completed on days 2 and 4. Mice received a diet of gamma-irradiated doxycycline chow (Bio-Serv, 55829; 625 mg per kg (body weight)) for the duration of the experiment to maintain MUC1ΔCT expression. Mouse lung metastatic burden was monitored by BLI on days 0, 3, 5 and 8.

### EMT6^HER2^ mouse model

BALB/c mice were obtained from Janvier Laboratories and bred in-house at the University Hospital Basel, Switzerland. All mouse experiments were approved by the University Hospital Basel Ethical Committee (approval 2370 and 3036, Basel Stadt, Switzerland). Animals were housed under specific pathogen-free conditions. For tumor growth experiments, 8- to 12-week-old females were used, and 1 × 10^6^ EMT6^HER2^ cells were injected into the right mammary fat pad. For efficacy studies, four i.p. doses of PBS, 10 mg per kg (body weight) αHER2-eStcE or an equimolar quantity (2.8 nmol) of αHER2, αHER2-eStcE^E447D^ or αGFP-eStcE was administered every 2 d for a total of four doses once the tumor size reached an average size of 80−100 mm^3^. For analysis of tumor infiltration by flow cytometry, two i.p. doses of PBS, 10 mg per kg (body weight) αHER2-eStcE or an equimolar quantity of αHER2 were administered every 2 d for a total of two doses once the tumor size reached an average size of 80−100 mm^3^. Perpendicular tumor diameters were measured by caliper, and tumor volume was calculated according to the following formula: tumor volume (mm^3^) = (*d*^2^ × *D*)/2, where *d* and *D* are the shortest and longest diameters of the tumor (in millimeters), respectively. Mice were killed once the tumor size reached approximately 1,500 mm^3^ or when the mice developed ulcerated tumors that required euthanasia, and the animals were excluded from further analysis.

### IHC of lung tissues for the 4T07^MUC1, HER2^ mouse model

IHC was performed as previously described^75^ using antibodies specific to phospho-FAK Y397, cyclin D1 and phospho-(Ser/Thr)-Akt substrates (see Supplemental Table 5). Briefly, antigen retrieval was accomplished by boiling sections in 10 mM citrate buffer for 10 min. Following primary antibody incubation overnight at 4 °C, sections were incubated for 1 h with species-specific horseradish peroxidase (HRP)-conjugated secondary antibodies (ImmPRESS HRP goat anti-rabbit IgG polymer detection kit, Peroxidase, Vector Laboratories, MP-7452 and MP-7451) before developing positive staining with ImmPACT DAB substrate peroxidase (HRP, Vector Laboratories, SK-4105). Images of stained sections were acquired with an Olympus microscope (IX81) with a ×10 objective at 1.5× magnification.

### Quantitative histological analysis of lung tissues for the 4T07^MUC1, HER2^ mouse model

Analysis of IHC and H&E-stained lung tissue sections was performed using ImageJ and QuPath software, respectively. Specifically, an IHC profiler ImageJ plugin was used to quantify percent positive DAB staining area of lung metastases with selection for either cytoplasmic (phospho-FAK and phospho-(Ser/Thr)-Akt substrates) or nuclear (cyclin D1) staining^76^. Reported values correspond to the sum of high positive and positive DAB signal. To assess metastatic lesion area throughout the depth of mouse lung tissues, lungs were sectioned as two steps spaced 40 μm apart, with 15 sequential sections of 5 μm cut at each step. The top and bottom sections from each step were then stained with H&E (75 μm apart), and slides were scanned using a ZEISS Axio Scan.Z1 digital slide scanner equipped with CMOS and color cameras and ×10, ×20 and ×40 objectives. Percent area of lung metastasis for each section was determined in Qupath using the polygon tool to trace and annotate lung lesion area compared to whole tissue section area. The average values of two lung sections from each animal are presented.

### Analysis of in vivo mucin cleavage in the EMT6^HER2^ mouse model

For the preparation of single-cell suspensions, tumors were collected, and surgical specimens were mechanically dissociated and subsequently digested using Accutase (PAA Laboratories), collagenase IV (Worthington), hyaluronidase (Sigma) and DNase type IV (Sigma) for 1 h at 37 °C under constant agitation. Cell suspensions were filtered through a 70-μm mesh, 10 μl of CountBright Plus absolute counting beads (Invitrogen) was added, and samples were frozen at −80 °C. Single-cell suspensions were briefly thawed in a 37 °C water bath and immediately placed on ice. Cells were washed once and counted, and 1.9 × 10^6^−3 × 10^6^ cells were processed per biological sample. Cells were washed once with cold FACS buffer, treated with mouse BD Fc block in cold FACS buffer for 5 min on ice and immediately stained with Brilliant Violet 421 CD45 antibody (30-F11), Alexa Fluor 488 HER2 antibody (24D2) and Alexa Fluor 647 StcE^E447D^ (5 μg ml^−1^) in FACS buffer with 1:1,000 benzonase for 30 min on ice protected from light. UltraComp eBeads Plus Compensation Beads (Thermo Fisher Scientific) were stained in parallel for antibody single-color controls following the manufacturer’s recommendations. Cells were washed once with cold PBS and stained with 1:1,000 GloCell Fixable Viability Dye Violet 510 (STEMCELL Technologies) in PBS for 30 min on ice protected from light. ArC Amine Reactive Compensation Beads (Thermo Fisher Scientific) were stained in parallel for viability single-color controls following the manufacturer’s recommendations. Cells were washed once with cold FACS buffer with 2 mM EDTA and resuspended in cold FACS buffer with 2 mM EDTA, and an average of 560,000 live single cells were analyzed using a MACSQuant Analyzer 10 flow cytometer (Miltenyi Biotec). EMT6^HER2^ and immune cells were gated from live single cells as shown in Supplementary Fig. 11 using fluorescence minus one controls. Correct gating of these populations was confirmed with EMT6^HER2^ and commercial mouse PBMCs (IQ Biosciences), which were stained and analyzed in parallel with the tumor samples.

### Flow cytometry analysis of tumor-infiltrating immune cells

Thawed single-cell suspensions were stained with antibodies shown in Supplementary Table 6 and analyzed on a Cytek Aurora instrument. Live single cells were gated for different immune subsets as diagrammed in Supplementary Fig. 12a. For t-distributed stochastic neighbor embedding (t-SNE) analysis, live single CD45^+^ cells were randomly downsampled using the FlowJo DownSample v3.3.1 plugin. t-SNE analysis was performed using the FlowJo t-SNE plugin once on the concatenated files using all compensated fluorophores (default settings: learning configuration = opt-SNE, iterations = 1,000, perplexity = 30, learning rate = 6,996, KNN algorithm = exact(vantage point tree), gradient algorithm = Barnes-Hut) on ~100,000 total cells evenly distributed between treatment groups and evenly distributed between biological replicates in each treatment group. For analysis of activation states, gates for positive staining were defined using unstained samples and were kept consistent for all immune subsets (Supplementary Fig. 12b).

### Reporting summary

Further information on research design is available in the Nature Portfolio Reporting Summary linked to this article.

## Extended Data

## Supplementary Material

Suplementary information

supplementary information 2

video 1

video 2

video 3

data 2

data 3

data 4

data 1

## Data Availability

Data supporting the findings of this study are available within the article and its Supplementary Information or from the corresponding author upon reasonable request. MS proteomics data have been deposited to the ProteomeXchange Consortium via the PRIDE^77^ partner repository with the dataset identifier PXD042243.
