# Supplementary material for: Design of a mucin-selective protease for targeted degradation of cancer-associated mucins
Source: Nat Biotechnol. Author manuscript; Available in PMC 2024 Apr 17. (PMC11018308; doi:10.1038/s41587-023-01840-6)
Supplement: Suplementary information [file NIHMS1939299-supplement-Suplementary_information.pdf]

nature biotechnology

Article https://doi.org/10.1038/s41587-023-01840-6

Design of a mucin-selective protease for targeted degradation of cancer-associated mucins

In the format provided by the authors and unedited

Supplementary Information Table of Contents

Supplementary Figures 1-12
Supplemental Tables 5-8

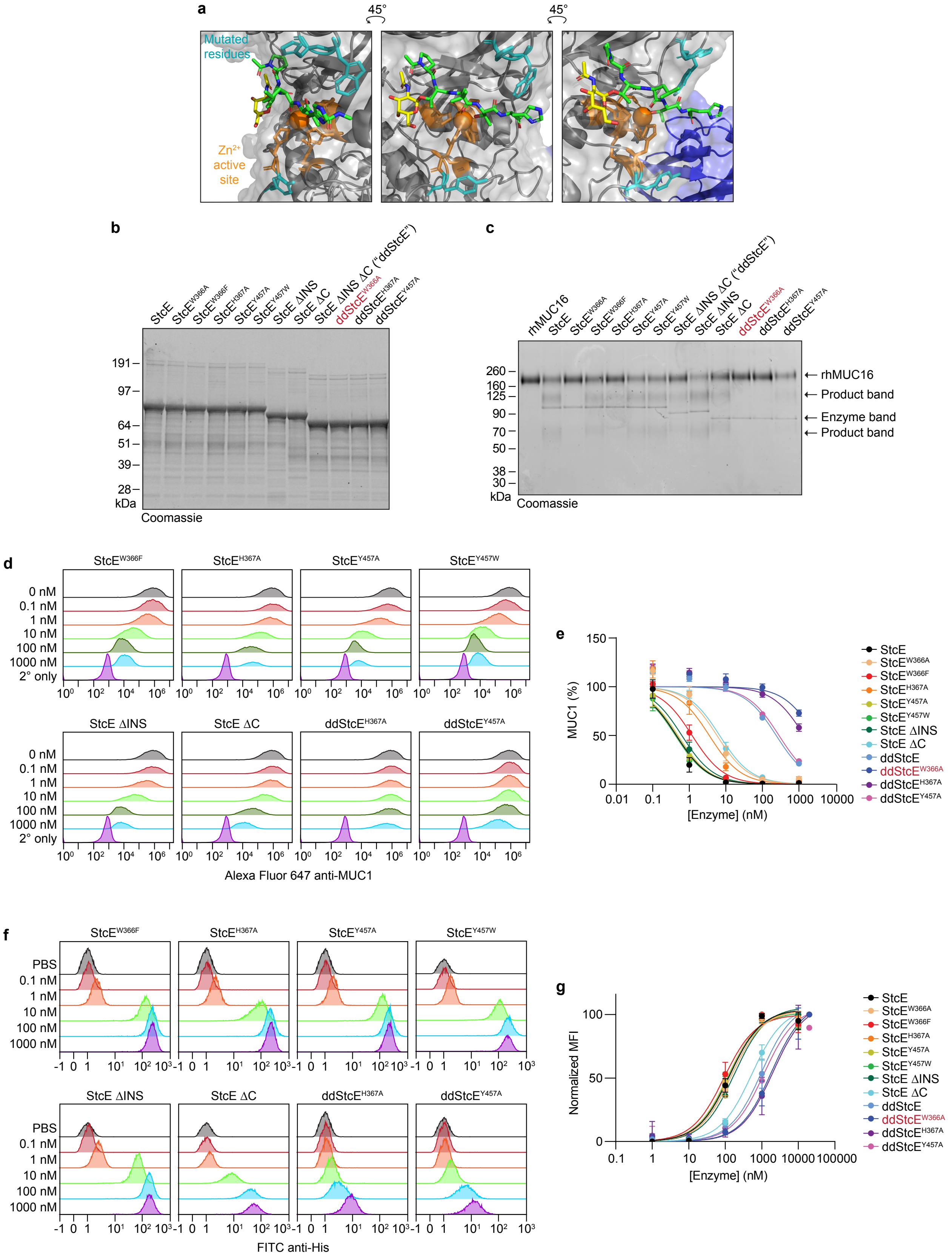
a
45°
45°
Mutated residues
Zn2+ active site
b
StcE
StcEW366A
StcEW366F
StcEH367A
StcEY457A
StcEY457W
StcE ΔINS
StcE ΔC
StcE ΔINS ΔC ("ddStcE")
ddStcEW366A
ddStcEH367A
ddStcEY457A
191
97
64
51
39
28
kDa
Coomassie
c
rhMUC16
StcE
StcEW366A
StcEW366F
StcEH367A
StcEY457A
StcEY457W
StcE ΔINS ΔC ("ddStcE")
StcE ΔINS
StcE ΔC
ddStcEW366A
ddStcEH367A
ddStcEY457A
260
160
125
90
70
50
38
30
kDa
Coomassie
← rhMUC16
← Product band
← Enzyme band
← Product band
d
StcEW366F
StcEH367A
StcEY457A
StcEY457W
StcE ΔINS
StcE ΔC
ddStcEH367A
ddStcEY457A
0 nM
0.1 nM
1 nM
10 nM
100 nM
1000 nM
2° only
10^0 10^2 10^4 10^6
Alexa Fluor 647 anti-MUC1
e
MUC1 (%)
150
100
50
0
0.01 0.1 1 10 100 1000 10000
[Enzyme] (nM)
StcE
StcEW366A
StcEW366F
StcEH367A
StcEY457A
StcEY457W
StcE ΔINS
StcE ΔC
ddStcE
ddStcEW366A
ddStcEH367A
ddStcEY457A
f
StcEW366F
StcEH367A
StcEY457A
StcEY457W
StcE ΔINS
StcE ΔC
ddStcEH367A
ddStcEY457A
PBS
0.1 nM
1 nM
10 nM
100 nM
1000 nM
-1 0 1 10^1 10^2 10^3
FITC anti-His
g
Normalized MFI
100
50
0
0.1 1 10 100 1000 10000 100000
[Enzyme] (nM)
StcE
StcEW366A
StcEW366F
StcEH367A
StcEY457A
StcEY457W
StcE ΔINS
StcE ΔC
ddStcE
ddStcEW366A
ddStcEH367A
ddStcEY457A

Supplementary Fig. 1. Design, expression, and characterization of engineered StcE mutants.

a, Docking of glycopeptide Ac-P(GalNAc)TLTH-*N*Me into the structure of StcE determined using AlphaFold[65]. The INS domain is shown in blue. The Zn^{2+} active site is depicted in orange while mutated residues are shown in teal. The glycopeptide backbone is shown in green and the GalNAc sugar is depicted in yellow.
b, SDS-PAGE of purified StcE and StcE mutants.
c, Digestion of rhMUC16 with 50 nM StcE or StcE mutants at 37 °C for 1 hour.
d, Representative flow plots related to Fig. 2e-f showing surface MUC1 levels of HeLa cells treated with StcE variants at indicated concentrations.
e, MUC1 cleavage curves for StcE and StcE mutants corresponding to mean fluorescence intensity from Fig. 2e-f and (**d**) (*n*=3 biologically independent replicates).
f, Representative flow plots related to Fig. 2g-h depicting cell surface binding of StcE variants on HeLa cells measured by anti-His staining (*n*=3 biological replicates).
g, Binding curves for StcE and StcE mutants corresponding to normalized mean fluorescence intensity from Fig. 2g-h and (**f**) (*n*=3 biologically independent replicates).
Data are mean ± s.d.

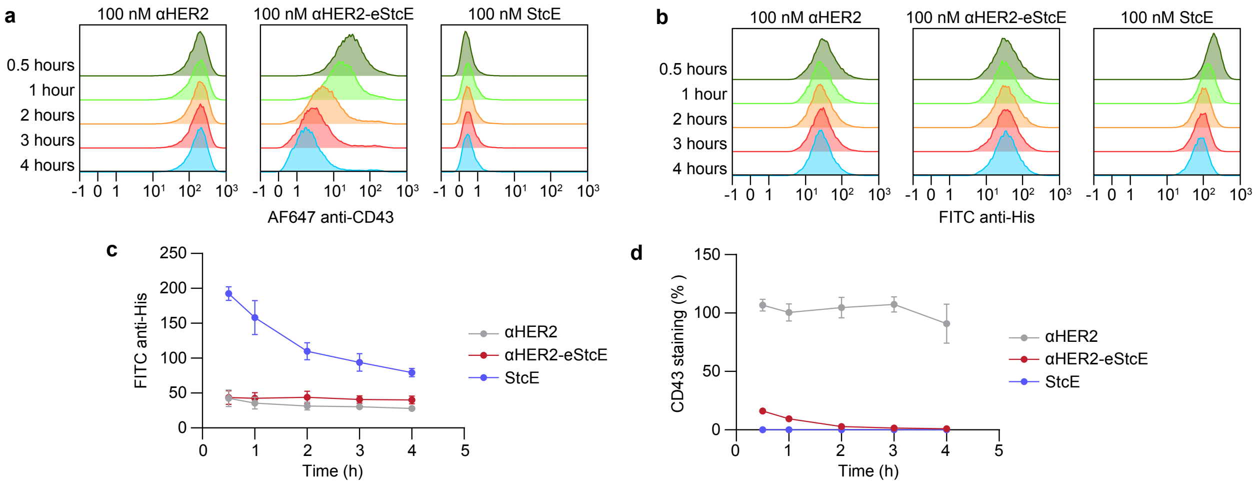

Supplementary Fig. 2. αHER2-eStcE does not need to bind mucins stably in order to deplete cellular mucins.

a-b, Representative flow plots showing the change in CD43 cell surface levels (**a**) and binding (**b**) to K562^{HER2} cells following different incubation times with 100 nM of αHER2, αHER2-eStcE, or StcE.

c-d, Time-dependent CD43 cleavage determined via quantification of the normalized median fluorescence intensities (**c**) and plot of the median fluorescence intensities depicting αHER2, αHER2-eStcE, or StcE cell surface residency (**d**) from (**a-b**) (*n*=3 biologically independent replicates).

Data are mean ± s.d.

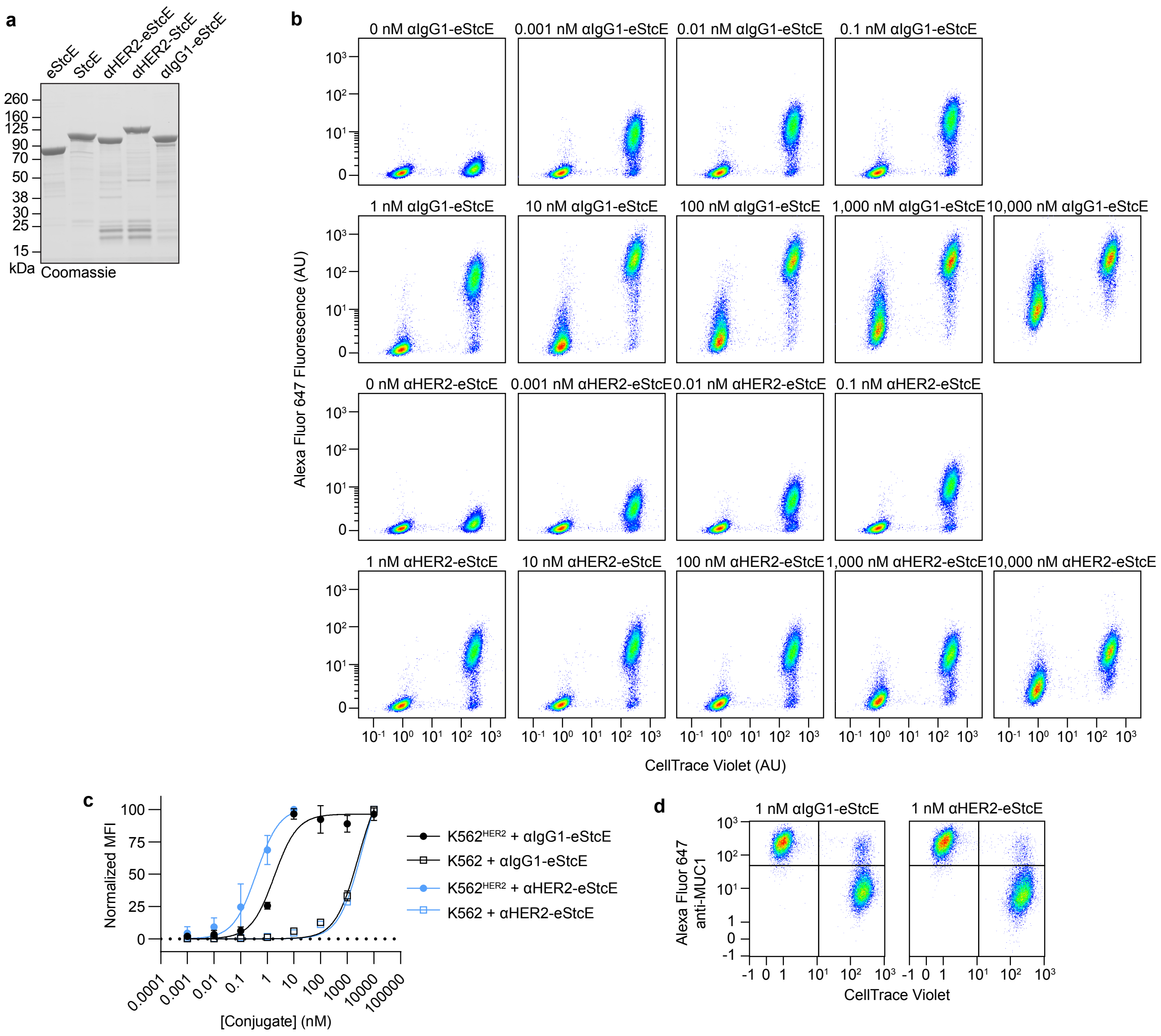

Supplementary Fig. 3. Validation of αIgG1-eStcE.

a, SDS-PAGE of purified αHER2-eStcE, αHER2-StcE, and αIgG1-eStcE.

b, Flow plots depicting specific binding of αIgG1-eStcE to mouse IgG1 antibodies. CellTrace Violet stained K562^{HER2} cells were mixed with unstained K562 cells, stained with primary mouse IgG1 anti-HER2 and secondary Alexa Fluor 647-labeled αIgG1-eStcE or no primary and Alexa Fluor 647-labeled αHER2-eStcE.

c, Cell surface binding curves derived from normalized median fluorescence intensities from (**b**) (*n*=2 biologically independent replicates).

d, Flow plots of αIgG1-eStcE activity in a mixed cell cutting assay. Mixed K562 cells and K562^{HER2} cells were treated overnight with αHER2-eStcE or 10 μg/mL anti-HER2 mouse IgG1 and αIgG1-eStcE.

Data are mean ± s.d.

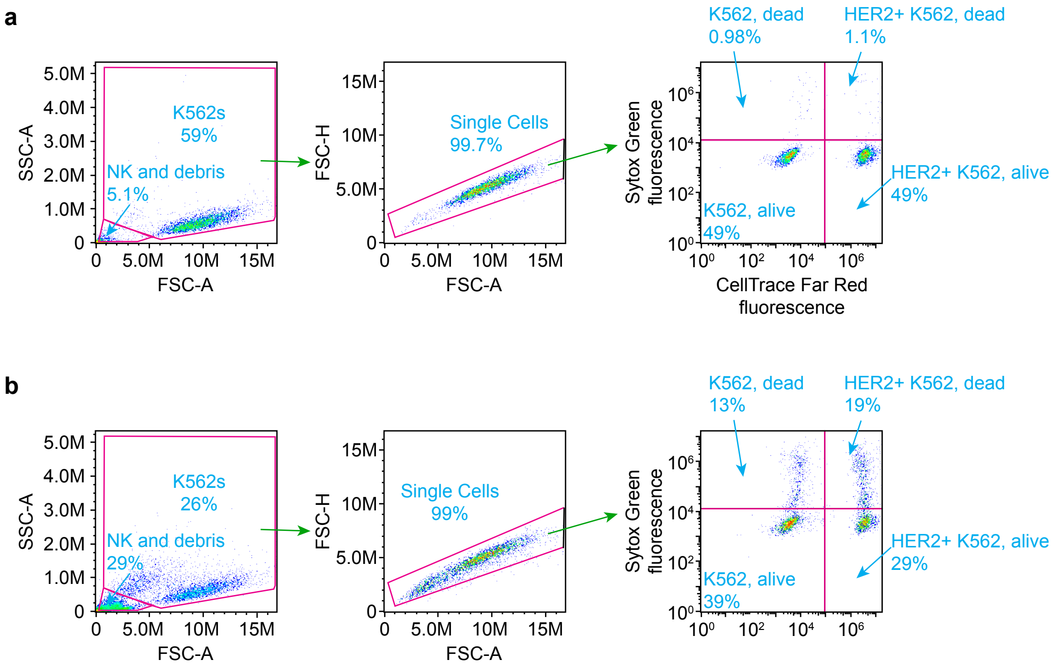

Supplementary Fig. 4. Gating strategy for mixed NK cell killing assay.
a-b, Gating strategy used to define the populations in Fig. 4d using a representative 10 nM αHER2-eStcE treated K562$^{\pm HER2}$ sample (**a**) and a representative 10 nM αHER2-eStcE and NK cell treated K562$^{\pm HER2}$ sample (**b**). K562$^{\pm HER2}$ were gated from NK cells using FSC-A vs SSC-A given the different sizes of the two cell populations. Values shown on the graph are the percentage of cells from the parent population in each gate from these representative replicates.

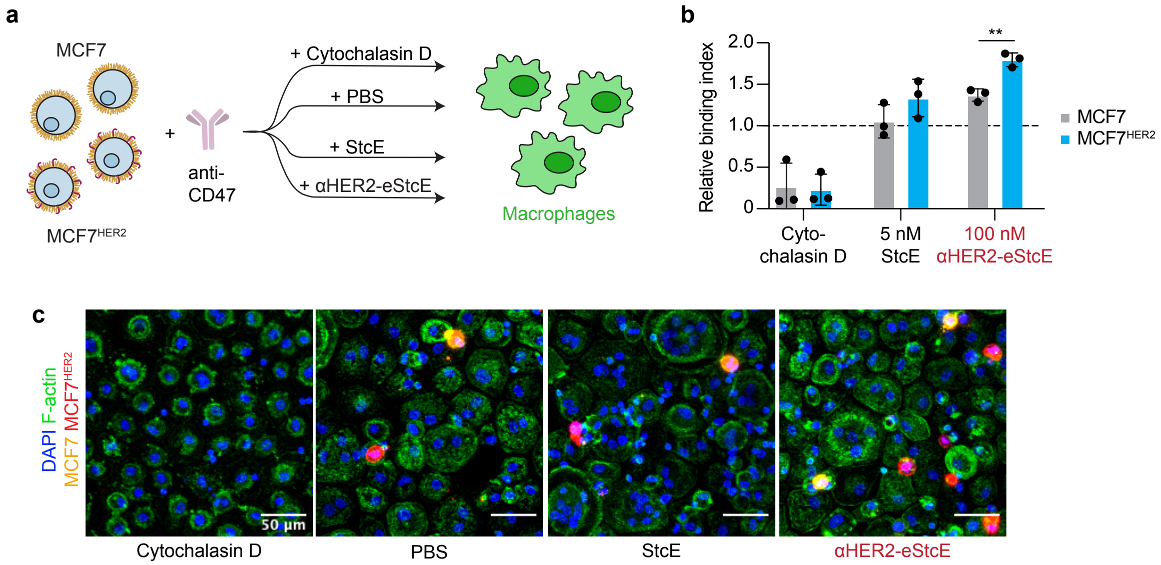

Supplementary Fig. 5. αHER2-eStcE potentiates macrophage phagocytosis in a mixed cell assay.

a, Setup for mixed cell macrophage phagocytosis assay using MCF7$^{\pm HER2}$ cells.

b, Relative binding index of MCF7$^{\pm HER2}$ cells treated with the phagocytosis inhibitor cytochalasin D, StcE, or αHER2-eStcE (*n*=3 biologically independent replicates).

c, Representative confocal microscopy images used for (**b**).

Data are mean ± s.d. *P*-values were determined using multiple unpaired two-tailed t-tests with two-stage Benjamini, Kreiger, and Yekutieli false discovery rate correction. *$p < 0.05$, **$p < 0.005$, ***$p < 0.0005$.

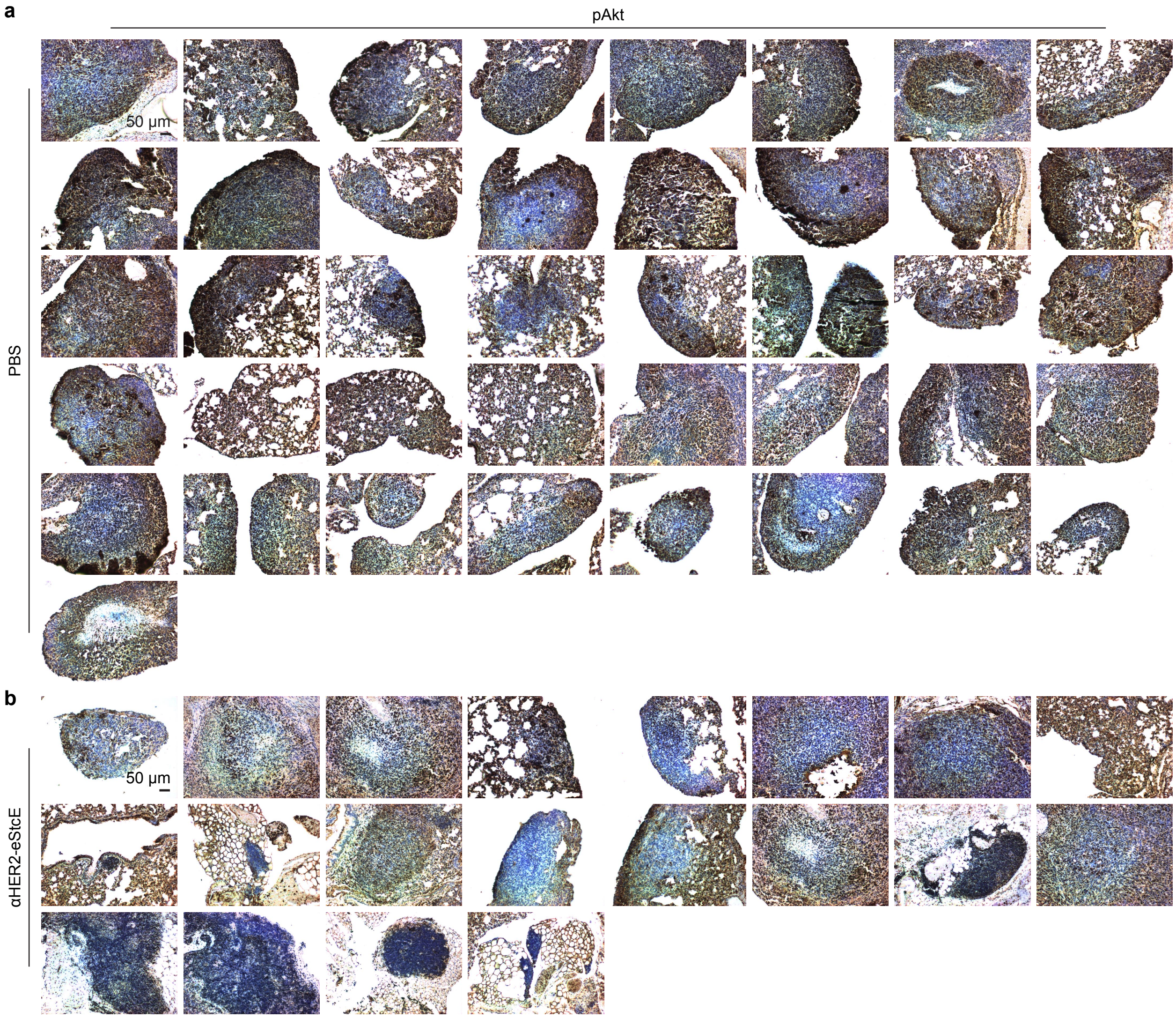

Supplementary Fig. 6. In the 4T07$^{MUC1, HER2}$ murine model of breast cancer progression, αHER2-eStcE reduces the prosurvival mechanosignaling marker, pAkt[23].

a, pAkt immunohistochemistry of lungs from PBS-treated animals described in Fig. 4e. (*n*=41 unique fields of view from 7 animals).

b, pAkt immunohistochemistry of lungs from αHER2-eStcE treated animals described in Fig. 4e. (*n*=20 unique fields of view from 7 animals).

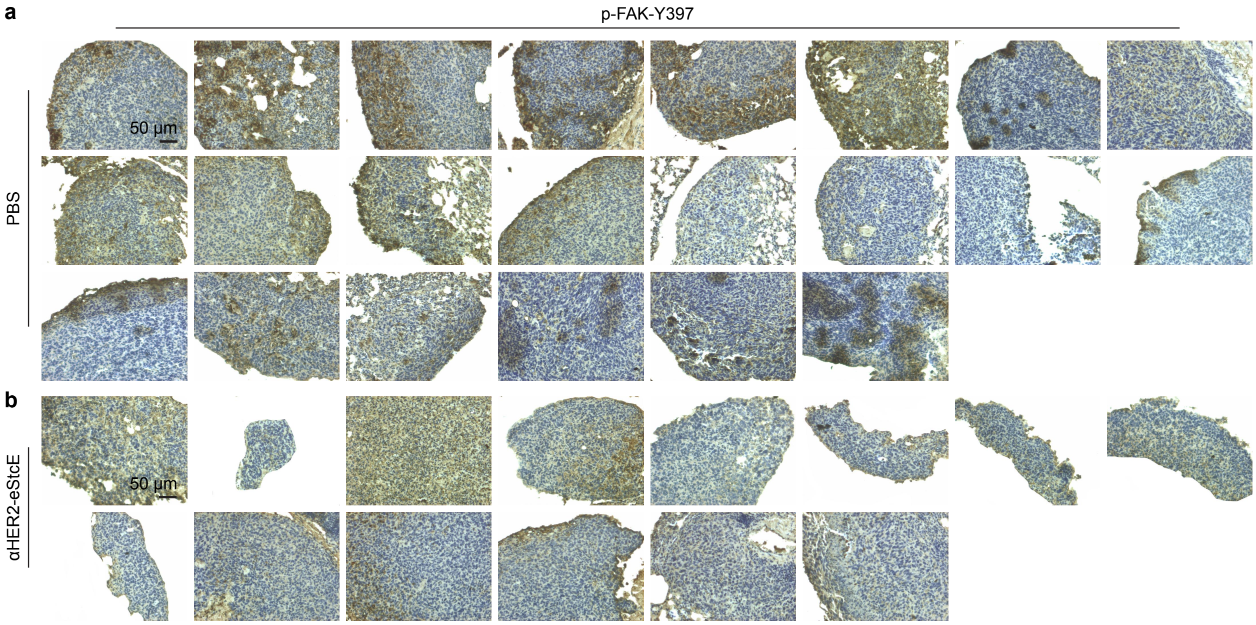

Supplementary Fig. 7. In the 4T07$^{MUC1,\ HER2}$ murine model of breast cancer progression, αHER2-eStcE reduces the prosurvival mechanosignaling marker, p-FAK-Y397[23].

a, p-FAK-Y397 immunohistochemistry of lungs from PBS-treated animals described in Fig. 4e. (*n*=22 unique fields of view from 7 animals).

b, p-FAK-Y397 immunohistochemistry of lungs from αHER2-eStcE treated animals described in Fig. 4e. (*n*=14 unique fields of view from 7 animals).

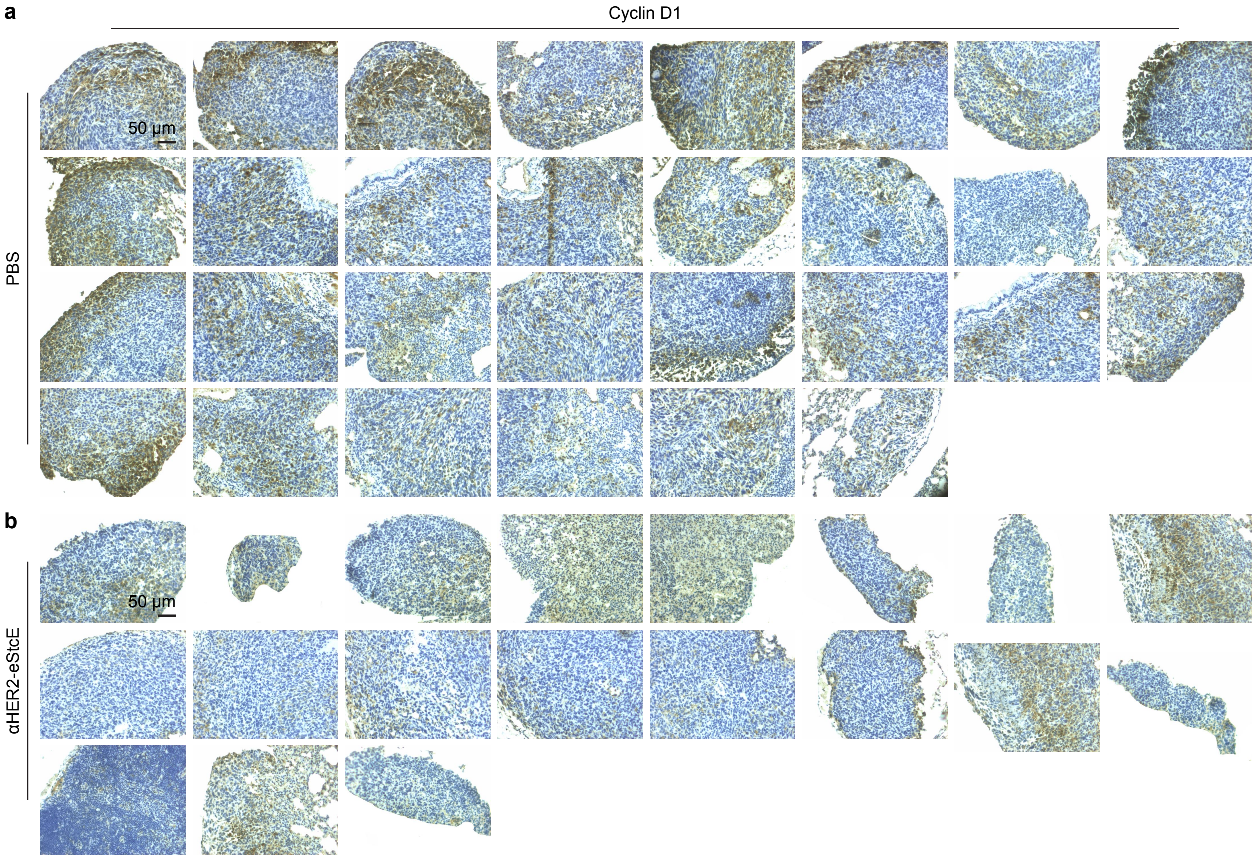

Supplementary Fig. 8. In the 4T07$^{MUC1,\ HER2}$ murine model of breast cancer progression, αHER2-eStcE reduces, cyclin D1, a protein required for cell cycle progression[23].

a, Cyclin D1 immunohistochemistry of lungs from PBS-treated animals described in Fig. 4e. (*n*=30 unique fields of view from 7 animals).

b, Cyclin D1 immunohistochemistry of lungs from αHER2-eStcE treated animals described in Fig. 4e. (*n*=19 unique fields of view from 7 animals).

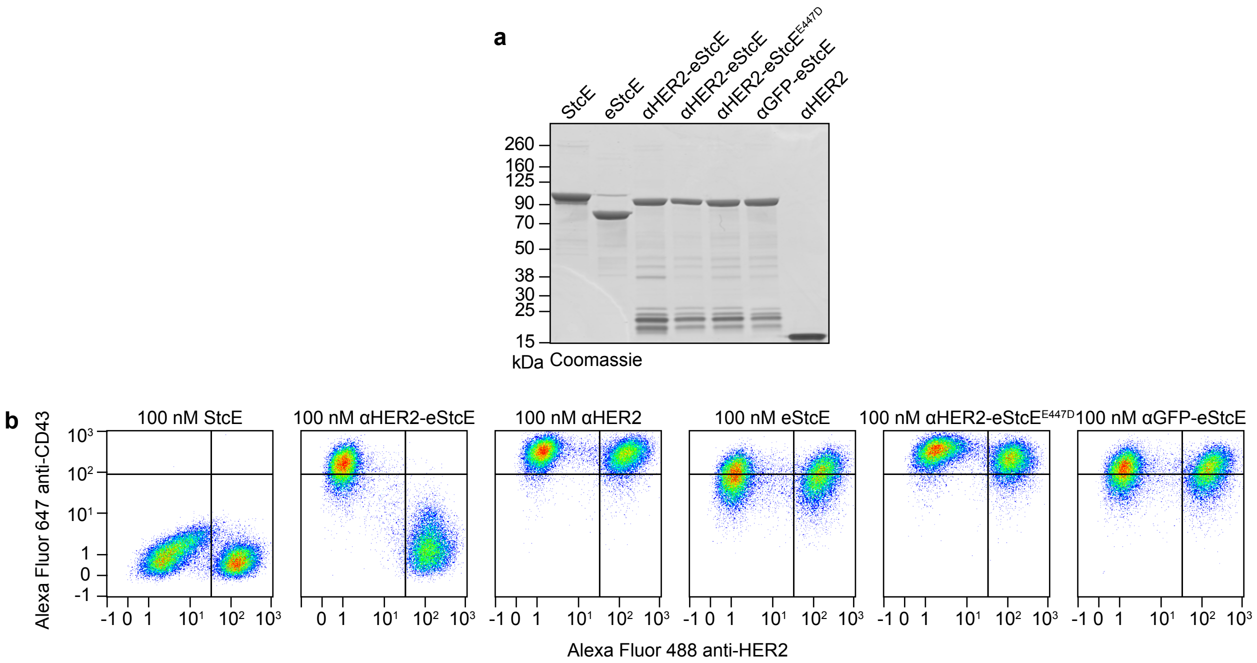

Supplementary Fig. 9. Generation and validation of αGFP-eStcE and αHER2-eStcE^{E447D}.

a, SDS-PAGE of purified αHER2-eStcE^{E447D}, αGFP-eStcE, and αHER2. The two αHER2-eStcE lanes represent different purification batches.

b, Flow plots depicting surface CD43 levels of mixed K562$^{\pm HER2}$ cells treated with StcE, αHER2, αHER2-eStcE, αGFP-eStcE, or αHER2-eStcE^{E447D} overnight.

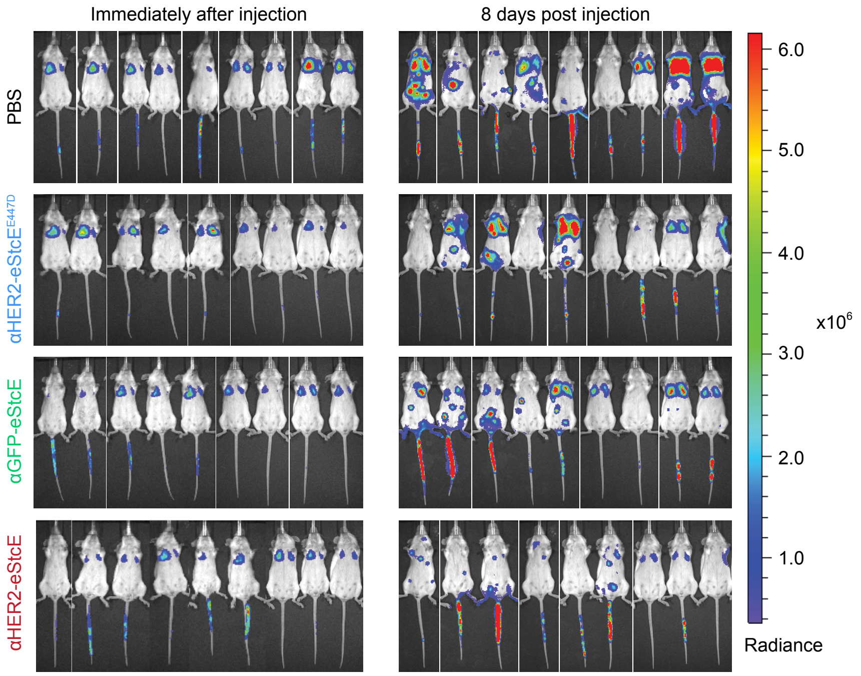

Supplementary Fig. 10. Bioluminescent imaging of animals described in Extended Data, Fig. 9k.

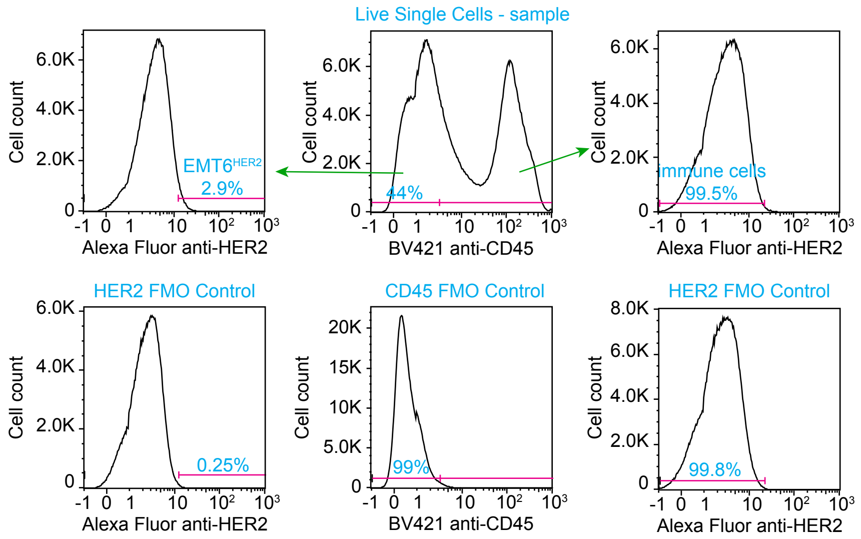

Supplementary Fig. 11. Gating strategy for EMT6^{HER2} and immune cells, related to Extended Data Fig. 10e-f. Fluorescence minus one controls (FMO) were used to define negative staining gates. The values given are the percentage of cells from the parent population in each gate from these representative replicates.

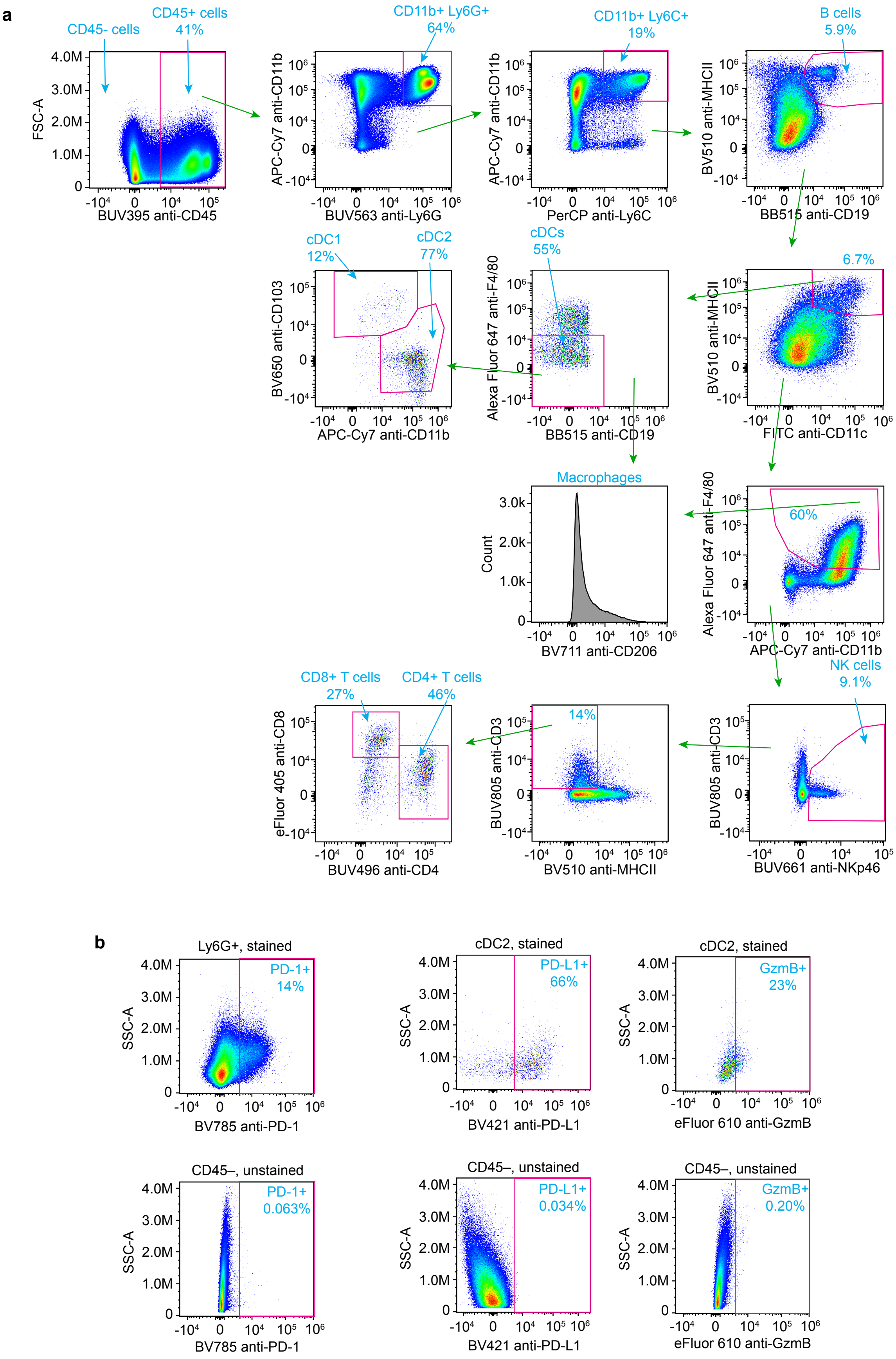
a
CD45- cells
CD45+ cells 41%
FSC-A
BUV395 anti-CD45
CD11b+ Ly6G+ 64%
APC-Cy7 anti-CD11b
BUV563 anti-Ly6G
CD11b+ Ly6C+ 19%
PerCP anti-Ly6C
B cells 5.9%
BV510 anti-MHCII
BB515 anti-CD19
cDC1 12%
cDC2 77%
BV650 anti-CD103
cDCs 55%
Alexa Fluor 647 anti-F4/80
6.7%
FITC anti-CD11c
Macrophages
Count
BV711 anti-CD206
60%
CD8+ T cells 27%
CD4+ T cells 46%
eFluor 405 anti-CD8
BUV496 anti-CD4
14%
BUV805 anti-CD3
NK cells 9.1%
BUV661 anti-NKp46
b
Ly6G+, stained
PD-1+ 14%
SSC-A
BV785 anti-PD-1
cDC2, stained
PD-L1+ 66%
BV421 anti-PD-L1
cDC2, stained
GzmB+ 23%
eFluor 610 anti-GzmB
CD45–, unstained
PD-1+ 0.063%
CD45–, unstained
PD-L1+ 0.034%
CD45–, unstained
GzmB+ 0.20%

Supplementary Fig. 12. Gating strategy for EMT6^{HER2} immune subset profiling.
a, Gating strategy used to define different immune subsets in Extended Data Fig. 10g-o from live single cells. Plots are from a representative αHER2-eStcE treated mouse, with the percentages of cells within each gate of the parent population from this representative sample shown.
b, Defining gates for positive PD-1, PD-L1, and GzmB staining in Extended Data Fig. 10l, n-o with immune subsets from the same mouse as in (**a**). Top plots show gates on stained populations and bottom plots show gates on a fully unstained sample. The values given are the percentage of cells from the parent population in each gate from the representative samples shown.

Supplemental Table 5. Antibody clones and working concentrations.

Antibody target	Color	Vendor	Clone	Cat. Number	Dilution/ concentration
HER2	AF488/ AF647	BioLegend	24D2	324410/ 324412	1:200
CD43	AF647	Novus Biologics	MEM-59	NBP2-62227AF647	1:1000 (flow cytometry on BD Accuri C6 plus) 2.5 µg/mL (flow cytometry on MACSQuant10)
MUC1	Purified	EMD Millipore	214D4	05652	1:200 (flow cytometry), 1:1000 (Western blot)
Affinipure Goat Anti-Mouse IgG	AF647	Jackson ImmunoResearch	polyclonal	115605003	1:375
His	FITC	Miltenyi Biotec	GG11-8F3.5.1	130123547	1:50
MUC1	Purified	Cell Signaling Technology	VU4H5	4538S	1:200
MUC16	Purified	Abcam	X75	ab1107	1:1000
CD47	Purified	BioXCell	MIAP410	BE0283	20 µg/mL
CD45	BV421	BioLegend	30-F11	103134	1:80
Mouse BD Fc Block	Purified	BD Biosciences	2.4G2	553142	1:50
Phospho-FAK tyrosine 397	Purified	Invitrogen	31H5L17	700255	1:100
Cyclin D1	Purified	Cell Signaling Technology	polyclonal	2922	1:200
Phospho-(Ser/Thr) Akt Substrate Antibody	Purified	Cell Signaling Technology	polyclonal	9611	1:400

Supplemental Table 6. Flow panel antibodies related to Extended Data Fig. 10g-o and Supplementary Fig. 12.

Antibody target	Color	Vendor	Clone	Cat. Number	Dilution
CD45	BUV395	BD Biosciences	30-F11	564279	1:100
Live-dead	Zombie UV	BioLegend		423107	1:100
CD4	BUV496	BD Biosciences	?	93937	1:100
Ly-6G	BUV563	BD Biosciences	1A8	612921	1:200
NKp46	BUV661	BD Biosciences	29A1.4	741678	1:70
CD3	BUV805	BD Biosciences	145-2C11	749276	1:70
PD-L1	BV421	BioLegend	10F.9G2	124315	1:150
LFA-1	SB436	eBioscience	M17/4	62011180	1:100
CD8	eFluor 450	eBioscience	53-6.7	480081	1:100
MHCII	BV510	BioLegend	M5/114.15.2	107635	1:300
CD80	BV605	BioLegend	16-10A1	104729	1:70
CD103	BV650	BD Biosciences	2E7	748256	1:70
CD206	BV711	BioLegend	C068C2	141727	1:100
PD-1	BV785	BioLegend	29F.1A12	135225	1:100
CD19	BB515	BD Biosciences	1D3	564509	1:100
CD11c	FITC	BioLegend	N418	117306	1:100
Ly-6C	PerCP	BioLegend	HK1.4	128028	1:200
Tim-3	BB700	BD Biosciences	5D12/TIM-3	747619	1:100
Siglec E	PE	BioLegend	M1304A01	677104	1:50
CD25	PE-Cy5.5	eBioscience	PC61.5	35025182	1:100
F4/80	AF647	BioLegend	BM8	123122	1:100
CD11b	APC-Cy7	BioLegend	M1/70	101226	1:100
Ki67	AF532	eBioscience	SolA15	58569882	1:200
TCF-7	AF700	R&D Systems	812145	FAB8224N	1:100
GzmB	PE-eFluor610	eBioscience	NGZB	61889882	1:100
FoxP3	APC	eBioscience	FJK-16s	17577382	1:100

Supplemental Table 7. Sample IDs and TMT labeling for TAILS MS.

Channel	Sample	Channel	Sample
126C	PBS 1	127N	StcE 1
127C	PBS 2	128N	StcE 2
128C	PBS 3	129N	StcE 3
129C	PBS 4	130N	StcE 4
130C	eStcE 1	131N	αHER2-eStcE 1
131C	eStcE 2	132N	αHER2-eStcE 2
132C	eStcE 3	133N	αHER2-eStcE 3
133C	eStcE 4	134N	αHER2-eStcE 4

Supplemental Table 8. Primary antibodies used with αIgG1-eStcE.

Target name	Antibody clone number	Vendor	Mucin?	Mucinome enrichment score	Concentration of antibody for cutting experiment (μg/mL)	Maximum MFI observed in binding assay (AU)
MUC1	214D4	EMD Millipore	Yes	9.41	5	327.8
CD43	CD43-10G7	BioLegend	Yes	8.52	10	160.4
CD30	BY88	BioLegend	Yes	7.18	5	9.8
CD236	QA20A41	BioLegend	No	8.48	5	188.5
CD46	TRA-2-10	BioLegend	No	7.26	2.5	64.9
HER2	24D2	BioLegend	No	--	2.5	310.6
CD98	MEM-108	BioLegend	No	-4.49	10	234
Annexin A1 (ANXA1)	74/3	BioLegend	No	-7.44	20	1.3
CD29	P5D2	BioLegend	No	-4.17	2.5	23.7
Isotype	MOPC-21	BioLegend			20	0.1
