# Supplementary material for: Design of a mucin-selective protease for targeted degradation of cancer-associated mucins
Source: Nat Biotechnol. Author manuscript; Available in PMC 2024 Apr 17. (PMC11018308; doi:10.1038/s41587-023-01840-6)
Supplement: supplementary information 2 [file NIHMS1939299-supplement-supplementary_information_2.pdf]

nature research

Corresponding author(s): Carolyn R. Bertozzi

Last updated by author(s): May 18, 2023

Reporting Summary

Nature Research wishes to improve the reproducibility of the work that we publish. This form provides structure for consistency and transparency in reporting. For further information on Nature Research policies, see our Editorial Policies and the Editorial Policy Checklist.

Statistics

For all statistical analyses, confirm that the following items are present in the figure legend, table legend, main text, or Methods section.

n/a	Confirmed	
☐	☒	The exact sample size (*n*) for each experimental group/condition, given as a discrete number and unit of measurement
☐	☒	A statement on whether measurements were taken from distinct samples or whether the same sample was measured repeatedly
☐	☒	The statistical test(s) used AND whether they are one- or two-sided<br>*Only common tests should be described solely by name; describe more complex techniques in the Methods section.*
☒	☐	A description of all covariates tested
☐	☒	A description of any assumptions or corrections, such as tests of normality and adjustment for multiple comparisons
☐	☒	A full description of the statistical parameters including central tendency (e.g. means) or other basic estimates (e.g. regression coefficient) AND variation (e.g. standard deviation) or associated estimates of uncertainty (e.g. confidence intervals)
☐	☒	For null hypothesis testing, the test statistic (e.g. *F*, *t*, *r*) with confidence intervals, effect sizes, degrees of freedom and *P* value noted<br>*Give P values as exact values whenever suitable.*
☒	☐	For Bayesian analysis, information on the choice of priors and Markov chain Monte Carlo settings
☒	☐	For hierarchical and complex designs, identification of the appropriate level for tests and full reporting of outcomes
☒	☐	Estimates of effect sizes (e.g. Cohen's *d*, Pearson's *r*), indicating how they were calculated

Our web collection on statistics for biologists contains articles on many of the points above.

Software and code

Policy information about availability of computer code

Data collection	Protein sequences for StcE, αHER2-eStcE, and eStcE-αHER2 were used as inputs for ColabFold (https://colab.research.google.com/github/sokrypton/ColabFold/blob/main/AlphaFold2.ipynb#scrollTo=kOblAo-xetgx) to obtain model structures. The docked glycopeptide-StcE model was generated using Molecular Operating Environment (MOE). Flow cytometry data were collected using MACSQuant Analyzer 10 and BD Accuri C6 plus flow cytometers. SDS-PAGE gels were imaged using an Odyssey CLx Near-Infrared Fluorescence Imaging System (LI-COR Biosciences). MS data were collected using an Orbitrap Fusion Tribrid mass spectrometer.
Data analysis	All flow cytometry data were analyzed using FlowJo v. 10.0. Lethal fraction scores for cell death assays were calculated in Excel (Microsoft Corp., v.16.72). The imaging software Imaris was used to analyze macrophage phagocytosis results. Analysis of SDS-PAGE gels and tissue sections were performed using ImageJ and QuPath software. HCD-pd-ETD raw files were searched using Byonic by ProteinMetrics and O-Pair in MetaMorpheus (0.0.307 and 0.0.308). Peptides were manually validated and/or sequenced using Xcalibur software. Raw TAILS mass spectrometry data files were processed using MaxQuant. Statistical analyses were performed using Graphpad Prism version 9.0. Molecular graphics were generated using PyMOL v2.5.2.

For manuscripts utilizing custom algorithms or software that are central to the research but not yet described in published literature, software must be made available to editors and reviewers. We strongly encourage code deposition in a community repository (e.g. GitHub). See the Nature Research guidelines for submitting code & software for further information.

Data

Policy information about availability of data

All manuscripts must include a data availability statement. This statement should provide the following information, where applicable:

- Accession codes, unique identifiers, or web links for publicly available datasets
- A list of figures that have associated raw data
- A description of any restrictions on data availability

Data supporting the findings of this study are available within the article and its supplementary information or from the corresponding author upon reasonable request. The mass spectrometry proteomics data have been deposited to the ProteomeXchange Consortium via the PRIDE partner repository with the dataset identifier PXD042243.

Field-specific reporting

Please select the one below that is the best fit for your research. If you are not sure, read the appropriate sections before making your selection.

☒ Life sciences ☐ Behavioural & social sciences ☐ Ecological, evolutionary & environmental sciences

For a reference copy of the document with all sections, see nature.com/documents/nr-reporting-summary-flat.pdf

Life sciences study design

All studies must disclose on these points even when the disclosure is negative.

Sample size	No sample size calculations were performed. The sample size (n) for each experiment is provided in the corresponding figure caption. Sample sizes were chosen to support meaningful conclusions.
Data exclusions	No data were excluded.
Replication	All attempts at replication were successful and are noted in the figure legends.
Randomization	Mice and all biological samples were allocated randomly into different experimental groups.
Blinding	Experiments were not blinded because data collection and analysis were performed by the same investigator.

Reporting for specific materials, systems and methods

We require information from authors about some types of materials, experimental systems and methods used in many studies. Here, indicate whether each material, system or method listed is relevant to your study. If you are not sure if a list item applies to your research, read the appropriate section before selecting a response.

Materials & experimental systems

n/a	Involved in the study	
☐	☒	Antibodies
☐	☒	Eukaryotic cell lines
☒	☐	Palaeontology and archaeology
☐	☒	Animals and other organisms
☒	☐	Human research participants
☒	☐	Clinical data
☒	☐	Dual use research of concern

Methods

n/a	Involved in the study	
☒	☐	ChIP-seq
☐	☒	Flow cytometry
☒	☐	MRI-based neuroimaging

Antibodies

Antibodies used	All antibodies used are listed in Supplemental Tables 5, 6 and 8.
Validation	Antibodies used in this study were validated by the manufacturer for the specific applications (flow cytometry, Western blot, and immunohistochemistry) and have been used repeatedly in the literature. Examples are provided on the manufacturer's website showing positive and negative controls.

Eukaryotic cell lines

Policy information about cell lines

Cell line source(s)	MCF10A cells were obtained from M. Paszek (Cornell University). EMT6 cells were obtained from H. Läubli (University of Basel). 4T07 cells were obtained from V. Weaver (UCSF). K562, CCRF-CEM, HeLa, CCRF-HSB-2, MCF7, OVCAR-3, and HEK-293T cells were obtained from ATCC.
Authentication	These cell lines have not been authenticated.
Mycoplasma contamination	All cell lines tested negative for mycoplasma contamination.
Commonly misidentified lines (See ICLAC register)	No commonly misidentified cell lines were used.

Animals and other organisms

Policy information about studies involving animals; ARRIVE guidelines recommended for reporting animal research

Laboratory animals	12-week-old male BALB/cJ, 7-12-week-old female BALB/cJ, and 10-week-old male C57BL/6 mice were used in this study.
Wild animals	This study did not involve wild animals.
Field-collected samples	This study did not involve field-collected samples.
Ethics oversight	Experiments involving animals were approved under Stanford APLAC protocol no. 31511 and no. 10266, UCSF IACUC protocol no. AN179766, and University Hospital Basel Ethical Committee Approval 2370 and 3036.

Note that full information on the approval of the study protocol must also be provided in the manuscript.

Flow Cytometry

Plots

Confirm that:

☒ The axis labels state the marker and fluorochrome used (e.g. CD4-FITC).

☒ The axis scales are clearly visible. Include numbers along axes only for bottom left plot of group (a 'group' is an analysis of identical markers).

☒ All plots are contour plots with outliers or pseudocolor plots.

☒ A numerical value for number of cells or percentage (with statistics) is provided.

Methodology

Sample preparation	Described in methods.
Instrument	MACSQuant Analyzer 10, BD Accuri C6 Plus, Cytek Aurora
Software	FlowJo v. 10.0
Cell population abundance	N/A
Gating strategy	Unless otherwise noted, different populations were separated using fluorochrome staining from live single cells. Cells were isolated from debris using FSC-A/SSC-A, and single cells were gated using FSC-A/FSC-H. Live cells were selected using the lower (unstained) viability dye population, and resulting single, live cells were analyzed.

☒ Tick this box to confirm that a figure exemplifying the gating strategy is provided in the Supplementary Information.
